# Genome-Wide Identification and Characterization of the* LRR-RLK* Gene Family in Two* Vernicia* Species

**DOI:** 10.1155/2015/823427

**Published:** 2015-12-13

**Authors:** Huiping Zhu, Yangdong Wang, Hengfu Yin, Ming Gao, Qiyan Zhang, Yicun Chen

**Affiliations:** ^1^State Key Laboratory of Tree Genetics and Breeding, Chinese Academy of Forestry, Beijing 100091, China; ^2^Institute of Subtropical Forestry, Chinese Academy of Forestry, Hangzhou 311400, China

## Abstract

Leucine-rich repeat receptor-like kinases (LRR-RLKs) make up the largest group of RLKs in plants and play important roles in many key biological processes such as pathogen response and signal transduction. To date, most studies on LRR-RLKs have been conducted on model plants. Here, we identified 236 and 230* LRR-RLKs* in two industrial oil-producing trees:* Vernicia fordii* and* Vernicia montana*, respectively. Sequence alignment analyses showed that the homology of the RLK domain (23.81%) was greater than that of the LRR domain (9.51%) among the* Vf/VmLRR-RLKs*. The conserved motif of the LRR domain in* Vf/VmLRR-RLKs* matched well the known plant LRR consensus sequence but differed at the third last amino acid (W or L). Phylogenetic analysis revealed that* Vf/VmLRR-RLKs* were grouped into 16 subclades. We characterized the expression profiles of* Vf/VmLRR-RLKs* in various tissue types including root, leaf, petal, and kernel. Further investigation revealed that* Vf/VmLRR-RLK* orthologous genes mainly showed similar expression patterns in response to tree wilt disease, except 4 pairs of* Vf/VmLRR-RLKs* that showed opposite expression trends. These results represent an extensive evaluation of* LRR-RLKs* in two industrial oil trees and will be useful for further functional studies on these proteins.

## 1. Introduction

Plants and animals respond to changes in their environment via cell surface receptors, which allow them to sense both external and internal signals and adapt accordingly. Receptor-like protein kinases (RLKs) are one of the most important groups of cell surface receptors. These proteins have special structural features that make them particularly suitable for cell-to-cell signaling. Since the first RLK was identified in maize [1], many studies have functionally characterized RLKs from various plants, including rice, poplar, soybean, and potato, and have shown that the RLKs make up a superfamily in plants. A typical RLK usually includes three distinct parts: an extracellular N-terminal domain, a single transmembrane (TM) domain, and a C-terminal intracellular kinase domain. RLKs can be classified according to their extracellular N-terminal domain. The RLKs with a leucine-rich-repeat (LRR) N-terminal domain, the LRR-RLKs, are the largest group of proteins in the RLK superfamily.

LRR-RLK proteins in various organisms contain a consensus motif of 20–30 amino acid residues [2] that is tandemly repeated to build the domain [3]. The distinguishing feature of an LRR motif is an 11-amino acid consensus sequence, LxxLxLxxNxL, where x is any amino acid [4]. This domain can bind to ligands or participate in protein-protein interactions [4].

The protein kinase (PK) domain of LRR-RLKs usually consists of approximately 250–300 amino acid residues [3] and has a cytoplasmic PK domain [5]. LRR-RLKs can be classified into three types depending on their cytoplasmic PK domain: (1) protein Ser/Thr kinases, (2) protein tyrosine kinases, and (3) protein histidine kinases [6]. The Ser/Thr kinases have been well studied in plants. The Ser/Thr domain transduces signals downstream via autophosphorylation and then phosphorylates specific substrates [7].

Previous studies have shown that the LRR-RLK family has 216 members in* Arabidopsis thaliana* [7], 234 members in* Solanum lycopersicum* [8], 379 members in* Populus trichocarpa* [9], and 309 members in* Oryza sativa* [3]. This extreme expansion in plant genomes reflects their functional significance [10]. Members of the LRR-RLK family have been shown to play critical and diverse roles in physiological processes such as secondary wall formation [11], embryogenesis [12], meristematic growth [13], maintaining vascular tissue polarity [14], germination speed [15], regulation of organ shape [16], pollen self-incompatibility [17], negative regulator-programmed cell death [18], signaling pathways [19], abscisic acid (ABA) early signaling [20], brassinosteroid signaling [21], hormone regulation [22], pathogen defense [23], tolerance to oxidative stress [15], and tolerance to salt and heat stress [10].

To date, most* LRR-RLK* genes have been isolated from model plants and herbs, rather than woody oil plants. Tung oil tree (*Vernicia fordii*) and wood oil tree (*Vernicia montana*) are important industrial oil plants belonging to the Euphorbiaceae family. The oil extracted from tung seeds is an excellent drying oil that is renewable, safe, and environmentally friendly. This oil is widely used in industrial products such as paints, plasticizers, resins, medicine, synthetic rubber, and printing ink [24], and as a raw material for biodiesel production [25]. China produces approximately 70–80% of the tung oil on the global market. However, tung trees are susceptible to* Fusarium* wilt disease. Interestingly, the two different species of* Vernicia* show different degrees of resistance to this disease;* V. fordii*, which is the main oil-producing species, is susceptible to the disease, while wood oil tree (*V. montana*) is resistant. A previous study showed that many LRR-RLKs are defense-related [10]; therefore, studies on the LRR-RLKs of these two* Vernicia* species may help to clarify why one species is more resistant than the other.

In this study, we identified the* LRR-RLKs* in two* Vernicia* species and conducted multiple sequence alignments, phylogenetic analyses, and conserved motif analyses of the* Vf*LRR-RLK and* Vm*LRR-RLK gene families. We selected several* LRR-RLK* genes for gene expression analyses in various tissues of* V. fordii* and* V. montana*. Finally, we investigated the changes in expression of 22* Vm/fLRR-RLK* genes during infection with* Fusarium oxysporum*. These results will be useful for further studies on the functions of LRR-RLKs in woody oil trees.

## 2. Materials and Methods

### 2.1. Plant Materials

Samples of* V. fordii* and* V. montana* were collected from Fuyang Urban Forest Park, Hangzhou city, Zhejiang Province, China, and then separated into roots, stems, leaves, flower buds, ovaries, and kernels. No specific permits were required to collect the samples from the park. Three replicates were collected for all samples. The samples were immediately frozen in liquid nitrogen and stored at −80°C until use.

### 2.2. Total RNA Isolation and cDNA Synthesis

Total RNA was extracted separately from each sample using an RN38-EASY Spin Plus Plant RNA kit (Aidlab Biotech, Beijing, China) following the manufacturer's instructions. The concentration of purified RNA was determined by agarose gel electrophoresis and spectrophotometry (NanoDrop 5000, Thermo Scientific, Waltham, MA, USA). Only RNA samples with a 260/280 wavelength ratio between 2.0 and 2.2 and a 260/230 wavelength ratio greater than 1.8 were used for cDNA synthesis. The cDNA was synthesized using Superscript III RT (Invitrogen, Carlsbad, CA, USA) following the manufacturer's instructions. All cDNA synthesis reactions were performed at the same time so that the efficiency of reverse transcription was approximately equal among the samples. The cDNAs were diluted 1 : 10 with nuclease-free water for RT-PCR and amplification.

### 2.3. Screening for* LRR-RLK* Genes in* V. fordii* and* V. montana*

The members of the LRR-RLK superfamily in the two* Vernicia* species were first identified from transcriptome data using look-up function of computer and using “LRR” as the key word; then we sought the selected genes one by one according to their descriptions of annotations. All hit genes were considered to be the purpose genes. Then, the corresponding ORF and amino acid sequences were identified. For all of the obtained protein sequences, the presence of characteristic domains (LRR, TM, and RLK domains) was confirmed using the Conserved Domain Database of NCBI (http://www.ncbi.nlm.nih.gov/Structure/cdd/wrpsb.cgi). Controversial sequences were used as search queries at PBLAST (http://blast.ncbi.nlm.nih.gov/Blast.cgi?PROGRAM=blastp&PAGE_TYPE=BlastSearch&LINK_LOC=blasthom). Sequences not belonging to the LRR-RLK family were rejected. The Simple Modular Architecture Research Tool (SMART) (http://smart.embl-heidelberg.de/) [26] was used as a secondary method to confirm the presence of the domain(s). All of the obtained sequences were submitted to the NCBI. Arabidopsis LRR-RLK amino acid sequences with known functions were downloaded from the NCBI database.

### 2.4. Sequence Alignment and Construction of Phylogenetic Trees

Multiple sequence alignments of amino acid sequences of RLK domains and full-length amino acid sequences of* Vernicia* LRR-RLKs with complete domains were performed using ClustalX v.1.83 [27] using the default settings. DNAMAN v.5.5.2 was used as a secondary method for aligning sequences and rechecking results.

There were some studies of LRR-RLKs in many plants, such as* A. thaliana*,* S. lycopersicum*,* P. trichocarpa*, and* O. sativa*; however, much more information about the functional classification was reported in* A. thaliana*. To compare the evolutionary relationships of LRR-RLKs between* Vernicia* species and* A. thaliana* and roughly predict the functions of LRR-RLKs in* Vernicia* species, multiple sequence alignments were performed for* Vm*LRR-RLK,* Vf*LRR-RLK, and 35* At*LRR-RLKs with known functions using the amino acid sequences of the RLK domains.

The phylogenetic trees were constructed with the neighbor-joining method using MEGA 5.1 software [28] with position correction, pairwise deletion, and 1000 bootstrap replicates indicated at each node.

### 2.5. Motif Recognition of LRR-RLKs in* Vernicia* Species

The conserved motifs of LRR-RLK protein sequences in two* Vernicia* species were identified using the motif-based sequence analysis tool, Multiple Expectation Maximization for Motif Elicitation (MEME) Suite version 4.10.0 (http://meme.nbcr.net/meme90/tools/meme) [29], with the following parameters: any number of repetitions of a motif, maximum number of motifs = 25.

### 2.6. Inoculation of* V. fordii* and* V. montana* with* Fusarium* Pathogen

The tung wilt disease pathogen* F. oxysporum* was cultivated in potato dextrose broth (PDB, 1/4 strength) on a shaker at 180 rpm (28°C) for 4 days to reach a fungal titer of 10^6^ spores/mL. Roots of 2-month-old seedlings were dug from the soil, rinsed with water, then soaked in 75% alcohol for 1 min, 0.5% sodium hypochlorite for 3 min, 90% alcohol for 30 s, and then rinsed three times in sterile water. The roots were wounded with a sterile knife, dipped in 100 mL spore liquid, and then replanted in soil. After this infection process, the plants were cultivated in an artificial climate chamber (8 h light/16 h dark) at 26°C with 95% relative humidity. The plants were observed regularly and the disease incidence was recorded [30]. Roots of plants were collected, and the stage of infection was determined according to the symptoms of the seedlings.

### 2.7. Real-Time Quantitative PCR (RT-qPCR)

The primers used for RT-qPCR were designed using Primer Premier 5.0 with the following criteria: product size between 100 and 250 bp; melting temperature around 60°C; 40–60% GC content; and primer length of 18–21 bp. Primers specific for* ACT7* (Actin7a) [31] were used to standardize the cDNA. Subsequently,* LRR-RLK* gene-specific primers (Table 1) were used to amplify the corresponding genes. The qRT-PCRs were carried out using an SYBR Premix Ex Taq Kit (TaKaRa, Tokyo, Japan) according to the manufacturer's protocol. Each PCR mixture (20 *μ*L) consisted of 2 *μ*L 4-fold diluted 1st-strand cDNA, 10 *μ*L 2x SYBR Premix Ex Taq, 0.4 *μ*L 10 *μ*M forward and reverse primers, 0.4 *μ*L 50x ROX reference dye, and 6.8 *μ*L DEPC-treated water. Reactions were performed on an ABI 7300 Real-Time quantitative instrument (Applied Biosystems, Foster City, CA, USA). The cycling parameters were as follows: 95°C for 30 s, 40 cycles of 95°C for 5 s, and 60°C for 31 s. A melting curve analysis was performed after the PCR cycling to verify the specificity of the amplification.

## 3. Results and Discussion

### 3.1. Identification of* VfLRR-RLKs* and* VmLRR-RLKs* in* V. fordii* and* V. montana*

A total of 286 and 260 candidate genes in the LRR-RLK superfamily were obtained based on annotations of RNA-seq data. Then, 236 and 230 sequences in* V. fordii* and* V. montana* with at least one characteristic domain were positively identified as members of the LRR-RLK superfamily. All of the 466 sequences were submitted to the NCBI by our laboratory, and the accession numbers were listed in Table 2.

The LRR-RLK family proteins contained at least one full or partial characteristic domain (LRR, TM, and/or RLK domains). According to the structural characteristics of the LRR-RLKs in the two* Vernicia* species, the proteins were classified into seven groups (Table 3): group 1 with an LRR domain; group 2 with a TM domain; group 3 with an RLK domain; group 4 with LRR and TM domains; group 5 with LRR and RLK domains; group 6 with TM and RLK domains; and group 7 with LRR, TM, and RLK domains. As shown in Table 3, groups 1, 3, and 5 had the most members and group 4 had the fewest members (three in* V. fordii* and five in* V. montana*). The number of members in each group was similar between* V. fordii* and* V. montana*, possibly because of the close genetic relationship between these two species.

Approximately 223, 234, 309, and 379* LRR-RLK* genes were identified in the* A. thaliana*,* O. sativa*,* S. Lycopersicum*, and* P. trichocarpa* genomes, respectively [7]. Our results showed that there were fewer* LRR-RLK* members in* Vernicia* species than in* O. sativa* and* P. trichocarpa*. This may be related to interspecific differences or functional differentiation of* LRR-RLKs*. The genome sequences also provided information on the different ratios of* Vernicia* homologues to* LRR-RLK* genes in other species.

### 3.2. Alignment and Evolutionary Analysis of VfLRR-RLKs and VmLRR-RLKs

**Figure 1:**
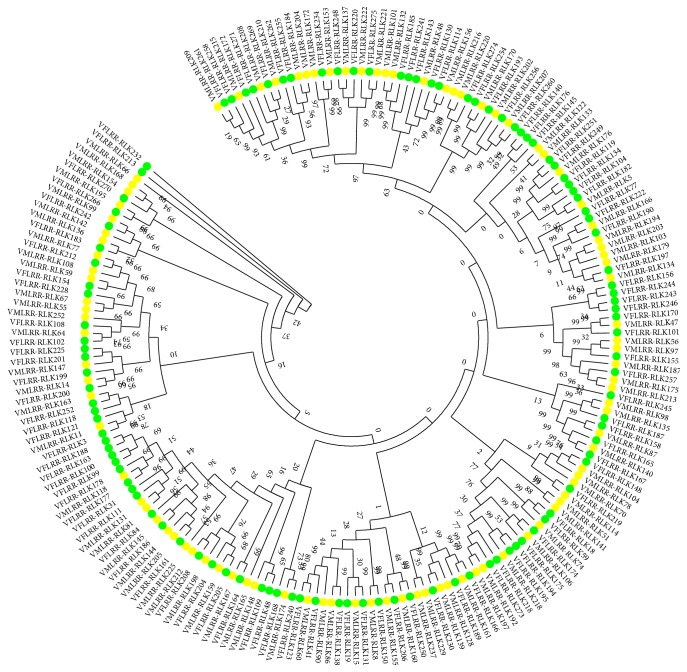
Phylogenetic tree based on the RLK sequences of* Vf/Vm*LRR-RLKs. The phylogenetic tree was constructed by MEGA package v5.1 using neighbor-joining method. The numbers at each branch point represent the bootstrap scores (1,000 replicates). The VfLRR-RLKs were signed by circle filled with green, and the VmLRR-RLKs were signed by circle filled with yellow. Amino acid sequences of RLK domain used were listed in supplementary Data Set 1.

Because of the large differences in length and complexity among the sequences, it was difficult to conduct alignments for all of the LRR-RLKs identified in these two* Vernicia* species. Therefore, we conducted alignments for the protein groups with the most members. First, we analyzed proteins with the LRR domain, since these were the most abundant. When the LRR domain was selected for the alignment the consistency was approximately 3.90%. Therefore, we selected different sequences, trimmed both ends of the sequences, and tried the alignment again. The consistency reached 9.51%, which was still too low to build a phylogenetic tree. The low consistency of LRR domains suggested a high degree of sequence complexity and diversity between VfLRR-RLKs and VmLRR-RLKs. Therefore, we selected the RLK domain amino acids sequence containing 53–394 amino acids from 201 LRR-RLK genes in* Vernicia* species for alignment. The consistency among these sequences was 23.81% (Supplementary Figure  1 in Supplementary Material available online at http://dx.doi.org/10.1155/2015/823427). To analyze the evolutionary relationships of the LRR-RLK superfamily in these two* Vernicia* species, an unrooted NJ phylogenetic tree was constructed based on the multiple sequence alignments of 106 VfLRR-RLKs and 95 VmLRR-RLKs containing the RLK domain (Figure 1). There is no standard classification method for LRR-RLKs. In previous studies, these proteins were usually classified into different subfamilies according to clades in the phylogenetic tree. Therefore, we grouped the VfLRR-RLKs and VmLRR-RLKs into 16 subclades according to the phylogenetic tree (Figure 1, subclades 1–16). Subclades 14, 15, and 16 had only one member, indicating that these subfamilies had few members or their members were too different to group into the same subclade in the tree.

To confirm the reliability of the phylogenetic tree, a phylogenetic tree was constructed for each of the two species, using the sequences of 106 VfLRR-RLK (Supplementary Figure  2) and 95 VmLRR-RLK RLK (Supplementary Figure  3) proteins. The evolutionary relationships were generally consistent among the three trees. The genes showing close relationships in the tree constructed for a single species also showed close relationships in the tree combining both species. Some VfLRR-RLK or VmLRR-RLK proteins classified into the same clade in the tree for each single species grouped into different clades in the tree combining the two species, possibly because of the more elaborate classification in the larger tree.

**Figure 2:**
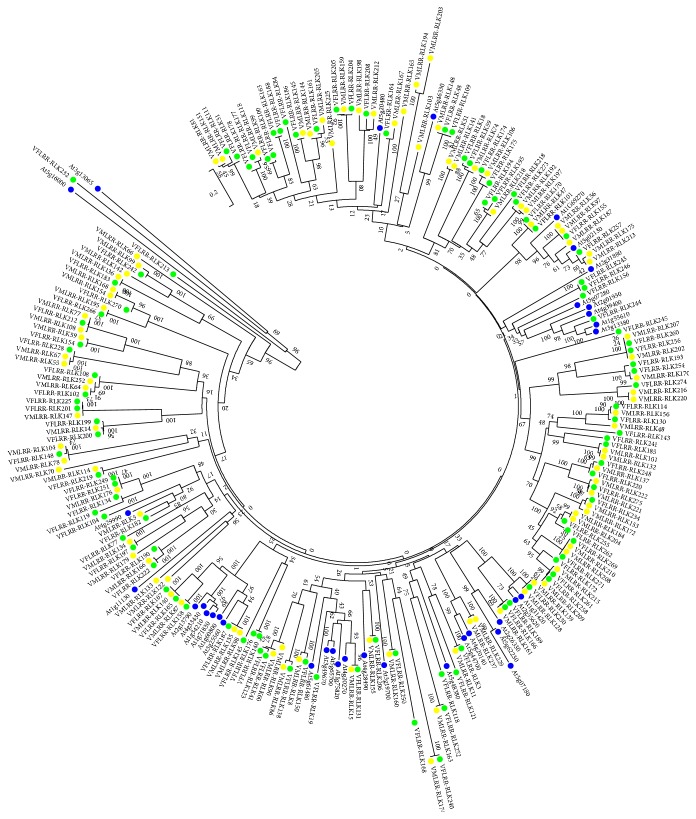
Phylogenetic tree based on the RLK sequences of LRR-RLK gene family both in* V. fordii*,* V. montana*, and* A. thaliana*. The phylogenetic tree was constructed by MEGA package v5.1 using neighbor-joining method. The numbers at each branch point represent the bootstrap scores (1,000 replicates). The LRR-RLKs of* V. Fordii* were signed by circle filled with green, the LRR-RLKs of* V. montana* were signed by circle filled with yellow, and the LRR-RLKs of* Arabidopsis thaliana* were signed by circle filled with blue. The accession number and the amino acid sequences of the* A. thaliana* used were listed in supplementary Data Set 1.

To predict the function of LRR-RLKs in* Vernicia* species, 35 Arabidopsis LRR-RLKs with known functions (Table 4) were compared with VfLRR-RLKs and VmLRR-RLKs (Figure 2). Almost every LRR-RLK subfamily in* A. thaliana* corresponded to an LRR-RLK subclade in* Vernicia* species. The members of subclade 7 in* V. fordii* and* V. montana* (Figures 1 and 2) grouped together with members of subfamily II in* A. thaliana*, suggesting that they may share the same function. These proteins may participate in brassinosteroid signaling, pathogen responses, cell death, and male sporogenesis. Similarly, members of subclade 6 in* Vernicia* species may be related to the plant brassinosteroid receptor, vascular differentiation, abscisic acid signaling, embryonic pattern formation, another development, cell death, and innate immunity. Subclade 10 members in* V. fordii* and* V. montana* may play a role in the pathogen response. Interestingly, the members of subclade 9 in* Vernicia* species corresponded to two different subclades in* A. thaliana*: AtLRR-RLKXIII and AtLRR-RLKXI. This may reflect functional differentiation of LRR-RLKs in* A. thaliana*. Based on the roles of AtLRR-RLKXIII and AtLRR-RLKXI proteins in Arabidopsis, the members of subclade 9 in* Vernicia* species may be involved in meristem differentiation, epidermal surface formation during embryogenesis, floral organ abscission, determination of seed size, cell wall biosynthesis, organ growth, and stomatal patterning and differentiation.

### 3.3. Motif Analysis of Vf/VmLRR-RLKs

**Figure 3:**
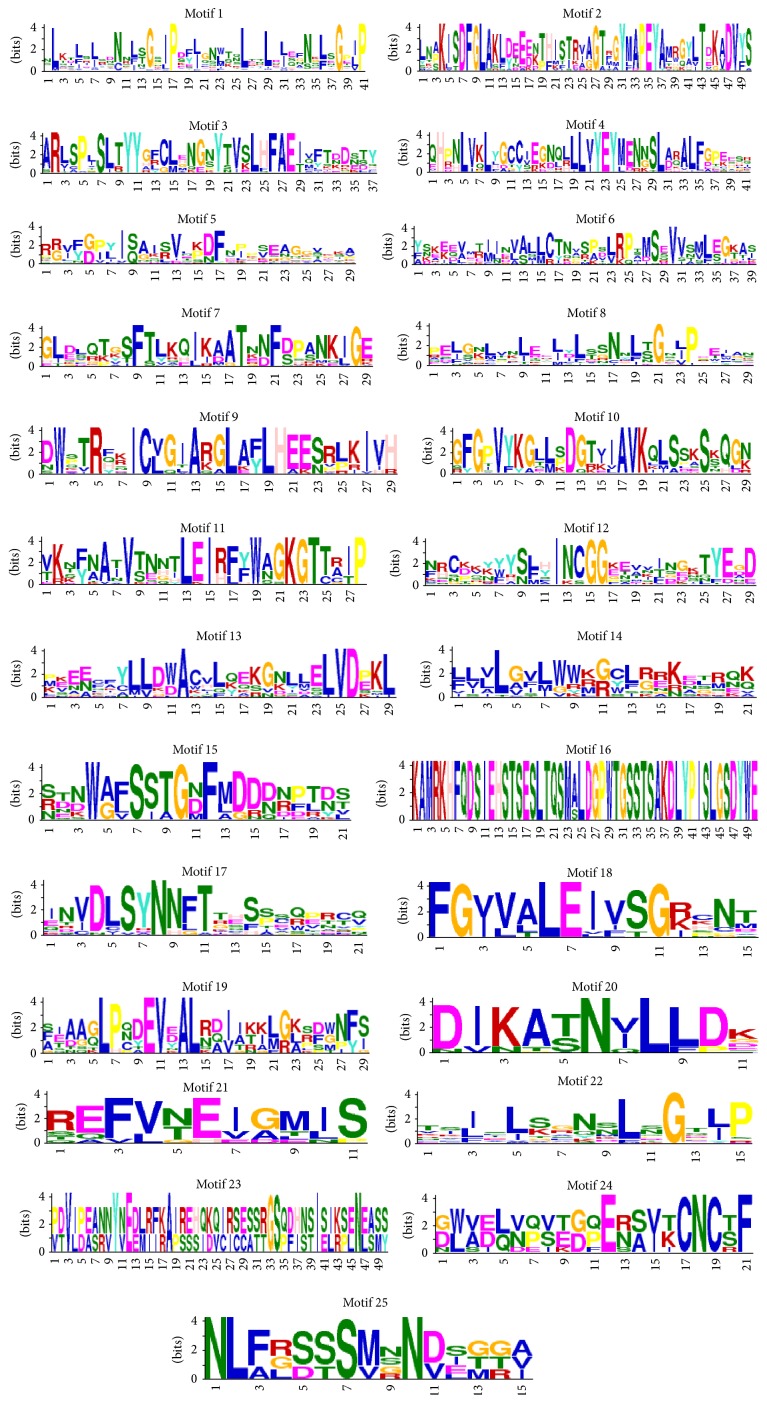
Display of conserved motifs of* Vf/VmLRR-RLK* gene family. The conserved motifs were searched in 20 Vf/VmLRR-RLKs which contained full characteristic domains (the amino acid sequences were listed in supplementary Data Set 2) by Multiple Expectation Maximization for Motif Elicitation (MEME) Suite version 4.10.0. Overall height in each stack indicates the sequence conservation at that position; height of each residue letter indicates relative frequency of the corresponding residue.

To further reveal the diversification and potential functions of LRR-RLKs in* Vernicia*, we selected 20 Vf/VmLRR-RLKs (Table 5) with full characteristic domain and investigated their conserved motifs using MEME version 4.10.0. A total of 25 conserved motifs were identified and numbered 1–25 (Figure 3).

**Figure 4:**
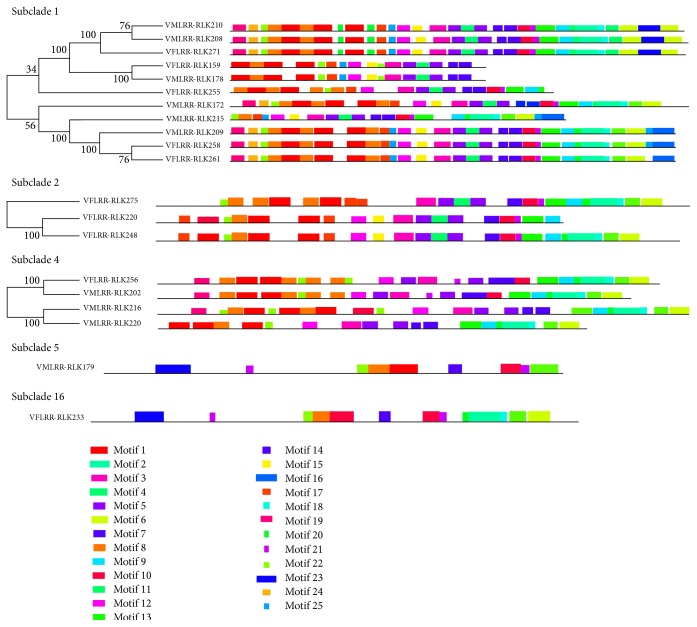
Conserve motifs of different subclades of LRR-RLKs in* Vernicia* species. The conserve motifs of each LRR-RLK gene were searched by Multiple Expectation Maximization for Motif Elicitation (MEME) Suite version 4.10.0. Different colors and different lengths boxes represent different motifs.

Among the 20 Vf/VmLRR-RLKs, there were six different motifs at the N-terminal and six at the C-terminal. The six motifs at the N-terminal were Motifs 19, 1, 8, 22, 17, and 23. Ten of the 20 LRR-RLKs (50%) had Motif 19 at the N-terminal, and most of these LRR-RLKs were in subclades 1 and 4 (Figure 4). The other five motifs were present in one to three of the 20 LRR-RLKs. Interestingly, Motif 17 was present at the N-terminal of two LRR-RLKs, both of which were in subclade 2. This may indicate that Motif 17 is specific to subclade 2. There were too few members of subfamilies 16 and 5 to make accurate predictions about their motif structure.

The six motifs at the C-terminal were Motifs 6, 20, 16, 4, 9, and 7. Motif 6 was present in 11 of the 20 LRR-RLKs (55%), and in almost every subclade. All members of subclade 4 had Motif 6 at their C-terminal. Subclade 5 had only one member, which had Motif 4 at its C-terminal. Motif 16 was present in four of the 20 LRR-RLKs, all of which were in subclade 1. The other C-terminal motifs were detected in only one or two of the 20 LRR-RLKs.

The motifs of different domains were detected according to their sequences and sites. The most obvious motif was that of the LRR domain, characterized by repeated “L” residues. This motif was present in Motifs 22, 8, and 1. Among them, Motif 1 was the most representative of the basic LRR structural skeleton, with the sequence LxxLxLxxNxLxGxIPxxLxxW/Lxx. This sequence matched well the plant LRR consensus sequence (LxxLxLxxNxLxGxIPxxLxxLxx) but differed at the third last amino acid (W or L). Motifs 12, 15, 3, and 5 corresponded to the TM domain, and Motifs 10, 4, 9, 20, 2, 13, and 6 corresponded to the RLK domain. Among all of the motifs, the most conserved structure of LRR-RLKs in* Vernicia* species was the RLK domain containing Motifs 4, 9, 20, 2, 13, and 6.

### 3.4. Expression of* VfLRR-RLKs* and* VmLRR-RLKs* in Response to* Fusarium* Infection

*Fusarium* wilt disease of tung oil tree is a devastating fungal soil-borne disease that severely affects tree growth.* V. fordii*, which is the main oil-producing species, is susceptible to this disease, while* V. montana* (wood oil tree) is resistant. To investigate the responses of* Vm*/*Vf*LRR-RLKs to the* Fusarium* wilt pathogen, we collected roots from plants before infection (stage 0), at an early stage of* F. oxysporum* infection (stage 1), and at a late stage of* F. oxysporum* infection (stage 2). We randomly selected 22* Vm*/*Vf*LRR-RLK orthologous genes and monitored their transcript levels by RT-PCR. The 22 orthologous genes were* VfLRR-RLK2/VmLRR-RLK18*,* VfLRR-RLK6/VmLRR-RLK17*,* VfLRR-RLK7/VmLRR-RLK30*,* VfLRR-RLK9/VmLRR-RLK29*,* VfLRR-RLK11/VmLRR-RLK111*,* VfLRR-RLK13/VmLRR-RLK241*,* VfLRR-RLK159/VmLRR-RLK178*,* VfLRR-RLK172/VmLRR-RLK164*,* VfLRR-RLK256/VmLRR-RLK206*,* VfLRR-RLK260/VmLRR-RLK202*, and* VfLRR-RLK271/VmLRR-RLK210*. All genes were amplified reliably.

**Figure 5:**
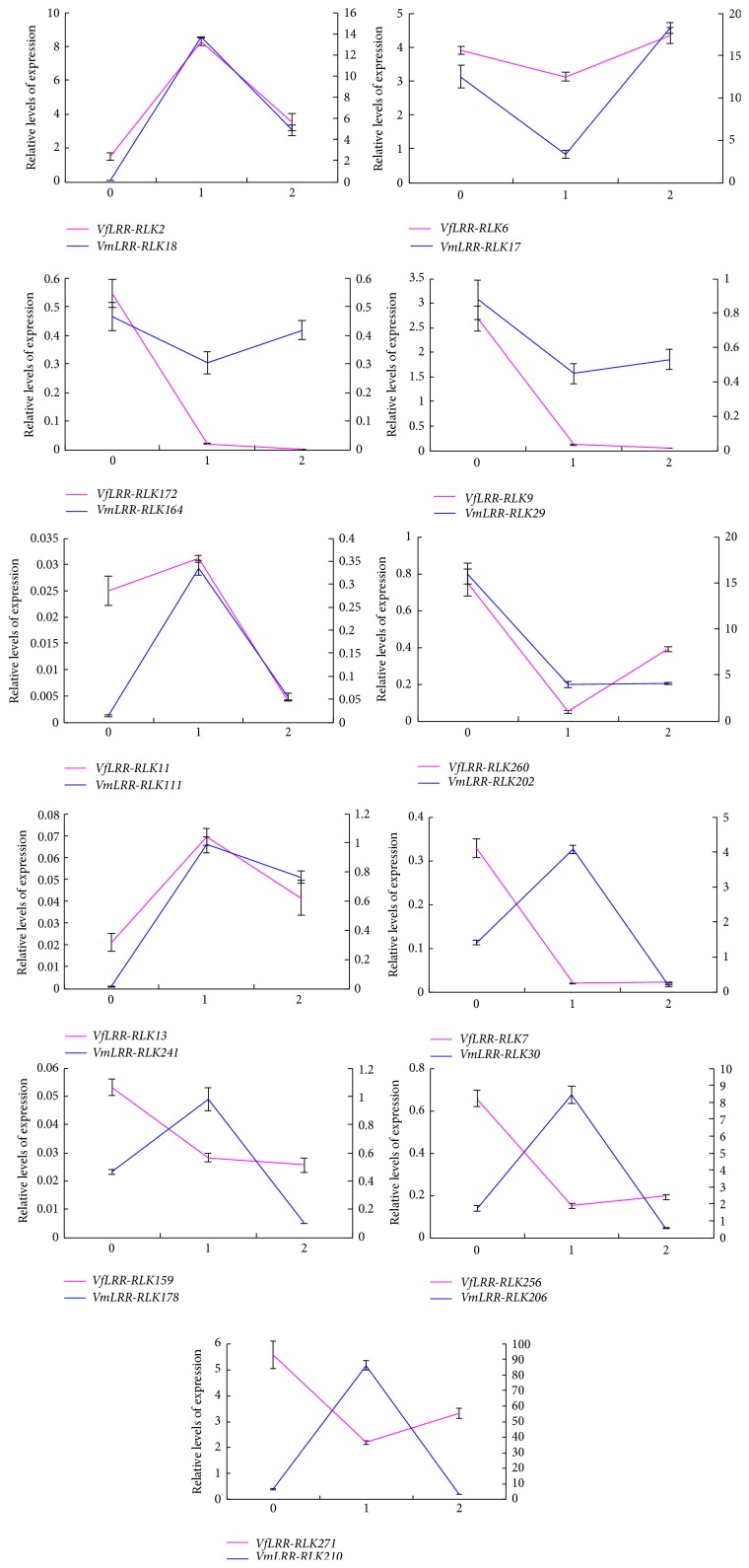
Expression analysis of 22* Vm/Vf LRR-RLK* genes in roots of* Vernicia* during infection with* Fusarium*. Vertical axis represents gene transcript levels. Primary axis represents transcript levels of* Vf LRR-RLKs*; secondary axis represents transcript levels of* VmLRR-RLKs*. Standard errors are shown (*n* = 3 biological samples). Each sample was analyzed by real-time PCR in triplicate. 0, before infection; 1, early stage of infection; 2, late stage of infection.

The qRT-PCR results showed that although there were some differences in transcript levels between pairs of orthologous genes, most of them showed similar transcription profiles in response to* Fusarium* wilt disease in both* V. fordii* and* V. montana* during the infection period (Figure 5). This result suggests that many* Vf/VmLRR-RLKs* have similar functions during pathogen infection. Four pairs of orthologous genes (*VfLRR-RLK7/VmLRR-RLK30*,* VfLRR-RLK159/VmLRR-RLK178*,* VfLRR-RLK256/VmLRR-RLK206*, and* VfLRR-RLK271/VmLRR-RLK210*) showed opposite expression patterns between* V. montana* and* V. fordii*. In* V. montana*, the transcript levels of* VmLRR-RLK30*,* 178*,* 206*, and* 210* increased at the early stage of infection, whereas those of the corresponding orthologous genes in* V. fordii*,* VfLRR-RLK7*,* 159*,* 256*, and* 271*, decreased. This finding suggests that these four* VmLRR-RLK* genes participate in resistance to* F. oxysporum* in* V. montana*.

### 3.5. Transcription Patterns of* VfLRR-RLKs* and* VmLRR-RLRs* in Various Tissues

**Figure 6:**
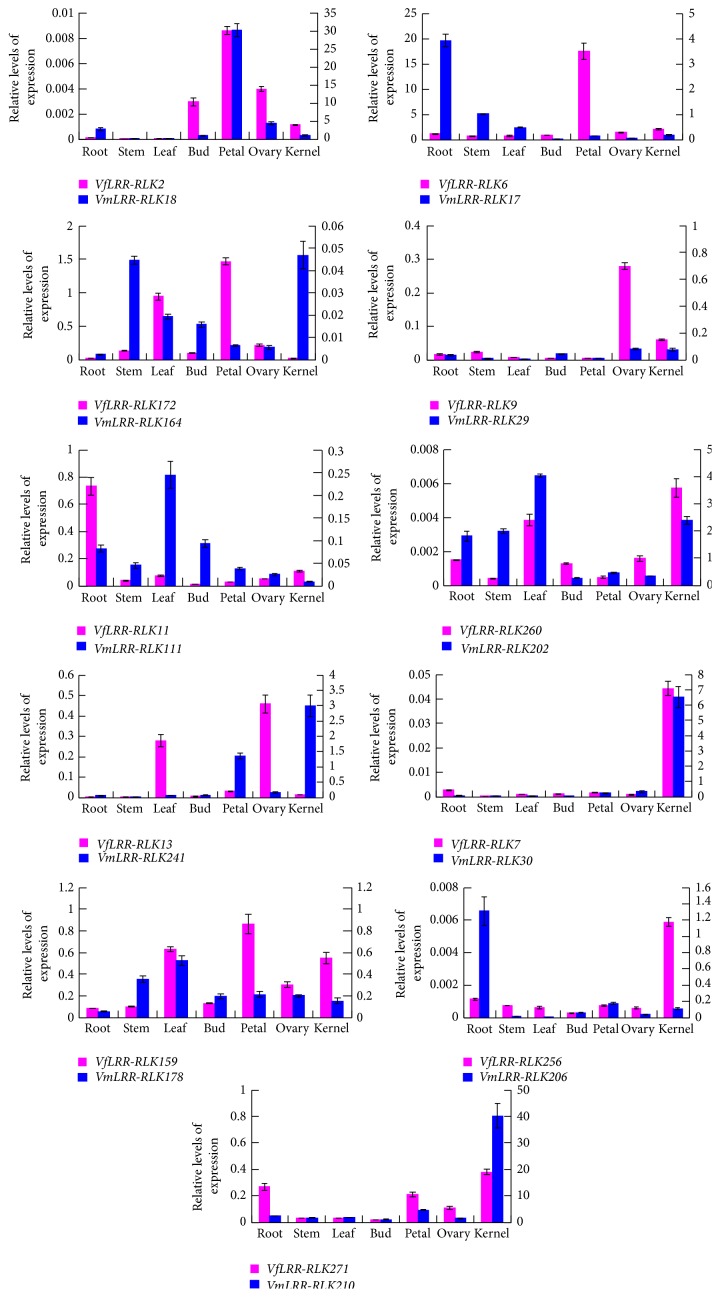
Transcript levels of 22* Vm/Vf LRR-RLK* genes in various tissues. Column height shows gene transcript levels. Primary axis represents transcript levels of* Vf LRR-RLKs*; secondary axis represents transcript levels of* VmLRR-RLKs*. Standard errors are shown (*n* = 3 biological samples). Each sample was analyzed by real-time PCR in triplicate.

To investigate the tissue specificity of* VfLRR-RLKs* and* VmLRR-RLRs* expression and further analyze genes related to* Fusarium* wilt disease, we analyzed the transcript levels of the 22 genes described above in seven tissues of* V. fordii* and* V. montana* by qRT-PCR (Figure 6). Among them,* VfLRR-RLK260* and* VfLRR-RLK159* showed similar expression patterns in all seven tissues of* V. fordii*. Both showed higher transcript levels in leaves and kernels and lower transcript levels in roots, stems, buds, and ovaries. However, compared with* VfLRR-RLK260*,* VfLRR-RLK159* was more strongly expressed in petals, suggesting that it may have a special function in floral development.* VfLRR-RLK2* was expressed in roots, stems, and leaves and strongly expressed in petals, but not in vascular tissues.* VfLRR-RLK172* was expressed most strongly in petals, followed by leaves, but expressed at low levels in the other tissues.* VfLRR-RLK13* showed the highest transcript level in ovaries, followed by leaves.* VfLRR-RLK271* showed similar expression patterns in all tissues. The other five genes showed tissue-specific expression patterns.* VfLRR-RLK6* was specifically expressed in petals,* VfLRR-RLK9* in ovaries, and* VfLRR-RLK11* in roots. Both* VfLRR-RLK7* and* VfLRR-RLK256* were specifically expressed in kernels. Together, these results suggest that* VfLRR-RLKs* play various roles in the development of tung tree.

Compared with* VfLRR-RLK*s, most* VmLRR-RLK*s showed higher transcript levels in the seven tissues analyzed. Six* VmLRR-RLK*s (*VmLRR-RLK18*,* VmLRR-RLK29*,* VmLRR-RLK202*,* VmLRR-RLK30*,* VmLRR-RLK178*, and* VmLRR-*RLK210) showed the same expression patterns as their orthologous genes in* V. fordii*. This may indicate that they share the same function in* V. fordii* and* V. montana.* The other five* VmLRR-RLK*s showed different expression patterns in* V. montana*.* VmLRR-RLK111* was mainly expressed in leaves and had similar transcript levels in other tissues.* VmLRR-RLK164* and* VmLRR-RLK241* showed peak expression in kernels, but* VmLRR-RLK164* was also expressed at high levels in the stems. Both* VmLRR-RLK17* and* VmLRR-RLK206* showed the highest transcript levels in roots and lower levels in other tissues. The different expression patterns in* V. montana* may reflect functional differentiation during evolution.

Among the seven pairs of orthologous genes showing similar trends in gene expression in* V. montana* and* V. fordii* in response to* Fusarium* infection, three pairs also showed similar expression patterns in the tissues (*VfLRR-RLK2/VmLRR-RLK18*,* VfLRR-RLK9/VmLRR-RLK29*, and* VfLRR-RLK260/VmLRR-RLK202*). The other four pairs showed different expression patterns in the seven tissues analyzed. Of the four pairs of orthologous genes showing opposite responses to* Fusarium* infection in* V. montana* and* V. fordii* (Figure 5), three pairs showed similar expression patterns in the tissues, and one pair (*VfLRR-RLK256* and* VmLRR-RLK206*) showed different expression patterns in the tissues (Figure 6). There were high transcript levels of* VfLRR-RLK256* in kernels and* VmLRR-RLK206* in the roots. Given that the* Fusarium* pathogen invades via the roots of tung tree, these results suggest that* VmLRR-RLK206* may play a role in resistance to* Fusarium* wilt disease.

## 4. Conclusion

This is the first extensive evaluation of the LRR-RLK superfamily in tung oil tree and wood tung tree. Phylogenetic analyses, conserved motif analyses, and expression analyses of* VfLRR-RLKs* and* VmLRR-RLKs* in different tissues and in response to* Fusarium* infection were conducted. Characterization of* LRR-RLK* genes in a ligneous oil plant will improve our understanding of the evolutionary processes and functions of this gene superfamily. The results of this study provide important information for further research on the diversity and functions of the* LRR-RLK* gene family in tung tree.

## Supplementary Material

Supplementary Fig.1 Multiple alignment of RLK domain for LRR-RLK family from V. fordii and V. montana. The multiple alignment was performed using Clustal X v.1.83 with the default settings, and the figure was output by DNAMAN v.5.5.2. Residues highlighted in different color represent different amino acids identity, respectively. Amino acid sequences of RLK domain used were list in supplementary Data Set 1.
Supplementary Fig.2 Phylogenetic tree based on the RLK sequences of VfLRR-RLKs. The phylogenetic tree was constructed by MEGA package v5.1 using neighbor-joining method. The numbers at each branch point represent the bootstrap scores (1,000 replicates). Amino acid sequences of RLK domain used were list in supplementary Data Set 1.
Supplementary Fig.3 Phylogenetic tree based on the RLK sequences of VmLRR-RLKs. The phylogenetic tree was constructed by MEGA package v5.1 using neighbor-joining method. The numbers at each branch point represent the bootstrap scores (1,000 replicates). Amino acid sequences of RLK domain used were list in supplementary Data Set 1.
Supplementary Data Set 1 Amino acid sequences of RLK domain from V. fordii , V. Montana and A. Thaliana. All of the amino acid sequences used to conduct multiple sequence alignments and construction of phylogenetic trees were listed in this data set.
Supplementary Data Set 2 Amino acid sequences for motif analysis. All of the amino acid sequences used to perform motif analysis were listed in this data set.

## Figures and Tables

**Table 1:** Sequences of primers specific for *VfLRR-RLK* and *VmLRR-RLK* genes amplification used qRT-PCR.

Primer names	Sequences (5′-3′)	*T*_*m*_	Amplicons length (bp)
*VfLRR-RLK2*	GGCTCAATCCCAGAATCAT/CCAACAGAAACGAAACATC	51.9	166
*VfLRR-RLK6*	TTTAGACTTGCTGCCTGAC/TAACACCACCAGTGACCTG	54.1	121
*VfLRR-RLK7*	TCAGGCAGTGAGGTAGAAG/GAAGCACGAGAAAGATTAA	48.9	182
*VfLRR-RLK9*	ATTTCGCCACCATAGAGTC/ATTTCATCCTGCGTAAGTG	51.9	167
*VfLRR-RLK11*	AGGAGAACAGGAAGCCCACT/GAAGGTCCATAAATGTATCA	53.3	198
*VfLRR-RLK13*	GACCTGAAAACTGGAAATG/TATGTAACCAATGGAGCCT	49.9	147
*VfLRR-RLK159*	TTAGGATAAGCGACAACAA/GCTACTCAGATTGGGAAAA	49.75	197
*VfLRR-RLK172*	GTCCAATTTCGGTCGGTTGC/AGGAATGGTGTTCGGGTTT	55.2	132
*VfLRR-RLK256*	CTTGAGCGGTGGGCGTATC/ACCCTGGAATGACGAAGGTATG	58.6	181
*VfLRR-RLK260*	CTTGAGCGGTGGGCGTATC/ACCCTGGAATGACGAAGGTATG	58.6	181
*VfLRR-RLK271*	CAACAGTCTCAACGGAAGC/AATGGATGATGGAATGGGT	53.0	106
*VmLRR-RLK18*	TTGCCTCATGGAAATCCGACA/GGCCTGTAAGATTGGTTAAT	53.45	156
*VmLRR-RLK17*	GGACCAGCAGTTGTGAGT/CTTTCTGTTGGGTGGAGA	53.75	168
*VmLRR-RLK30*	ATGTCAGGCAGTGAGGTAG/GAAGCACGAGAAAGATTAA	51.95	135
*VmLRR-RLK29*	CGCCACCATAGAGTCATAG/CACTTACGCAGGATGAAAT	53.0	163
*VmLRR-RLK111*	TTCTTCTGGAGATCCCATTT/GTAAACCATCCTTTGCCTC	52.15	151
*VmLRR-RLK241*	TGACCTGAAACCTGGAAAT/GCCACCCATACCATACTCT	53	172
*VmLRR-RLK178*	TTAGGATAAGCGACAACAA/GCTACTCAGATTGGGAAAA	49.75	197
*VmLRR-RLK164*	CTATGGAGGGTCCTATTC/TTAAGCCAGTGATTGAGC	51.45	159
*VmLRR-RLK206*	TCGCAAATCGCCTTTATTC/ATGGCTATGCTAGGGTCAA	51.9	118
*VmLRR-RLK202*	CTTGAGCGGTGGGCGTATC/ACCCTGGAATGACGAAGGTATG	58.6	181
*VmLRR-RLK210*	TCATAGGCCCAGAACACTC/TCCTGGTGCTTATGTGAGT	54.1	231
*ACT*	CGATGAAGCACAGTCCAAAAG/GTTGAGAGGAGCCTCAGTG	58.85	170

**Table 2:** GenBank accession numbers of *VfLRR-RLK* and *VmLRR-RLK* genes.

Vernicia fordii		Vernicia montana	
Gene ID	GenBank accession number	Gene ID	GenBank accession number
*VfLRR-RLK1-VfLRR-RLK14*	c805427-KT805440	*VmLRR-RLK1-VmLRR-RLK9*	KT805663-KT805671
*VfLRR-RLK18-VfLRR-RLK19*	KT805441-KT805442	*VmLRR-RLK11-VmLRR-RLK18*	KT805672-KT805679
*VfLRR-RLK22-VfLRR-RLK24*	KT805443-KT805445	*VmLRR-RLK20-VmLRR-RLK32*	KT805680-KT805692
*VfLRR-RLK26-VfLRR-RLK28*	KT805446-KT805448	*VmLRR-RLK34*	KT805693
*VfLRR-RLK30-VfLRR-RLK46*	KT805449-KT805465	*VmLRR-RLK36-VmLRR-RLK39*	KT805694-KT805697
*VfLRR-RLK48*	KT805466	*VmLRR-RLK41-VmLRR-RLK67*	KT805698-KT805724
*VfLRR-RLK50-VfLRR-RLK53*	KT805467-KT805470	*VmLRR-RLK69-VmLRR-RLK83*	KT805725-KT805739
*VfLRR-RLK55-VfLRR-RLK57*	KT805471-KT805473	*VmLRR-RLK86-VmLRR-RLK90*	KT805740-KT805744
*VfLRR-RLK59-VfLRR-RLK60*	KT805474-KT805475	*VmLRR-RLK92*	KT805745
*VfLRR-RLK63-VfLRR-RLK65*	KT805476-KT805478	*VmLRR-RLK94-VmLRR-RLK108*	KT805746-KT805760
*VfLRR-RLK68-VfLRR-RLK70*	KT805479-KT805481	*VmLRR-RLK110-VmLRR-RLK115*	KT805761-KT805766
*VfLRR-RLK72*	KT805482	*VmLRR-RLK117-VmLRR-RLK118*	KT805767-KT805768
*VfLRR-RLK74-VfLRR-RLK90*	KT805483-KT805499	*VmLRR-RLK120*	KT805769
*VfLRR-RLK92-VfLRR-RLK93*	KT805500-KT805501	*VmLRR-RLK122-VmLRR-RLK123*	KT805770-KT805771
*VfLRR-RLK95-VfLRR-RLK97*	KT805502-KT805504	*VmLRR-RLK125-VmLRR-RLK129*	KT805772-KT805776
*VfLRR-RLK99-VfLRR-RLK104*	KT805505-KT805510	*VmLRR-RLK131-VmLRR-RLK142*	KT805777-KT805788
*VfLRR-RLK106-VfLRR-RLK124*	KT805511-KT805529	*VmLRR-RLK144-VmLRR-RLK151*	KT805789-KT805796
*VfLRR-RLK126*	KT805530	*VmLRR-RLK153-VmLRR-RLK157*	KT805797-KT805801
*VfLRR-RLK128*	KT805531	*VmLRR-RLK159-VmLRR-RLK187*	KT805802-KT805830
*VfLRR-RLK130-VfLRR-RLK140*	KT805532-KT805542	*VmLRR-RLK190*	KT805831
*VfLRR-RLK143*	KT805543	*VmLRR-RLK192-VmLRR-RLK195*	KT805832-KT805835
*VfLRR-RLK145-VfLRR-RLK146*	KT805544-KT805545	*VmLRR-RLK197-VmLRR-RLK199*	KT805836-KT805838
*VfLRR-RLK148*	KT805546	*VmLRR-RLK202-VmLRR-RLK206*	KT805839-KT805843
*VfLRR-RLK150-VfLRR-RLK156*	KT805547-KT805553	*VmLRR-RLK208-VmLRR-RLK222*	KT805844-KT805858
*VfLRR-RLK158-VfLRR-RLK159*	KT805554-KT805555	*VmLRR-RLK225-VmLRR-RLK245*	KT805859-KT805879
*VfLRR-RLK161-VfLRR-RLK170*	KT805556-KT805565	*VmLRR-RLK247-VmLRR-RLK257*	KT805880-KT805890
*VfLRR-RLK172-VfLRR-RLK190*	KT805566-KT805584	*VmLRR-RLK259-VmLRR-RLK260*	KT805891-KT805892
*VfLRR-RLK193-VfLRR-RLK197*	KT805585-KT805589		
*VfLRR-RLK199-VfLRR-RLK202*	KT805590-KT805593		
*VfLRR-RLK204-VfLRR-RLK208*	KT805594-KT805598		
*VfLRR-RLK210*	KT805599		
*VfLRR-RLK212-VfLRR-RLK214*	KT805600-KT805602		
*VfLRR-RLK216-VfLRR-RLK220*	KT805603-KT805607		
*VfLRR-RLK222-VfLRR-RLK223*	KT805608-KT805609		
*VfLRR-RLK225*	KT805610		
*VfLRR-RLK227-VfLRR-RLK230*	KT805611-KT805614		
*VfLRR-RLK232-VfLRR-RLK237*	KT805615-KT805620		
*VfLRR-RLK239-VfLRR-RLK246*	KT805621-KT805628		
*VfLRR-RLK248-VfLRR-RLK258*	KT805629-KT805639		
*VfLRR-RLK260-VfLRR-RLK264*	KT805640-KT805644		
*VfLRR-RLK266-VfLRR-RLK276*	KT805645-KT805655		
*VfLRR-RLK279-VfLRR-RLK281*	KT805656-KT805658		
*VfLRR-RLK283-VfLRR-RLK286*	KT805659-KT805662		

**Table 3:** The number of LRR-RLK genes containing different conserved domains in *V. fordii *and *V. montana*.

Species	Number of Total	Number of LRR	Number of TM	Number of RLK	Number of LRR-TM	Number of LRR-RLK	Number of TM-RLK	Number of LRR-TM-RLK
*V. fordii*	236	75	12	73	3	52	11	10
*V. montana*	229	75	6	68	5	55	10	10

**Table 4:** Subclassification of LRR-RLK genes in *A. thaliana*, *V. fordii,* and *V. Montana*.

Subgroup in *A. thaliana*	Gene name (accession number in GenBank)	Functions	Reference	Subgroup in *V. fordii*	Subgroup in *V. montana*
LRR I	LRRPK (At4g29990)	Light signal transduction	[1]	*Vf*LRR-RLK182 (subclade 5)	*Vm*LRR-RLK5 (subclade 5)

LRR II	BAK1/AtSERK3 (At4g33430); BKK1/AtSERK4 (At2g13790); AtSERK1 (At1g71830); AtSERK2 (At1g34210); NIK1 (At5g16000); NIK2 (At3g25560); NIK3 (At1g60800)	Antiviral defense response; BR signaling; cell death; male sporogenesis; and pathogen response	[32–34]	*Vf*LRR-RLK187 (subclade 7)	*Vm*LRR-RLK135 (subclade 7); *Vm*LRR-RLK98 (subclade 7)

LRR V	SRF4 (At3g13065); Scrambled/SRF9/SUB/Strubbelig (At1g11130)	Cell morphogenesis; leaf size control; organ development; positional signaling; and root epidermis patterning	[13, 21, 35, 36]	*Vf*LRR-RLK232 (subclade 16)	*Vm*LRR-RLK122 (subclade 5) *Vm*LRR-RLK133 (subclade 5)

LRR X	BRI1 (At4g39400); BRL1 (At1g55610); BRL2/VH1 (At2g01950); BRL3 (At3g13380); RPK1/TOAD1 (At1g69270); RPK2/TOAD2 (At3g02130); EMS1/EXS (At5g07280); BIR1 (At5g48380)	Abscisic acid signaling; anther development; brassinosteroid receptor; cell death and innate immunity; embryonic pattern formation; and vascular different	[20, 22, 37–40]	*Vf*LRR-RLK155 (subclade 6); *Vf*LRR-RLK257 (subclade 6); *Vf*LRR-RLK244 (subclade 6); *Vf*LRR-RLK3 (subclade 9); *Vf*LRR-RLK121 (subclade 9); *Vf*LRR-RLK118 (subclade 11); *Vf*LRR-RLK252 (subclade 11)	*Vm*LRR-RLK56 (subclade 6) *Vm*LRR-RLK97 (subclade 6) *Vm*LRR-RLK187 (subclade 6) *Vm*LRR-RLK175 (subclade 6) *Vm*LRR-RLK213 (subclade 6) *Vm*LRR-RLK11 (subclade 9) *Vm*LRR-RLK163 (subclade 11)

LRR XI	GSO1 (At4g20140); GSO2 (At5g44700); CLV1 (At1g75820); BAM1 (At5g65700); BAM2 (At3g49670); BAM3 (At4g20270); SOBIR1 (At2g31880); HAESA (At4g28490); IKU2 (At3g19700); PXY/TDRv (At5g61480)	Anther development; cell death and innate immunity; epidermal surface Embryogenesis; formation during floral organ abscission; meristem differentiation; and seed size	[40–44]	*Vf*LRR-RLK243 (subclade 6); * Vf*LRR-RLK246 (subclade 6); * Vf*LRR-RLK156 (subclade 6); * Vf*LRR-RLK206 (subclade 9); * Vf*LRR-RLK19 (subclade 9)	*Vm*LRR-RLK229 (subclade 9) *Vm*LRR-RLK237 (subclade 9) *Vm*LRR-RLK155 (subclade 9) *Vm*LRR-RLK15 (subclade 9)

LRR XII	FLS2 (At5g46330); EFR (At5g20480)	Pathogen response	[45]	*Vf*LRR-RLK164 (subclade 10) *Vf*LRR-RLK48 (subclade 10) *Vf*LRR-RLK109 (subclade 10)	*Vm*LRR-RLK167 (subclade 10) *Vm*LRR-RLK148 (subclade 10)

LRR XIII	FEI1 (At1g31420); FEI2 (At2g35620); ERECTA (At2g26330); ERL1 (At5g62230); ERL2 (At5g07180)	Cell wall biosynthesis; organ growth; and stomatal patterning and differentiation	[46–48]	*Vf*LRR-RLK230 (subclade 9)	*Vm*LRR-RLK139 (subclade 9) *Vm*LRR-RLK128 (subclade 9)

**Table 5:** Basic information of some *Vf*LRR-RLK and *Vm*LRR-RLK family genes.

Gene name	Amino acids length	L content (%)	PI	Molecular mass (KD)	LRR-Domain	TM-Domain	RLK-Domain
Basic information of some *Vf*LRR-RLK family genes							
*Vf*LRR-RLK159	567	11.88	9.06	62669.6	25–204	269–453	534–564
*Vf*LRR-RLK220	782	11.75	8.29	86377.1	187–333	384–569	656–781
*Vf*LRR-RLK233	836	10.16	7.01	93442.0	389–446	5–312	566–780
*Vf*LRR-RLK248	1002	11.93	6.71	110540.3	187–333	384–569	655–922
*Vf*LRR-RLK255	719	13.04	8.41	79622.9	6–220	297–481	564–708
*Vf*LRR-RLK256	991	12.10	6.91	111107.1	155–311	445–624	701–967
*Vf*LRR-RLK258	986	10.48	6.15	108664.5	248–319	380–561	644–913
*Vf*LRR-RLK261	984	10.62	6.05	108287.2	248–319	380–561	644–913
*Vf*LRR-RLK271	1010	11.04	5.93	112529.3	159–302	378–553	634–901
*Vf*LRR-RLK275	1036	11.08	5.03	113890.5	292–351	428–612	700–963

Basic information of some *Vm*LRR-RLK family genes							
*Vm*LRR-RLK172	1028	11.97	8.31	112996.4	116–342	413–600	683–950
*Vm*LRR-RLK178	567	11.87	9.19	62741.8	269–453	25–205	518–558
*Vm*LRR-RLK179	659	11.08	7.02	74326.8	389–446	5–312	566–651
*Vm*LRR-RLK202	935	12.04	6.95	105218.5	155–311	390–569	646–912
*Vm*LRR-RLK208	1018	10.94	5.42	113540.2	280–302	378–561	642–909
*Vm*LRR-RLK209	986	10.69	6.19	108573.5	259–319	380–561	644–913
*Vm*LRR-RLK210	1010	10.83	5.50	112606.3	280–302	378–553	634–901
*Vm*LRR-RLK215	742	9.60	7.49	82247.5	2–60	99–280	363–669
*Vm*LRR-RLK216	1061	13.01	8.29	117955.4	147–276	514–695	772–1050
*Vm*LRR-RLK220	847	12.16	8.51	94738.0	19–221	293–474	551–829
