# Supplementary material for: Genome-Wide Identification and Characterization of the LRR-RLK Gene Family in Two Vernicia Species
Source: Int J Genomics. 2015 Dec 13;2015:823427. doi: 10.1155/2015/823427 (PMC4691485; doi:10.1155/2015/823427)
Supplement: Supplementary file 1 — Supplementary Fig.1 Multiple alignment of RLK domain for LRR-RLK family from V. fordii and V. montana. The multiple alignment was performed using Clustal X v.1.83 with the default settings, and the figure was output by DNAMAN v.5.5.2. Residues highlighted in different color represent different amino acids identity, respectively. Amino acid sequences of RLK domain used were list in supplementary Data Set 1. Supplementary Fig.2 Phylogenetic tree based on the RLK sequences of VfLRR-RLKs. The phylogenetic tree was constructed by MEGA package v5.1 using neighbor-joining method. The numbers at each branch point represent the bootstrap scores (1,000 replicates). Amino acid sequences of RLK domain used were list in supplementary Data Set 1. Supplementary Fig.3 Phylogenetic tree based on the RLK sequences of VmLRR-RLKs. The phylogenetic tree was constructed by MEGA package v5.1 using neighbor-joining method. The numbers at each branch point represent the bootstrap scores (1,000 replicates). Amino acid sequences of RLK domain used were list in supplementary Data Set 1. Supplementary Data Set 1 Amino acid sequences of RLK domain from V. fordii , V. Montana and A. Thaliana. All of the amino acid sequences used to conduct multiple sequence alignments and construction of phylogenetic trees were listed in this data set. Supplementary Data Set 2 Amino acid sequences for motif analysis. All of the amino acid sequences used to perform motif analysis were listed in this data set. [file 823427.f1.pdf]

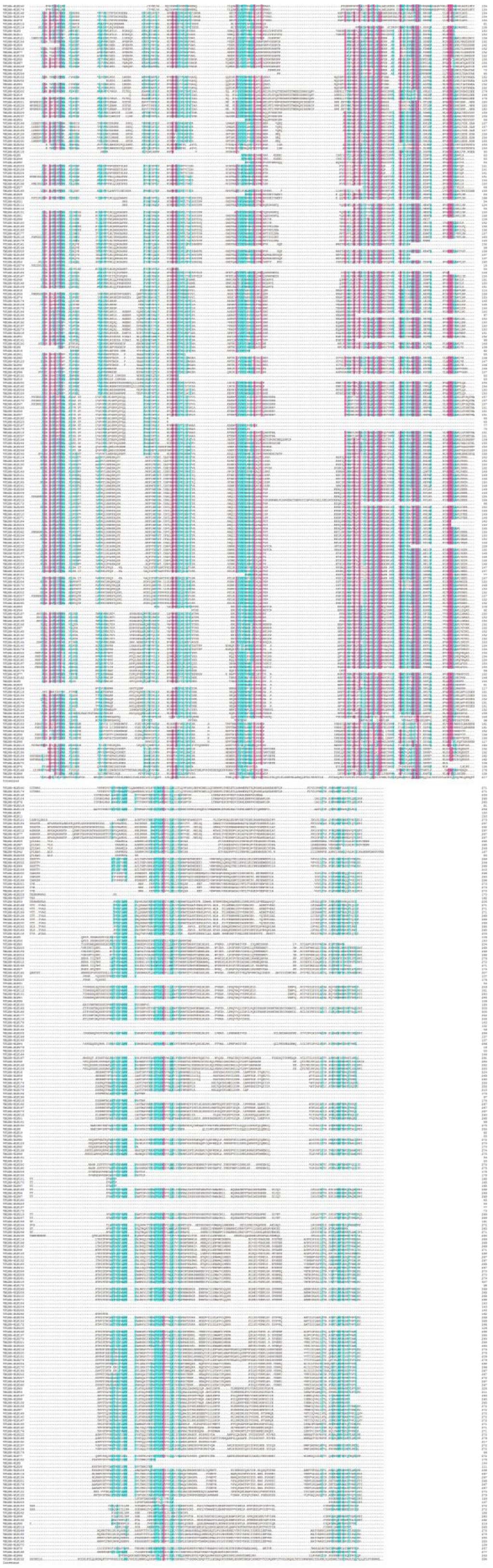

Supplementary Fig.1 Multiple alignment of RLK domain for *LRR-RLK* family from *V. fordii* and *V. montana*

The multiple alignment was performed using Clustal X v.1.83 with the default settings, and the figure was output by DNAMAN v.5.5.2. Residues highlighted in different color represent different amino acids identity, respectively. Amino acid sequences of RLK domain used were list in supplementary Data Set 1.

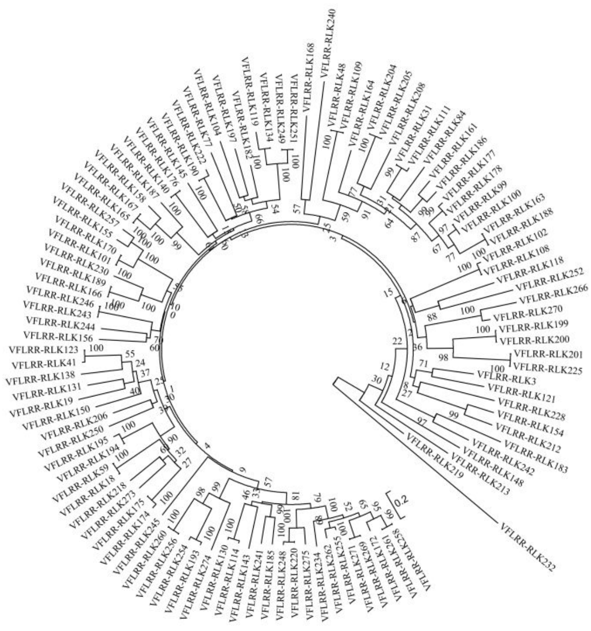

Supplementary Fig.2 Phylogenetic tree based on the RLK sequences of VfLRR-RLKs

The phylogenetic tree was constructed by MEGA package v5.1 using neighbor-joining method. The numbers at each branch point represent the bootstrap scores (1,000 replicates). Amino acid sequences of RLK domain used were list in supplementary Data Set 1.

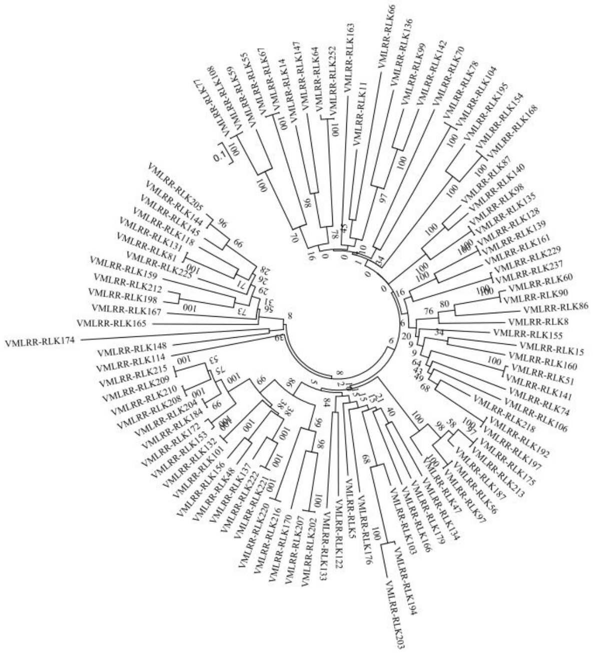

Supplementary Fig.3 Phylogenetic tree based on the RLK sequences of VmLRR-RLKs

The phylogenetic tree was constructed by MEGA package v5.1 using neighbor-joining method. The numbers at each branch point represent the bootstrap scores (1,000 replicates). Amino acid sequences of RLK domain used were list in supplementary Data Set 1.

supplementary Data Set 1 Amino acid sequences of RLK domain from *V. fordii* , *V. Montana* and *A. Thaliana*

Amino acid sequences of RLK domain from *V. fordii*
>VFLRR-RLK3
IGGGSIGTVYRTNFEGGISIAVKKLETLGRIRSQDEFEQEIGRLGNLRHPNLVSFQGYYWSSSMQ
LILSEFVPNGNLYDNLHGLDYPGTSTGVGNSELYWSRRFQIALGTARALSYLHHDCRPPILHLNI
KSTNILLDENYEAKLSDYGL
>VFLRR-RLK18
KTFKSEMYSLTEIRHRNIVKMHGFSYLNGSLYFVYEFARGGSLGELLQEKKKAKILNWDLRLK
VIKGVANALSYLHHDCTPAVVHRDISGSNILLDAEFEAKISDFGTARVLRKSESNWTVPVGSYG
YIAPELASTIKVTEKCDVHSFGVVALEVILGKHPHELLLCLQSGGYDMLFSYILDKRLAPPTGPI
VQELVLAVTLALLCIRENPKSRPTMHQVSSEL
>VFLRR-RLK19
MPNGELIAVKKLWKTKRDKESVDSFAAEIQILGHIRHRNIVRLLGYCSNKSVKLLLYNYIPN
>VFLRR-RLK31
SKSFIAECKALRNIRHRNLVKILTYCSSIDFKGNDFKALVFDFMENGSLDTWLH
>VFLRR-RLK41
LGRGRSGIVYMVTLPSGSTVAVKRFKTMDKFSAAAFSSEIATLARIRHRNIVRLL
>VFLRR-RLK48
IIGASSLSTVYKGQLEDGQMVAVKKLNLQQFPAESDKSFYREVKTLSQLRHKNLVKVLGYAWE
SRKLKALVLEYMNNGSLESIIHDAHVDQSKWTLSQRIDVWISVASGLEYLHSGYDFPIVHCDLK
PSNILLDSSWVAHVSDFGTARILGVHLQDGSSLSSSSAFQGTIGYLAPEFAYMRKVTTKVDVFSF
GIIIMEFLTKRRPTGLTEEHGIPISLSQLFEKALGNGINGLLQVLDPVIAMNASKDEQTLTELFKL
ALCCTNPNPDDRPNMNEVLSSLNKLK
>VFLRR-RLK59
FNEKHSIGRGGQGSVYKAILPSGDIFAVKRLHPSEDNVSSEEYQMKTFKSEMYSLTEIRHRNIVK
MHGFSYLNGSLYFVYEFARGGSLGELLQEKKKAKILNWDLRLKVIKGVANALSYLHHDCTPA
VVHRDISGSNILLDAEFEAKISDFGTARVLRKSESNWTVPVGSYGYIAPELASTIKVTEKCDVYS
FGVVALEVILGKHPHELLLCLQSGGYDMLFSYILDKRLAPPTGPIVQELVLAVTLALLCIRENPK
SRPTMHQVSSEL
>VFLRR-RLK77
VGSGAFGNVYKGIFEVEGTLAIKRAHADSFQSVEEFRNEVKLLSKVKHSNLVSLVGFCEENGG
GKRAKVLVYEYVPNGSLLEYIIGRRGRSLTWRQRVNIAIGAAKGIAHLHDGVTPSIIHRDIKPSN
ILVGEDFEAKVSDFGLVKMGPIGDQSHVSSQIKGTPGYLDPAYCSSFHLSPFSDVYSFGVILLQL
VSARPAVDSSRSQSNYHIIEWARPSLERGNVAEILDANLLTEPCNMEIMLKMGQLGLRCVVQNP
KDRPTMTQVWQELEE
>VFLRR-RLK84
NLIGFGSYGSVYKGLLHQSQSFIAVKVFNLQLRGASKSFIHECKALRNIRHRNLLKIFSVCSSAD
HQGNDFQALIYEFIPRGSLDKWLYPEVTKDDYGFSTLNLIQRLNIVIDVACALEYLHCDCQPPIV
HSDIKPSNVLLDDDMVAHVGDFGLARVLSTVSSDAQDDQSNSIIKGSIGYIAPEYGMGEGASIQ
GDVYSFGILIMEIFTTRKPTDGMFQGDLNLHNFTVMALPERVMDIVDPQLLFEDNEAGRMQLC
LISVLRVGLSCSVEIPRDRMEIRNVVREL

>VFLRR-RLK99
IGIGSFGSVYKGILDGGETLIAIKVFNLMRRGAFKSFLAECEALRNIRHRNLVKVLTACSSVDYQ
GNDFKALVYEFMVNGSLEEWLHPLVETRNLNILQRLNIAIDVACALDYLHHRCETQIVHCDLK
PSNVLLDKELT
>VFLRR-RLK100
IGIGSFGSVYKGILDGGETLIAIKVFNLMRRGAFKSFLAECEALRNIRHRNLVKVLTACSSVDYQ
GNDFKALVYEFMVNGSLEEWLHPLVETRNLNILQRLNIAIDVACALDYLHHRCETQIVHCDLK
PSNVLLDKELTGCVGDFGLARILPEAIYNNTNESSSIGVRGTIGYAPPEYGIGNEVS
>VFLRR-RLK101
FSIRNLIGTGGFGSTYKAELAPGYLVAVKRLSLGRFQGIQQFDAEIRTLGRIRHKNLVTLIGYYV
GEAEMFLIYNYLSGGNLETFIHERSSKNVQWSVIYKIAFDIAQALAYLHYSCVPRILHRDIKPSN
ILLDEELNAYLSDFGLAKLLEVSQTHATTDVAGTF
>VFLRR-RLK102
LSRTRYGLVFKACYNDGMVLSIRRLPDGLMDENMFRKEAEFLSKVKHRNLTVLRGYYAGQQQ
DLRLVVYDYMPNGNLATLLQEASHQDGHVLNWPMRHLIALGIARGLAFLHTSNMVHGDVKP
QNVLFDADFEAHLSDFGLDRLTIATPAEASSSTTVGTLGYVSPEAILTGEVSKESDVYSFGIVLLE
LLTGKRPVMFNEDEDIVKWVKKQLQRGQITELLEPGLLELDPESSEWEEFLLGVKVGLLCTAP
DPLDRPTMSDIVFMLEG
>VFLRR-RLK104
MVIFCRMKRRGATTQSNKEGNLVSLIGYCDDRHNKALIYEYMVYGNLREHLSETSGSILNWDE
RLQIAVDAAHGLEYLHNGCKPPIIHRDLKTSNILINDKLQAKIADFGLSRAFKNESGSHITTHPA
GTYGYLDPEAHATANFSQKSDVYSFGIILLELITGQPAIKRDMTGKIILIQKWVTSIIERGDIQSLV
DPRLQGQFDTNSAWKVVETALTCVLNSAIQRPDMSHVLAELKE
>VFLRR-RLK108
LSRTRYGLVFKACYNDGMVLSIRRLPDGLMDENMFRKEAEFLSKVKHRNLTVLRGYYAGQQQ
DLRLVVYDYMPNGNLATLLQEASHQDGHVLNWPMRHLIALGIARGLAFLHTSNMVHGDVKP
QNVLFDADFEAHLSDFGLDRLTIATPAEASSSTTVGTLGYVSPEAILTGEVSKESDVYSFGIVLLE
LLTGKRPVMFNEDEDIVKWVKKHYRE
>VFLRR-RLK109
IIGASSLSTVYKGQLEDGQMVAVKKLNLQQFPAESDKSFYREVKTLSQLRHKNLVKVLGYAWE
SRKLKALVLEYMNNGSLESIIHDAHVDQSKWTLSQRIDVWISVASGLEYLHSGYDFPIVHCDLK
PSNILLDSSWVAHVSDFGTARILGVHLQDGSSLSSSSAFQGTIGYLAPEFAYMRKVTTKVDVFSF
GIIIMEFLTKRRPTGLTEEHGIPISLSQLFEKALGNGINGLLQVLDPVIAMNASKDEQTLTELFKL
ALCCTNPNPDDRPNMNEVLSSLNKL
>VFLRR-RLK111
SKSFIAECKALRNIRHRNLVKILTYCSSIDFKGNDFKALVFDFMENGSLDTWLHQEGKGNTQVQ
NLNFLQRLHIAIDVSFALLYLHDDCEAPVIHCDLKPSNILLDNEMTAHVGDFGLSKLLSKTINNS
SLGQTSSIGIKGTIGYMAPEYGIGSEATTSGDVYSFGIILLEIFTGKKPTDEMFINGLNLHNFVKA
KIPGQVMQVVDPKLELGDNNVQKCIVSILEIGLACSAEQVGERMNMRDVTRKLN
>VFLRR-RLK114
KVGEGGFGIVYKGVLKDGTVAAIKVLSPESRQGVREFLTEIKLIADVEHDNLVKLYGCCVEEDH
RILVYGYLKNNSLAQTLLGGGHSSIQFNWPTRCRICIGVAQGLAFLHEEVQPHIVHRDIKASNIL
LDENLMPKISDFGLAKLFPPNLTHISTRVAGTAGYLAPEYAIRGQLTRKADIYSFGVLLLEIVCGR

SNTNRRLPPEEQYLLERVWDSHEKGELVSLVDASLNEDYDTEEACKYLKIGLLCTQDMPKLRP
TMSTVVKMLMG
>VFLRR-RLK118
MEKRSLDQWLHSKKRSTNVSGSASHLYLDWPKRFRIAVEAAQGLSYMHHDCLPPIIHRDVKSS
NILLDSSFKAKIADFGLARLLVKKGEATVSAVAGSFGYIAPEYAQTYRVNEKIDVYSFGVVLLEL
TTGKEANFGDENSCLADWAWRYMNEGNPIVHALDKEIKNPSYFDEMSIVFKLGVRCTSKLPSA
RPSMGEVLQILD
>VFLRR-RLK119
SKISMKIDGVKFFTFREMALATENFNSKTQVGQKEFLTEIRLLSRLHHRNLVSLVGYCDEEGEQ
MLVYEFMPNGTLRNWLSAKAKETLNFGMRLNTALGSAKGILYLHTEADPPVFHRDIKASNILL
DSKLTAKVADFGLSRLAPVLDDEGNLSNHVSTVVKGTPGYLDPEYFLTHKLTDKSDVYSLGIVF
LEFLTGMQPISHGKNIVREVTMAHQSGIMFSIIDSRMGSYPSECVEKFVALALGCCHDKPENRP
SMSEVVRELENI
>VFLRR-RLK121
LNKDCELGRGGFGVVYRTILRDGRSVAIKKLTVSSLIKSQDEFEKEVKRLGKIRHHNLVALEGY
YWIPSLQLLIYEFISSGSLYKHLHDGPNTNCLSWRQRFNIILGMAKGLAHLHHMNIIHYNLKST
NILIDDSGEPKVGDFGLARLLPMLDRCILSSKIQSALGYMAPEFACRTVKITEKCDVYGFGILVL
EVVTGKRPVEYMEDDVVVLCDMVKGALEDGRVDECVDGRLRGNFPADEAIPVIKLGLICASQ
VPSNRPDMEEVVNILELI
>VFLRR-RLK123
ILGRGRSGIVYMVTLPSGSTVAVKRFKTMDKFSAAAFSSEIATLARIRHRNIVRLLGWGANRKT
KLLFYDYMSNGTLGALLHEGSIVGLVEWETRFKIALGVAEGLAYLHHDCVPPILHRDVKAHNI
LLGDRYEACLADFGLARLVEDDQGSFSATPQFAGSYGYIAPEYACMLKITEKSDVYSYGVVLL
EIITGKKPVDPSFAEGQHVIQWVREQLKSKKDPVEILDPKLQGHPDTQIQEMLQALGISLLCTSN
RAEDRPTMKDVAALLREI
>VFLRR-RLK130
KVGEGGFGIVYKGVLKDGTVAAIKVLSPESRQGVREFLTEIKLIADVEHDNLVKLYGCCVEEDH
RILVYGYLKNNSLAQTLLGGNRSSIQFNWPTRCRICIGVAQGLAFLHEEVRPHIVHRDIKASNIL
LDENLMPKISDFGLAKLFPPNLTHISTRVAGTAGYLAPEYAIRGQLTRKADIYSFGVLLLEIVCGR
PNTNRRLVPEEQYLLERVWDLHEKGELVSLVDTSLNEDYNAEEACKYLKIGLLCTQDMPKLRP
TMSTVVKMLMG
>VFLRR-RLK131
IGRGGAGIVYKGIMPNGEQVAVKKLLGISKGSSHDNGLSAEIQTLGKIRHRNI
>VFLRR-RLK134
VGQGGYGKVYRGILADNTIVAIKRAEEGSLQGQKEFLTEIRLLSRLHHRNLVSLVGYCDEEGEQ
MLVYEFMPNGTLRNWLSAKAKETLNFGMRLNTALGSAKGILYLHTEADPPVFHRDIKASNILL
DSKLTAKVADFGLSRLAPVLDDEGNLPNHVSTVVKGTPGYLDPEYFLTHKLTDKSDVYSLGIVF
LEFLTGMQPISHGKNIVREVTMAHQSGIMFSIIDSRMGSYPSECVEKFVALALGCCHDKPENRP
SMSEVVRELENI
>VFLRR-RLK138
VIGTGSSGVVYKVTIPSGDSLAVKKMWSSEESGAFSSEIQTLGSIRHKNIIRLLGWGSNRNLKLL
FYDYLPNGSLSSLLHGAGKGGAEWETRYDIVLGVAHALAYLHHDCLPAILHGDVKAMNVLLG
PSYEPYLADFGLARVVSSNSSDDLAKPSQRPHLAGSYGYMAPEHASIQRITEKSDVYSFGVVLL

EVLTGRHPLDPTLPGGAPLVQWVRDHLARKKDPVDILDHKLRGRADPTMHEMLQTLAVSFLCI
STKADDRPTMKDIAAMLKEI
>VFLRR-RLK140
IIGRGGYGNVYKGELPDGSQVALKRFKNLSAAGDASFVHEVKVIASVRHVNLVALRGYCTATT
PFEGHQRIIVCDLMKNGSLHDHLFGSAKEKLSWPIRQKIALGTARGLAYLHYGAQPAIIHRDIK
ASNILLDEKFDAKVADFGLAKFTPEGVTHVSTRVAGTMGYVAPEYALYGQLTERSDVYSFGVV
LLELLSGKKALTMSDESQPASVSDWAWSLVRRGRTLDVIEDGMPELGPQEILEKYVLIAVLCSH
PQLYARPTMDQVVKMLE
>VFLRR-RLK143
IGEGGFGFVYKGRLEDGKFVAVKVLSADSKQGDREFLSEIALLSNISHQNLVKLYGGCIDGPCRI
LVYEYLEKGNLAQILLGRSKSRAKLSWKARREISIGIGEGLAYIHEEIKPHIVHRDLKASNILLD
QNFTPKVSDFGLSKLFSENLTHISTRVAGTLAYVAPEYAISGHLTRKSDVYSYGVLLLEIVSGRTA
VDFDLQLGEHYLVEKAWEMYKDDKLHQLVDPILNGNFIEDEALRFLKVGLLCVQEKCGLRPN
LSKAIKMMKG
>VFLRR-RLK145
DNKIGEGGFGSVYWGRTSKGVEIAVKRLKAMSAKAEMEFAVEVEILGRVRHKNLLGLRGFYA
GGDERLIVYDYMPNHSLITHLHGQLASDCLLDWPRRIKIVIGSAEGLAYLHHKANPHIIHRDIK
ASNVLLDKEFQAKVADFGFAKLIPDGVTHLTTRVKGTLGYLAPEYAMWGKVSESCDVYSFGIL
LLEIISARKPLKKLPGGVKLDIVHWVTPYVQKGAFDHIADPMLKGKYDLAQLKSAIMVAMRC
TDSNPENRPSMIQVVDWLKG
>VFLRR-RLK148
LGKSNFSATYKGILRDRSVVVVKCITKTSCKSDEADFLKGLKILTSFKHENLVRLRGFCCSKGR
GECFLIYDFVPNGNLLEYLDVKEGSGRVLEWSTRISIINGIAKGIGYLHGNKGNKSALFHQNISA
EKVLIDRRYNPLLSDSGLHKILADDIIFSMLKASAAMGYLAPEYTTTGRFTEKSDVYAFGMIILQ
ILSGKSNVTPLTRHAVESCKVEDFIDANLERNFPGSEAA
>VFLRR-RLK150
NIIGKGCSGMVYRVETPLKQVIAVKKLWPVKNGEIPERDWFSAEVRTLGSIRHKNIVRLLGCCN
NGKTRLLLFDYISNGSLAGLLHEKKLFLDWDARYNIILGAAHGLEYLHHDCTPPIVHRDIKAN
NILVGPQFEAFLADFGLAKLVDSAECSRVSNTVAGSYGYIAPEYGYSFRITEKSDVYSYGVVLL
EVLTGREPTDTQIPEGAHIVTWVTKELRERKRDFTTILDQQLLLRSGTQLQEMLQVLGVALLCV
NPSPEERPTMKDVTALLKEI
>VFLRR-RLK154
ILGATGSSIMYKAVLEDGTALAVRRIGENHVERFRDFETQVRVIAKLVHPNLVRIRGFYWGVDE
KLIIYDFVPNGSLSNARYRKVGSSPCHLPWEARLKIAKGVARGLSFLHDKKHVHGNLKPSNILL
GSDMEPRIGDFGLERLVMGDSSYKSSGSTRNFGSKRSTASRDSFQDFPLGPSPSPSPSSIGGLSPY
HAPESLRSIKPNPKWDVYSFGVILLELLTGKVIVVDELGQGNNGLVVDDKNRAMRMADVAIR
AEVDGKEESLLACFKLGYNCASPIPQKRPTMKEVLQVLEKI
>VFLRR-RLK155
IGSGGFGATYKAEIVPGVVVAVKRLSVGRFQGVQQFATEIRTLGRVQHPNLVKLIGYHLSETEM
FLIYNYLPGGNLETFIQERSRRVVEWSMLHKIALDIARALAYLHDECVPRVLHRDIKPSNILLDN
NFNAYLSDFGLARLLGTSETHATTDVAGTFGYVAPEYAMTCRVSDKADVYSYGVVLLELISDK
KALDPSFSSFGNGFNIVAWASMLLRQGRASEFFTAGLWDSGPHNDLIQILHLGIMCTGESLSSRP
SMKQVAQRLKRI
>VFLRR-RLK156

VGDGSFGFVYKASLSDGVIVAIKKLDTNAFQGFREFRAEMETLGKLQHPNIVRILGYCVSGAD
RVLIYEFIEKGNLDQWLHDTSPGNCNEQSSKLPLSWKTRIKVVKGLANGLAYLHQLDTPIIHRD
IKATNVLLDSDFEAHIADFGLARALDGSHSHVST
>VFLRR-RLK158
ILGQGGFGKVYKGVLQDNTKVAVKRLTDFESPGGDAAFQREVEMISVAVHKNLLRLIGFCTTPS
ERLLVYPFMMNLSVAYRLRERKPEEAVLDWATRKKVALGAARGLEYLHEHCNPKIIHRDVKA
ANVLLDEDFEAVVGDFGLAKLVDVRKTNVTTQVRGTMGHIAPEYLSTGKSSERTDVFGYGIM
LLELVTGQRAIDFSRLEEEDDVLLLDHVKKLEREKRLDAIVDRNLNKNYNILEVEMMIQVALL
CTQASPEDRPAMSDVVRMLEG
>VFLRR-RLK161
IGVGSYGSVYRGILEQVEIEVAVKVINLQQRGASNSFISECQALGSIRHRNLLKLLSVCSSMNFK
GNDFKALIYEFMANGSLEKWLHVHGQERNSTNLKLIHRLNIAIDIAFAIEYLHNGSSSTIIHGDL
KP
>VFLRR-RLK163
IGTGSFGSVYKGILDKEEGKIIAVKVLNLMHHGAFKSFVAECEALKNIRHRNLVKLITACSSTDY
QGNDFKALIYEFMVNRSLEEWLHPTFELDEATKSLDLLQRLNIAIDVASALQYLHHHCEKTIVH
CDLKPSNILLDDEMIGHVSDFGLAKILFQDTFYSSTNQSSSVGVRGTIGYTPPEYGVGSKVSTY
GDTYSYGILLLEMFTGKRPTDDMFGENMNLHNFVKTNLPKQVVQLIDPILFQEIFNSSKIKNHT
RNIEGIKNNGILECLISIFEIGIACCIDLPQDRMNIGNVVAQLSSI
>VFLRR-RLK164
IGNGGFGLVYKGCLGDGSAVAIKVIDITQTGFRKSFLAECEALRNVRHRNLVKLITSCSSVDLKN
TEFLALVYEFLGNGSLQDWIQGKRKNEDGDGLNLVERLNVAIDIASAIDYLHNDCEVPVLHCD
LKPNNILLDEDMTAKVGD
>VFLRR-RLK165
IIGQGGFGKVYKGVLPDNTKVAIKRLTDYYSPGGEAAFQREVQLISVAVHRNLLRLIGFCTTSSE
RILVYPYMRNLSVAYQLRELKPGEKGLDWPTRKRVAFGAAHGLEYLHEHCNLKIIHRDLKAAN
ILLDDNFEAVLGDFGLAKLVDTKLTHVTTQIRGTMGHIAPEYLSTGKSSEKTDVFGYGITLLELV
TGQRAIDLSRLEDEEDVLLLDHIKKLLREDRLDDIVDGNLKTYNRQEVKTIIQVALLSTQSSPED
RPTMAEVVKLLQGV
>VFLRR-RLK166
LDEEDVVGSGGFGTVYRMVMNDCGTFAVKRIDRSREGSDQVFERELEILGSIKHINLVNLRGY
CRLPMAKLLIYDYLAMGSLDDILHEHVQEQPLNWSARLSIALGSARGLAYLHHDCSPKIVHRD
IKSSNILLDENLEPHVSDFGLAKLLVDEDAHVTTVVAGTFGYLAPEYLQSGRATEKSDVYSFGV
LLLELVTGKRPTDPAFVKRGLNVVGWLNTLLRENRLDDVVDKRCKDADMETVEAILEIAARC
TDANPDDRPTMNQVLQLLE
>VFLRR-RLK167
IIGQGGFGKVYKGVLPDNTKVAIKRLTDYYSPGGEAAFQREVQLISVAVHRNLLRLIGFCTTSSE
RILVYPYMRNLSVAYQLRELKPGEKGLDWPTRKRVAFGAAHGLEYLHEHCNLKIIHRDLKAAN
ILLDDNFEAVLGDFGLAKLVDTKLTHVTTQIRGTMGHIAPEYLSTGKSSEKTDVFGYGITLLELV
TGQRAIDLSRLEDEEDVLLLDHIKKLLREDRLDDIVDGNLKTYNRQEVKTIIQVALLSTQSSPED
RPTMAEVVKLLQGV
>VFLRR-RLK168
LDIRHVVAHGTYGTVYRGTYDDQDVAVKLLDWGEDGIATTTETAALRASFQQEVAVWHKLDH
PNVTKFVGASMGTSNLKVPIKDSDGQESFPSRACCVVVDYLAGGTLKQYLIRNRRKKLAYKV

VIQLALDLSRGLSYLHSKKIVHRDVKTENMLLDTHRTLKIADFGVARVEAQNPRDMTGETGTL
GYMAPEVLDGKPYNRRCDVYSFGICLWEIYCCDMPYPDLSFADVSSAVVRQNLRPEIPRCCPSS
LAGIMRKCWDANPEKRPEMSEVVRML
>VFLRR-RLK170
FSIRNLIGTGGFGSTYKAELAPGYLVAVKRLSLGRFQGIQQFDAEIRTLGRIRHKNLVTLIGYYV
GEAEMFLIYNYLSGGNLETFIHERSSKNVQWSVIYKIAFDIAQALAYLHYSCVPRILHRDIKPSN
ILLDEELNAYLSDFGLAKLLEVSQTHATTDVAGTF
>VFLRR-RLK172
IGEGGFGPVYKGVLSDGAVIAVKQLSAKSKQGNREFVNEIGMISALQHPNLVRLYGCCIEGNQL
LLVYEYLENNSLARALFGREDQRLQLDWSTRKKI
>VFLRR-RLK174
CIGVGAHGNVYKAQLSINKIVALKKLHHSKAENLVNEVEVLTRIRHKNIVKLYGFCLHRRSMF
LIYEYMEKGSLFHALRNDTEAMELNWNIRVKIIKDIAFAVSYLHHGCNPPIVHRDISSKNILLNL
ELKAFVSDFGLARLLDPDSSNQTTMAGTYGYMAPELAYTIVMTEKCDVYSFGVVALELLMGT
HPGEFLSSSLTQNIMLNELLDSRLAPPSNDIDVQDIIFVATIAFSCLCATPKSRPTMEFLSQDFL
>VFLRR-RLK175
IGVGAHGNVYKAQLSINKIVALKKLHHSKTENLVNEVEVLTRIRHKNIVKLYGFCLHRRSMFLI
YEYMEKGSLFHALRNDTEAMELNWNIRVKIIKDIAFAVSYLHHGCNPPIVHRDISSKNILLNLEL
KAFVSDFGLARLLDPDSSNQTTMAGTYGYMAPELAYTIVMTEKCDVYSFGVVALEILMGTHP
GEFLSSSLAQNIKLNELLDSRLAP
>VFLRR-RLK176
IIGRGGYGNVYKGELPDGSQVALKRFKNLSAAGDASFVHEVKVIASVRHVNLVALRGYCTATT
PFEGHQRIIVCDLMKNGSLHDHLFGSAKEKLSWPIRQKIALGTARGLAYLHYGAQPAIIHRDIK
ASNILLDEKFDAKVADFGLAKFTPEGVTHVSTRVAGTMGYVAPEYALYGQLTERSDVYSFGVV
LLELLSGKKALTMSDESQPASVSDWAWSLVRRGRTLDVIEDGMPELGPQEILEKYVLIAVLCSH
PQLYARPTMDQVVKMLE
>VFLRR-RLK177
IGIGSFGSVYKGILDREGIVIAVKVLNLTRHGASKSFAAECETLRNIRHRNLVKVLTACSGVDYH
GNDFKALVYEFMANGSLDGWLHPTIGLDKAPKTLNVLQRLNIAIDVACALEYLHYHCVTPVIH
CDLKPSNILLDENMTGHVSDFGLVKFLSNETLDNFINQFSSIGVRGTIGYCPPEYGIGSEVSTFG
DIFSFGILLLEMFTGKRPIDDMFKESLNLHSFVKRALPDQVTQVIDPSL
>VFLRR-RLK178
IGTGSFGSVYKGILDREGIVIAVKVLNLTRHGASKSFAAECETLRNIRHRNLVKVLTACSGVDYH
GNDFKALVYDFMANGSLDGWLHPTIGLDKAPKTLNVLQRLNIAIDVACALEYLHYHCVTPVIH
CDLKPSNILLDENMN
>VFLRR-RLK182
LGKGGFGTVYHGSLDDGTQVAVKMLSPSSIQGYKEFQAEVKLLLRVHHRNLTTLVGYCNEET
NKGLIYEYMAYGNLEDYLSDTNSNTLNWATRLRIALEAAQGLEYLHNGCKLQIVHRDVKTTNI
LLNEKFQAKLADFGLSRIFPADGGTHVSTIVAGTPGYLDPEYYITNWLTDKSD
>VFLRR-RLK183
FSNIIGSSQDSFVYKGTMKGGSEIAVISLCIKEEHWTGYLELYFQKEVADLARLNSENTGKLLGY
CSESTPFTRMLVFEYASNGTLYEHLHYGEGCQLSWTRRMKIVIGIANGLKYLHTELDPPFTISEL
NSSAVYLTDDFSAKLVDFESWKSILARSEKNSGSIGSQGAICLLPNSIEGRHLDVQGNVYAFGVL

LLEIISGRPPYCKDKGCLVDWAKEYLEMPEVMSYVVDPELKHFKYEDLKVICEVVSLCIHPEPA
KRPSMEEISSILE
>VFLRR-RLK185
KLGRGGFGIVYKGTLKSGRQVAVKTLSAQSKQGVREFLNEIDTISKVRHPNLVELLGCCIQGTN
RILVYEYVENNSLDQALLGSKNSCIKLDWKKRSAICLGIARGLTYLHEELVPHIVHRDIKASNIL
LDKEFNPKIGDFGLAKLFPDNITHISTRIAGTTGYLAPEYALGGQLTMKADVYSFGVLILEIISGR
SSVRASWGETQKLLLEWAWQLHEEGKHLELVDPELGEFPEEEVIRHMKVAFFCTQGAANRRP
LMSQVVNMLSK
>VFLRR-RLK186
IGVGSYGSVYKGFLEQVGIEAAVKVLNLQRRGALKSFVSECQALRTIRHRNLLKLLNVCSSIDF
EGNDFKALIYEFMEKGSLEEWLHAHNAGEDGQEREFGNLKLIDRVNIAIDIANAIEYLHNGSSS
TIVHGDLKP
>VFLRR-RLK187
KNILGAGGFGNVYKGKLGDGTMVAVKRLKDVSGNAGESQFRTELEMISLAVHRNLLRLIGYC
ATPNERLLVYPYMSNGSVASRLRGKPALDWNTRKRIAIGAARGLLYLHEQCDPKIIHRDVKAA
NVLLDDFCEAVVGDFGLAKLLNHADSHVTTAVRGTVGHIAPEYLSTGQSSEKTDVFGFGILLIE
LITGMRALEFGKTVNQKGAMLEWVKKILQEKKVEELVDRELRSNYDLIEIGEMLQVALLCTQ
YLPAHRPKMSEV
>VFLRR-RLK188
IGTGSFGSVYKGILDKEEGKIIAVKVLNLMHHGAFKSFVAECEALKNIRHRNLVKLITACSSTDY
QGNDFKALIYEFMVNRSLEEWLHPTFELDEATKSLDLLQRLNIAIDVASALQYLHHHCEKTIVH
CDLKPSNILLDDEMIGHVSDFGLAKILFQDTFYSSTNQSSSVGVRGTIGYTPPEYGVGSKVSTY
GDTYSYGILLLEMFTGKRPTDDMFGENMNLHNFVKTNLPKQVVQLIDPILFQEIFNSSKIKNHT
RNIEGIKNNGILECLISIFEIGIACCIDLPQDRMNIGNVVAQLSSI
>VFLRR-RLK189
LDEEDVVGSGGFGTVYRMVMNDCGTFAVKRIDRSREGSDQVFERELEILGSIKHINLVNLRGY
CRLPMAKLLIYDYLAMGSLDDILHEHVQEQPLNWSARLSIALGSARGLAYLHHDCSPKIVHRD
IKSSNILLDENLEPHVSDFGLAKLLVDEDAHVTTVVAGTFGYLAPEYLQSGRATEKSDVYSFGV
LLLELVTGKRPTDPAFVKRGLNVVGWLNTLLRENRLDDVVDKRCKDADMETVEAILEIAARC
TDANPDDRPTMSQVLQLLE
>VFLRR-RLK190
FKEVIGRGSFGSVYLGKLSDGKQVAVKVRFDKTQLGADSFINEVYLLSQIRHQNLVCLEGFCYE
SKQQILVYEYLPGGSLAEHLYGLNSQKVSLSWVRRLKIAVDAAKGLDYLHNGNEPRIIHRDVK
CSNILLDKDMNAKVCDFGLSKQVMQADASHVTTVVKGTAGYLDPEYYSTQQLTEKSDVYSF
GVVLLELICGREPLRHSGTPDSFNLVLWV
>VFLRR-RLK193
EIGKGHIGIVYKAGMPFNVTFAVKKISPQKKQQEKDKIQREIFNLLSLRHENLVQLLGSCSRKG
HHILIYEYMENGSLHQALFEPDSTIELDWKARYDICLGIAKGLKYLHEDKRFEIVHGNITARNIL
LDKNHTPKISDFGLARFRDDEDAFTTIKTRERLYVAPEYFLGKAITVKADVYSFGVVALEIVSGR
TSMEQRPNQEYDVLLDTAYVLYAKGKILNLVDEKLSSSYDRKQALILLDIAIMCINSQSPSLRPK
MSDVVSVL
>VFLRR-RLK194

IGKGGYGTVYKANLPSGSTVAVKKLHPLGNGERPYQKEFLNEIRALTEIRHRNIVRLHGFCSYA
SHSFLVYEYLEGGSLAAVLGNDKEAKELDWSKRVNIVKGVAQALSYMHHDCSPPIVHRDIKSK
NILLDSEYEAHISDFGTAKLLNLDSSHWTALAGTYGYVAPEFAYTMK
>VFLRR-RLK195
IGKGGYGTVYKANLPSGSTVAVKKLHPLGNGERPYQKEFLNEIRALTEIRHRNIVRLYGFCSYA
RHSFLVYEYLEGGSLAAVLGNDKEAKELDWSKR
>VFLRR-RLK197
FEKKIGSGGFGVVYYGKMKDGKEIAVKVLTSNSFQGKREFSNEVTLLSRIHHRNLVQFLGFCQ
DDGRSMLVYEFMHHGTLKEHLYGPLTHGRNINWIKRLEIAEDAAKGIEYLHTGCVPAIIHRDLK
TSNILLDKHMRAKVSDFGLSKLAVDGVSHVSSIVRGTVGYLDPEYYISQQLTDKSDVYSFGVIL
LELMSGQEAISNESFGVNCRNIVQWAKLHIESGDIQGIIDPSLNDEYDIQSMWKIAEKALMCVQ
PHGHMRPSISEVLKEIQ
>VFLRR-RLK199
LAEGKFGPVYRGFLPGGIHVAVKVLVHGSTLTDQEAARELEYLGRIKHPNLVPLTGYCIAGDQR
IAIYDYMENGNLQNLLHDLPLGVQTTEDWSTDTWEEDDNNGIQNVGSEGLLTTWRFRHKIAL
GTARALAFLHHGCSPPLIHRDVKASSVYLDYNLESRVSDFGLAKIFGNGLDEEIARGSPGYVPP
EFSDPDNNSPTPKSDVYCFGVVLFELITGKKPVGDDYPEEKEATLVSWVRGMVRKNQGSRTID
SKIRDTGPEYEMEETLKIGYLCTADIPSKRPSMQQIVGLLKDI
>VFLRR-RLK200
LAEGKFGPVYRGFLPGGIHVAVKVLVHGSTLTDQEAARELEYLGRIKHPNLVPLTGFCIAGDQRI
AIYDYMENGNLQNLLHDLPLGVQTTEDWSTDTWEEDDNNGIQNVGSEGLLTTWRFRHKIALG
IARALAFLHHGCSPPLIHRDVKASSVYLDYNLESRVSDFGLAKIFGNGLDEEIARGSPGYVPPEF
SDPDNNSPTPKSDVYCFGVVLFELITGKKPVGDDYPEEKEATLVSWVRGMVRKNQGSRTIDSK
IRDTGPEYEMEETLKIGYLCTADIPSKRPSMQQIVGLLKDI
>VFLRR-RLK201
HFGKESLLSEGRCGPLYRAVLPGDVHVAIKVLENARGIDYEEAVTIFEGLSRLKHPNLLPLCGYC
IAGKEKLVLYEFMANGDLHRWLHELPTLVPNVEDWSTDTWEHQNISGSHIFSPEEKTNWLTRH
RIAVGVARGLAYLHHAGSIHGHLVASNILFSDTLEPRVADFGLRNISPKNKHIVENNGIEFDVYCF
GVVLIELLTGKQGSEETVEWVRRLVREGRGADALVPTLTVSGESVSEMVECLRVGYLCTAELP
GKRPSMQQVLGLLKDI
>VFLRR-RLK204
IGIGHFSSVYKGKLKDGKEVAIKAFNVEEETSLRSFDLECEVFSRVSHPNLVRTVCISNSINFKAL
VMEYMANGSLEKWLHTHKYDLDILQRFDIMIETAKAVRYLHQRCIIHCDIKPSNIILDEDMVAH
VSDFSIAKIQPVSRIRTIQSKLMCTIGYVAPEYGRYGIVSASMDVYSFGILLMETFTGKKPTHEM
FTGEMNLRRWVIESLPCEVERVIDPSLLQTEEENNDVYMKCISSVMRLALICSAESPSERLNMR
DVEEKLNNI
>VFLRR-RLK205
IGIGHFSSVYKGKLKDGKEVAIKVFNVEEETSLRSFDLECEVFSRVSHPNLVRTVCISNSINFKAL
VMEYMANGSLEKWLHTHKYDLDILQRVDIMIETAKAVRYLHQRCIIHCDIKPSNIILDEDMVAH
VSDFSIAKIQPVSRIRTIQSKLMCTIGYVAPEYGRYGIVSASMDVYSFGILLMETFTGKKPTHEM
FTGEMNLRRWVIESLPCEVERVIDPSLLQTEEENNDVYMKCISSVMRLALICSAESPSERLNMR
DVEEKLNNI
>VFLRR-RLK206

IGSGGSGQVYKVKLKSGQIVAVKRLWGGNNKKPETESVFRSEVETLGRVRHGNIVKLLMCCSG
EEFRILVYEYMENGSLGDVLHGEKDGGLMDWTERFAVAVGAAQGLAYLHHDCVPPIVHRDVK
SNNILLDEEMRPKVADFGLAKTLQSDVGEGDAVMSRIAGSYGYIAPEYAYTLK

>VFLRR-RLK208

KFNEINLLGRGSFGSVYKGTLHDGMNVAVKVFNLQLEAGFKSFDVECEVLRRIRHRNLVKIITA
CCNIDFKALVLEFMPNWSLEKWLYSHNYFLDILQRMNIMVDVALALEYLHYGSTTPVVHCDL
KPSNILLDEGMVGHVSDFGIAKLLGEENSIIQTMTLATVGYMAPEYGSEGLVSIKGDTYSYGIV
VMETFTRKRPTDEMFTEEMSMKQWVRDSLSLPCGVTEIADSNLLRHNEQHFFAKKDCIESTLL
LAQKCCADLPEERTDIRDVLSTLKKI

>VFLRR-RLK212

ILGATGASIVYKAVLADGTAFAVRRIGESGVERFRDFENQVRIIAKLRHPNLVKVRGFYWGNDE
KLVLYDYVSNGSLASSNYKKPGSSPFHLPLEVRFKIARGLARGLAFIHEKKHVHGSIKPSNILLN
FDMEPIISDFGLDRLVLGNNSYKASNSGRNFGSQRSNSTPQDHPITASPYATPSSSSSTTITSPYQA
PESLKNLKPNPKWDVYAFGVILLELLTGRVVSDRELSQWTAGLIVEDKDRVLRLADVAIRVDVE
AKEDAMLACFKLGFSCVSFVPQKRPSMKEAVQVLEKI

>VFLRR-RLK213

FDTMNFMGHGYQGQLYKGWLRDGLMVLIKCVKLKQKNLPQSLVQHMEVLSKLRHLHLVSV
LGHCIVTYQDHPRTASSTVFLVLEQISNGSLQDYLKDWRKKDVLKWPQRMAITIGIARGIQFLH
TGVAPAIFGNNIKIENVLLDESLTAKLSNYNIPLPSKVGSESPLYGQDSINAEKEDVYQLGVILVQ
VITGKIVTSPSELEELKIQLEKGLAESPLKLQAMVDPSTRGTFAYESLRTTVKITINCISKELSNRP
SIEDVL

>VFLRR-RLK218

IGEGGTGKVYKVEMPGSQVLAVKKLNYSGKDGEVSRIKIFSSEVAALAELRHRNIVKLHGFYA
RGKHTFLAYEFIENGSLANMLSSEKGASELNWEKRIKIVKGVAHALSYMHHDCNPPIIHRDISS
NNILLNSELEAYVSDFGTARFWKPDLSNWTTIAGTYGYVAPELAYTAAVTEKCDVYSFGVLTLE
VLIGKQPGELISYLQSLTHLEDVLDGRLSLPSDQQLADKLSCVLTIAFTCLRPNPQSRPSMRNVS
QLLD

>VFLRR-RLK219

IGEGGCSNVYKGCLRKCKPVAIKVLKQYKEAWNDFSFEVDIMSSLKHKNITHLIGVCIEDDHLI
LVYDFLSKGSLEERLQG

>VFLRR-RLK220

LGEGGYGPVYKGTLSDGRKVAVKLLSLASNQGKSEFVTEIATISAVQHRNLVKLYGCCIEGNRR
LLVYEYLENKSLDKALFATCAGKTSLHLDWPTRFDICLGTARGLAYLHEESRPRIVHRDVKA

>VFLRR-RLK222

FKEVIGRGSFGSVYLGKLSDGKPVAVKVRFDKTQLGADSFINEVYLLSQIRHQNLVCLEGFCYE
SKQQILVYEYLPGGSLAEHLYGLNSQKVSLSWVRRLKIAVDAAKGLDYLHNGNEPRIIHRDVK
CSNILLDKDMNAKVCDFGLSKQVMQADASHVTTVVKGTAGYLDPEYYSTQQLTEKSDVYSF
GVVLLELICGREPLRHSGTPDSFNLVLWAKPYLQAGAFEIVDDSIKGTFDVESMRKAATVAVRS
VERDAAQRPNIAEVLAELKE

>VFLRR-RLK225

HFGKESLLSEGRCGPLYRAVLPGDVHVAIKVLENARGIDYEEAVTIFEGLSRLKHPNLLPLCGYC
IAGKEKLVLYEFMANGDLHRWLHELPTLVPNVEDWSTDTWEHQNISGSHIFSPEEKTNWLTRH
RIAVGVARGLAYLHHAGSIHGHLVASNILFSDTLEPRVADFGLRNISPKNKHIVENNGIEFDVYCF

GVVLIELLTGKQGSEETVEWVRRLVREGRGADALVPTLTVSGESVSEMVECLRVGYLCTAELP
GKRPSMQQVLGLLKDI
>VFLRR-RLK228
LGRSSHGTSYRATLDNGMFLTVKWLREGVAKQKKEFAKEAKKFANIRHPNVVGLRGYYWGP
TQHEKLILSDYISPGSLASFLYDRPGRKGPPLTWVQRLKIAVDVARGLNYLHFDRAVPHGNLKA
TNILLDGPDLNARVADYCLHRLMTQAGTIEQILDAGVLGYRAPELAASKKPLPSFKSDVYAFG
VILLELLTGRCAGDVISGEEGGVDLTDWVRLRVTEGRGSDCFDPALMPEMSNPAVEKGMKE
>VFLRR-RLK230
LNEEHVIGFGGFGTVYKLAMDDGNVFALKRILKMNEGFDRFFERELEILGSIKHRYLVNLRGY
CNSPTSKLLIYDYLPGGSLDEALHERSEQLDWDARLNIIMGAAKGLAYLHHDCSPRIIHRDIKSS
NILLDGNLEARVSDFGLAKLLEDEESHITTIVAGTFGYLAPEYMQSGRATEKTDVYSFGVLVLE
VLSGKRPTDASFIEKGLNIVGWLNFLVTENRPREIADPNCEGVQIESLDALLLVATQCVSSSPED
RPTMHRVVQILE
>VFLRR-RLK232
VLKLASSLTPLPTGWSTKSSSGFCNWKGIECDSSNRVTSISLPNKDLTGSLPSELSTLTQLKTLSL
QNNHLSGDLPLLANFSNLQSIFLDFNNFTSIPSGFFKGLTSLQTLSLGNNVNLAPWQLPTDLTES
TTLNSITASQSNIYGSIPDIFGSLPSLQSLRLSYNNLTGSLPQSMANSGIQNLWINNQQIGLTGTIG
VLATMTQLSQVWLQKNQFTGSIPDLSKCDSLFDLQLRDNQFTGVVPSSLISLPNLVNISLSNNK
LQGPSPVFSSSVKVVNDGKNNYCTVSGKACDPQVTTLLEIAQGFGYPAKLSDGWTGNDACSG
WDFVTCDPQKRVITVSLGKQGLTGTISPAFANLTSLKNLYLNDNNLTGSIPASLTQLTQLQTLDV
SNNNL
>VFLRR-RLK234
KIGEGGFGSVYKGVLPNGMEIAVKQLSSKSKQGTREFVNEVGTIFALQHPNLVKLLGCCTEDN
QLLLVYEYMENNSLAHALFGSEELRLKLKWPIRFKICLGIAKGLAFLHEESKLKVVHRDIKPTN
VLLDKDLNAKISDFGLAKLYEAEKTHIITRIAGTTGYMAPEYAMRGYLTNKADVYSFGVVALEI
VSGKNGTSYRPNDESVYLLDLAYVLQEKGDLLSLVDPMLGCDYSVKQAMTILDLAMLCTNPS
PTLRPTMSEVVNVLEG
>VFLRR-RLK240
EVRILGALQHSCIVELYGHQISSKWVPSEEGKPERQILQSAILMEYVKGGSLKSYIEKASKTGEK
HVPVELALCIARDVAYALAELHSKHIIHRDVKSENILIDVENKRADGMPVVKLCDFDRAVPLRS
FLHTCCITHRGVPPPDVCVGTPRWMAPEVLQAMHKRSLYGLEVDIWSYGCLLLELLTLQVPYS
GLSEFHINELLQTGKRPPLTKELETLASMNEPATTQPGSNLAGAEAESETLRFLVDLFHRCTEGN
PANRPTAAEIYELL
>VFLRR-RLK241
IGGGGFGVVYRGVLRDGTQVAIKCLSAESKQGKHEFMTEIKIISYIRHPNLVELVGCCVEGSNRI
LVYEYMENNSLATSLLGSKGKHIAMDWSKRAAICLGTATGLAFLHEGIEPPIVHRDIKASNILLD
GNLQPKIGDFGLAKLFPDNVTHVTTRVAGTVGYLAPEYALLGQLTKKADVYSFGVLLLEIISGR
SSSKAAFGVDLLVLVEWTWKLREEGRLLELVDPELPEYPEDEVMRFIKVALFCTQAAGRQRPT
MKQVVEMLSK
>VFLRR-RLK242
IGSFSYGTVYKGTLSSGVEIAVTSTTVISRKEWSKILEAQFRKKIDTLSKMNHKNFVNLIGYCEE
NEPFTRIMVFEYAPNGTLFEHLHIKEAEHLDWGMRLRIAMGMAYCLEYMHQLTPPIVHDNLQS
SSIYLTEDYAAKISDFSYWNDVTTKIASIELIKPSSEYPESNVYSFGLILYEMITGRIPY
>VFLRR-RLK243

IGKGGFGTVYRGVLPDGRQVAVKKLQREGIEGEREFRAEMEVLSGNGFGWPHPNLVTLYGWC
LDGSEKILVYEYMEGGSLEDLVSDRMRLTWKKRIDIAIDVARALVFLHHECYPAIVHRDVKASN
VLLDKDGKARVTDFGLARFVDAGDSHVSTMVAGTVGYVAPEYGQTWQATTKGDVYSFGVLA
MELATGRRAVDGGEECLVEWARRVIGNARNGLGRAVIPVVLLGSGLAEGAVEMCELLRIGIRCI
AETPQARPNMKE
>VFLRR-RLK244
LIGSGGFGEVYKAQLKDGCVVAIKKLIHVTGQGDREFMAEMETIGKIKHRNLVPLLGYCKVGD
ERLLVYEYMKWGSLEAVLHDRSKGGCSRLDWAARKKIAIGSARGLAFLHHSCIPHIIHRDMKS
SNVLLDENFEARVSDFGMARLVNALDTHLSVSTLAGTPGYVPPEYYQSFRCTTKGDVYSYGVI
LLELLSGKKTIDPSEFGDDNNLVGWAKQLHREKRSDEILDPELTAQKSFEAELHQYLRISFECLD
DRPFKRPTMIQVMAMFKE
>VFLRR-RLK245
EVLGSGGFGKVYRAVLPSDGTVVAVKCLAEKGEQFEKTFAAELVAVAHLRHRNLVRLRGWCV
HEDQLLLVYDYMPNLSLDRVLFRRPENLTATPLDWERRRKIIGGLAAALHYLHEQLETQIIHRD
VKTSNVMLDSHYNARLGDFGLARWLEHELEYQTRMPSMRNHQFRLADSTRIGGTIGYLPPES
FQKRSVATAKSDVFSFGIVVLEVVSGRRAVDLTYPDDKIILLDWVRRLSDEGKLLQAGDNRLPD
GSYALSDMEQLIHLGLLCTLHIPQLRPNMKWIVETLVA
>VFLRR-RLK246
IGKGGFGTVYRGVLPDGRQVAVKKLQREGIEGEREFRAEMEVLSGNGFGWPHPNLVTLYGWC
LDGSEKILVYEYMEGGSLEDLVSDRMGLTWKKRIDIAIDVARALVFLHHECYPAIVHRDVKASN
VLLDKDGKARVTDFGLARFVDAGDSHVSTMVAGTVGYVAPEYGQTWQATTKGDVYSFGVLA
MELATGRRAVDGGEECLVEWARRVIGNARNGLGRAVIPVVLLGSGLAEGAVEMCELLRIGIRCI
AETPQARPNMKEVLAMLIKI
>VFLRR-RLK248
QLGEGGYGPVYKGTLSDGRKVAVKLLSLASNQGKSEFVTEIATISAVQHRNLVKLYGCCIEGNR
RLLVYEYLENKSLDKALFATCAGKASLHLDWPTRFDICLGTARGLAYLHEESRPRIVHRDVKA
SNILLDAELCPKISDFGLAKLYDEKKTHISTRVAGTIGYMAPEYAMRGHLTEKADVFSFGVLAL
EILSGIPNYENNLVEEKIYLLGWAWTLYEKNQSLALVDPSLMGFDENEALRVLRVALLCTQASP
TVRPTMSRVVAML
>VFLRR-RLK249
VGQGGYGKVYKGILADGTVVAIKRAQEHSLQGEKEFLTEIELLSRLHHRNLVSLTGYCDEEGE
QMLVYEFMPNGTLRDHLSAKSKVPLSFATRLRTALGSAKGILYLHTEADPPIFHRDIKASNILLD
SNFNAKVADFGLSRLAPVPDDEGAAPAHVSTVVKGTPGYLDPEYFLTHKLTDKSDVYSLGVVF
LELLTGMQAISHGKNIVREVNVSYQSGMIFSVIDGRMGSYPSDCVEKFLNLAMKCCKDETDAR
PSMADVVRELETI
>VFLRR-RLK250
VGHGGSGTVYKIELSNGEVVAVKRLWSKRTKDSSSEDQLLLDKNLKAEVGTLGSIRHKNIVKL
YCYFSSLNCSLLVYEYMSNGNLWDALHKNKIYLDWPTRHQIALGVAQGLAYLHHDLLSPIIHR
DIKSTNILLDVNYQPKVADFGIAKVLQARAGKDSTTTIVAGTYGYMAPEYAYSSKATTKCDVY
SFGIVLMELITGKKPIHADFGENKNIVYWVSAKVDTKEGVMEVLDKRLSGSFRDEMIQVLRIAI
RCTCNTPALRPTMNEVVQLLID
>VFLRR-RLK251
QVGQGGYGKVYKGILADGTVVAIKRAQEHSLQGEKEFLTEIELLSRLHHRNLVSLTGYCDEEG
EQMLVYEFMPNGTLRDHLSAKSKVPLSFATRLRTALGSAKGILYLHTEADPPIFHRDIKASNILL

DSNFNAKVADFGLSRLAPVPDDEGAAPAHVSTVVKGTPGYLDPEYFLTHKLTDKSDVYSLGV
VFLELLTGMQAISHGKNIVREVNVSYQSGMIFSVIDGRMGSYPSDCVEKFLNLAMKCCKDETD
ARPSMADVVRELETI
>VFLRR-RLK252
KTRFSTYYKATMPSGASYFVKKLNWSDKIFQLGSHDKFDQELEVLGKLSNSNVMTPLAYVLT
VDSAYLFYEHAQKGTLFDVLHGKLGNALDWGSRYSIAVGVAQGLTFLHGYTSGPILLLDLSSR
NILLKSLKEPLVGDIELYKVIDPTKSTGSLSTVAGSVGYIPPEYAYTMRVTMAGNVYSFGVVLLE
LLTGKPAVSEGTELAKWVSSNSAQKDRWDHILDFSISRTSLAVRGQMLAMLKVALSCVSLSPEA
RPKMKSVLRMI
>VFLRR-RLK254
EIGKGHIGIVYKAGMPFNVTFAVKKISPQKKQQEKDKIQREIFNLLSLRHENLVQLLGSCSRKG
HHILIYEYMENGSLHQALFEPDSTIELDWKARYDICLGIAKGLKYLHEDKRFEIVHGNITARNIL
LDKNHTPKISDFGLARFRDDEDAFTTIKTRGERLYVAPEYFLGKAITVKADVYSYGVVVLEIVS
GRTSMEQRPNQEYDVLLDAACVLHARGKILDLVDKKLSSSYDRKQALILLDTAIKCINSQSPTL
RPKMSDVVSSL
>VFLRR-RLK255
LGAGGFGAVYKGTLKDGTIIAVKQLSSRSKQGNREFLNEIGMISVLQHPNLVRLYGCCVEGNQL
LLVYEYMENNSLAHVLFDAEESQSKLDWATRQRICVGIAKGLAFLHEESALKIVHRDIKTANIL
LDGDLNPKISDFGLAKL
>VFLRR-RLK256
EIGRGRFGIVYKAEMPDQTKLAVKKISPESTQDKLKDELQGEIFFNVKSLEHENLIQLFDGYSK
KDLHLLIYEYMENGSLDQALFEPKSRIELNWEVRFNICLGLAKALKYLHEENVRKIIHRNIKAS
NILLDENYTAKLTDFGLASFDNEDDPFKTIKAGGARAYFAPEYARGQAITDKADVYSFGVVTLE
IFSGRSCTENKSEAQKYLLDDACIFQARGKIDQLVDKKLANYDRKLVPKILNLAIMCINPTADL
RPTMSEVVSVL
>VFLRR-RLK257
IGNGGFGATYKAEISSGVLVAIKRLAVGRFQGVQQFHAEIKTLGRLHHPNLVTLIGYHASETEM
FLIYNYLPGGNLEKFIQERSTRAVDWRILHKIALDIARALAYLHDECIPRVLHRDVKPSNILLDD
DFNAYLSDFGLARLLGTSETHATTGVAGTFGYVAPEYAMTCRVSDKADVYSYGVVLLELLSDK
KALDPSFSSYGNGFNIVAWACMLLRQGRAKELFTAGLWDAGPHDDLVEVLHLAVVCTVDLLS
TRPTMKQVVRRLKQL
>VFLRR-RLK258
IGEGGFGPVFKGLLSDGTIIAVKQLSSKSKQGNREFVNEIGMISALQHPHLVKLYGCCIEGNQLL
LVYEYMENNSLARALFGPEEHQLNLDWSTRHKICVGIARGLAFLHEESRLKIVHRDIKATNVLL
DKYLNSKISDFGLAKLDEEENTHISTRVAGTFGYMAPEYAMRGYLTDKADVYSFGIVALEIVSG
RCNTSNRSNMKEECFYLLDWACVLKEKGNLLELVDPKLGKNYNKEQVMTMINVALLCTNVS
PVVRPAMSSVVSMLEG
>VFLRR-RLK260
EIGRGRFGIVYKAEMPDQTKLAVKKISPESTQDKLKDELQGEIFFNVKSLEHENLIQLFDGYSK
KDLHLLIYEYMENGSLDQALFEPKSRIELNWEVRFNICLGLAKALKYLHEENVRKIIHRNIKAS
NILLDENYTAKLTDFGLASFDNEDDPFKTIKAGGARAYFAPEYARGQAITDKADVYSFGVVTLE
IFSGRSCTENKSEAQKYLLDDACIFQARGKIDQLVDKKLANYDRKLVPKILNLAIMCINPTADL
RPTMSEVVSVL
>VFLRR-RLK261

FDPANKIGEGGFGPVFKGLLSDGTIIAVKQLSSKSKQGNREFVNEIGMISALQHPHLVKL
YGCCIEGNQLLLVYEYMENNSLARALFGPEEHQLNLDWSTRHKICVGIARGLAFLHEESR
LKIVHRDIKATNVLLDKYLNSKISDFGLAKLDEEENTHISTRVAGTFGYMAPEYAMRGYL
TDKADVYSFGIVALEIVSGRCNTSNRSNMKEECFYLLDWACVLKEKGNLLELVDPKLGKN
YNKEQVMTMINVALLCTNVSPVVRPAMSSVVSML
>VFLRR-RLK262
FNPANKLGAGGFGAVYKGTLKDGTIIAVKQLSSRSKQGNREFLNEIGMISVLQHPNVVRL
YGCCVERNQLLLVYEYMENNSLEHALFGTEECLSKLDWPTRQRICIGIAKGLAFLHEESA
LKIVHRDIKAANILLDRDLNPKISDFGLAKLDEEENTHISTR
>VFLRR-RLK266
HGHSGDLFRGTLENGISVVIKRVDMQSIKKETYLLELDFFSKVSHPRLVPLLGHCLENENEKFLI
YKYMPNGDLSSSLYRKTSEEDGSLQSLDWITRLKIATGAAEGLSCLHHECTPPIVHRDVQASSIL
LDDKFEVRLGSLSEACPQEGDTHQSRITRLLRLPQSSEQGASGSVTTTCAYDVYCFGKVLLELV
TGKLGMSASSEAQLKEWLEQTLPYISMYEKELVTKIVDPSLIIDEDLLEEVWAMAIVARSCLNP
KPSRRPPMRYILKALEN
>VFLRR-RLK269
KIGEGGFGPVYKGLLPDGTVIAVKQLSSKSSQGNREFLNEIGMISCLQHPNLVKLHGCCVEGNQ
LLLVYEYMENNSLARALLGPEHSRIKLDWQTRQRICVGIARGLAFLHEESRLKIVHRDIKATNV
LLDKHLNPKISDFGLAKLDTEEKTHISTRIAGTMGYMAPEYALWGYLTHKVDVYSFGIVALEIV
SGKHNMSHGPENNFACLLDWACHLQQSGNLMELVDEKLGSDFKKKEAERMIKASLLCTNGSP
SLRPSMSEVVNMLEG
>VFLRR-RLK270
GHSGDLFRGFLDGGSTIVVKKVNLPSLKKESYMMELELFSKYSHTRLVPFLGHCSENDNEKLL
VYKYMPNADLASSLYRVSEFEDDNLQSLDWITRLKIAIGAAEGLSYLHHECNPPLVHRDIQASS
ILLDDKFEVRLG
>VFLRR-RLK271
IGEGGFGPVYKGLLPDGTVIAVKQLSSKSSQGNREFLNEIGMISCLQHPNLVKLHGCCVEGNQL
LLVYEYMENNSLARALLGPEHSRIKLDWQTRQRICVGIARGLAFLHEESRLKIVHRDIKATNVL
LDKHLNPKISDFGLAKLDTEEKTHISTRIAGTMGYMAPEYALWGYLTHKVDVYSFGIVALEIVS
GKHNMSHGPENNFACLLDWACHLQQSGNLMELVDEKLGSDFKKKEAERMIKASLLCTNGSPS
LRPSMSEVVNMLEG
>VFLRR-RLK273
IGVGGYGIVYKVVLPTGRVVAVKKLHQSQNGEMDDFKAFKSEICVLMNIRHRNIVKLYGFCSH
VKHSFLVYEFIERGSLRNVLSNEEQAMELDWFKRLNVVKGIANALAYMHHDCCPPIIHRDISSN
NVLLDSEFEAHVSDFGTARLLMPDSSNWTSFAGTFGYTAPELAYTMAVNEKCDVYSFGVVTLE
IIMGRHPGDFISSLSLSSSSLTLPIDQHTLEDVIDQRLPFPRNKAANGLIHTIKLALACLSANPQSR
PTMRQVSSQL
>VFLRR-RLK274
IGKDGRFGIIYKAELSNLTVAVKKLFPQSKAVAQIGTEVYAKTFKLEHDNLVQLLATYSRKDLHL
LIYEYMGYGSLEKALFDPKSSIQLSWEDRYSICLQIAQGLEYLHAKNPPIIHRNIKASNVLLDET
CKAKISDFGLAKLYEEDDPFRFIEESGTRMYMAPEYATRKAVTMGIDVYSFGVLLLEIISGIKND
ENLSEHHDQTIFLLEKVANLHVMVKDAKLQYKKVKYAELVDKKISNLNYSKEEVVTIVDLAM
LCTDQMVSLRPTISDVTSVLKG
>VFLRR-RLK275

KLGEGGFGPVYKGILNDGRVIAVKQLSVASHQGKSQFVTEIATISAVQHRNLVKLYGCCIEGYN
RLLVYEYLENKSLDQALFGETSLNLDWPTRYQICLGVARGLAYLHEESRLRIVHRDVKASNILL
DSELIPKISDFGLAKLYDDKKTHISTRVAGTIGYLAPEYAMRGHLTEKADVFAFGVVALEVISGR
PNSDSSLEEEKIYLLEWAWNLHENNREVELVDSKLSDFSEEDVKRLIGVALLCTQTSPNLRPSM
SRVIAML

Amino acid sequences of RLK domain from *V. Montana*

>VMLRR-RLK5
LGKGGFGTVYHGSLDDTEVAVKMLSPSSVQGYKQFQAEVKLLLRVHHRNLTTLVGFCNEDTY
MGLIYEYMANGDLENLLSGSNRNVLRWDMRLQIVVEAAKGLEYLHSGSKPPIVHRDVKTANI
LLNDKFQAKLADFGLSRSFP
>VMLRR-RLK8
VYRVETPLKQVIAVKKLWPVKNGEIPERDWFSAEVRTLGSIRHKNIVRLLGCCNNGKTRLLLFD
YISNGSLAGLLHEKKLFLDWDARYNIILGAAHGLEYLHHDCTPPIVHRDIKANNILVGPQFEAF
LADFGLAKLVDSAECSRVSNTVAGSYGYIAPEYGYSFRITEKSDVYSYGVVLLEVLTGENQLIL
RFLKELRERKRDFTTILDQQLLLRSGTQLQEMLQVLGVALLCVNPSPEERPTMKDVTALLKEI
>VMLRR-RLK11
IGGGSIGTVYRTNFEGGISIAVKKLETLGRIRSQDEFEQEIGRLGNLRHPNLVSFQGYYWSSSMQ
LILSEFVPNGNLYDNLHGLDYPGTSTGVGNSELYWSRRFQIALGTARALSYLHHDCRPPILHLNI
KSTNILLDENYEAKLSDYGL
>VMLRR-RLK14
QEAARELEYLGRIKHPNLVPLTGFCIAGDQRIAIYDYMENGNLQNLLHDLPLGVQATEDWSTD
TWEEDDNNGIQNVGSEGLLTTWRFRHKIALGIARALAFLHHGCSPPLIHRDVKASSVYLDYNL
EPRVSDFGLAKIFGNGLDEEIARGSPGYVPPEFSDPDNNSPTPKSDVYCFGVVLFELITGKKPVG
DDYPEEKEATLVSWVRGMVRKNQGSRTIDPKIRDTGPEYEMEETLKIGYLCTADIPSKRPSMQ
QIVGLLKDI
>VMLRR-RLK15
IGRGGAGIVYKGIMPNGEQVAVKKLLGISKGSSHDNGLSAEIQTLGKIRHRNI
>VMLRR-RLK47
FSIRNLIGTGGFGSTYKAELAPGYLVAVKRLSLGRFQGIQQFDAEIRTLGRIRHKNLVTLIGYYV
GEAEMFLIYNYLSGGNLETFIHERSSKNVQWSVIYKIAFDIAQALAYLHYSCVPRILHRDIKPSN
ILLDEELNAYLSDFGLAKLLEVSQTHATTDVAGTF
>VMLRR-RLK48
KIGEGGFGSVYKGRLKDGKITAIKVLSAESRQGVKEFLTEINVISEIEHENLVKLYGCCVEGNHR
ILVYNFLENNSLAQTLLGVGNSYSNIQFSWRTRSRICIGVARGLAFLHEDLRPHIVHRDIKASNIL
LDKDLSPKISDFGLAKLIPPNMTHVSTRVAGTIGYLAPEYAIRGQLTRKADIYSFGVLLVEIVSGR
CNTNTRLPIEEQYLLERTWELYERKELVGLVDTALDGDFDAEEACKFLKIGLLCTQDAPKLRPS
MSTVVKLLTG
>VMLRR-RLK51
IGKGGFGSVYKAVLSTGQVVAVKKLNISDSSDIPAINLQSFENEIRMLTEVRHRNIIKLYGYCSRR
GCLHLVYEYVERGSLGKVLYGVEGEIDLGWATRVKIVQGVAHAIAYLHHDCSPPIVHRDISMN
NILLESDFEPRLSDFGTARLLNADSSNWTAVAGSYGYMAPELALTMRVTDKCDVYSFGVVALE
VMMGRHPGELLSSISSPKPSMSNDPDLFLKDVLDQRLPSPTGQLAEEVVFVVQVAIACTRTTPE
ERPAMRFMAQEL

>VMLRR-RLK55
LGRSSHGTSYRATLDNGMFLTVKWLREGVAKQKKEFAKEAKKFANIRHPNVVGLRGYYWGP
TQHEKLILSDYISPGSLASFLYDRPGRKGPPLTWVQRLKIAVDVARGLNYLHFDRAVPHGNLKA
TNILLDGPDLNARVADYCLHRLMTQAGTIEQILDAGVLGYRAPELAASKKPLPSFKSDVYAFG
VILLELLTGRCAGDVISGEEGGVDLTDWVRLRVTEGRGSDCFDPALMPEMSNPAVEKGMKEVL
GLALRCIRSVSERP
>VMLRR-RLK56
IGSGGFGATYKAEIVPGVVVAVKRLSVGRFQGVQQFATEIRTLGRVQHPNLVKLIGYHLSETEM
FLIYNYLPGGNLETFIQERSRRVVEWSMLHKIALDIARALAYLHDECVPRVLHRDIKPSNILLDN
NFNAYLSDFGLARLLGTSETHATTDVAGTFGYVAPEYAMTCRVSDKADVYSYGVVLLELISDK
KALDPSFSSFGNGFNIVAWASMLLRQGRASEFFTAGLWDSGPHNDLIQILHLGIMCTGESLSSRP
SMKQVAQRLKRI
>VMLRR-RLK59
YKAVLEDGTALAVRRIGENHVERFRDFETQVRVIAKLVHPNLVRIRGFYWGVDEKLIIYDFVPN
GSLSNARYRKVGSSPCHLPWEARLKIAKGVARGLSFLHEKKHVHGNLKPSNILLGSDMEPRIG
DFGLERLVTGDSSHKSSGSTRNFGSKRSTASRDSFQDFPLGPSPSPSPSSIGGLSPYHAPESLRSIK
PNPKWDVYSFGVILLELLTGKVIVVDELGQGNNGLVVDDKNRAMRMADVAIRADVDGKEESL
LACFKL
>VMLRR-RLK60
LGRGRSGIVYMVTLPSGSTVAVKRFKTMDKFSAAAFSSEIATLARIRHRNIVRLLGWGANRKTK
LLFYDYMSNGTLGALLHEGSIVGLVEWETRFKIALGVAEGLAYLHHDCVPPILHRDVKAHNIL
LGDRYEACLADFGLARLVEDDQGSFSATPQFAGSYGYIAPEYACMLKITEKSDVYSYGVVLLEI
ITGKKPVDPSFAEGQHVIQWVREQLKSKKDPVEILDPKLQGHPDTQIQEMLQALGISLLCTSNR
AEDRPTMKDVAALLREI
>VMLRR-RLK64
LSRTRYGLVFKACYNDGMVLSIRRLPDGLMDENMFRKEAEFLSKVKHRNLTVLRGYYAGQQQ
DLRLVVYDYMPNGNLATLLQEASHQDGHVLNWPMRHLIALGIARGLAFLHTSNMVHGDVKP
QNVLFDADFEAHLSDFGLDRLTIATPAEASSSTTVGTLGYVSPEAILTGEVSKESDVYSFGIVLLE
LLTGKRPVMFNEDEDIVKWVKKQLQRGQITELLEPGLLELDPESSEWEEFLLGVKVGLLCTAP
DPLDRPTMSDIVFMLEG
>VMLRR-RLK66
GTAYRAKFQDGDIALVKEVKDLSQEKDVFIRQVQLLGRLHHRHLLALKGFSTGHKRLLVFDNI
ENGSLKEHLNDPLRTPLNWKTRLRIAIGVVAALEYLLLFSNPPMYHVSISSSNIMLDENFTAKLS
NVGLLSSIENYVTMPQASCAEDCMNQNCGNIIFQLGVLILELITGQSSEKGSTDLVQWIQGSRF
GSSIQKMIDPDLGNSYDSRELKNLLAVARLCIKSGDKPKFSIPQIFRYLQK
>VMLRR-RLK67
LGRSSHGTSYRATLDNGMFLTVKWLREGVAKQKKEFAKEAKKFANIRHPNVVGLRGYYWGP
TQHEKLILSDYISPGSLASFLYDRPGRKGPPLTWVQRLKIAVDVARGLNYLHFDRAVPHGNLKA
TNILLDGPDLNARVADYCLHRLMTQAGTIEQILDAGVLGYRAPELAASKKPLPSFKSDVYAFG
VILLELLTGRCAGDVISGEEGGVDLTDWVRLRVTEGRGSDCFDPALMPEMSNPAVEKGMKEVL
GLALRCIRSVSERP
>VMLRR-RLK70
VISRGKKGVLYKGKSILLSDMQFMVKEINDLNSIPSNFWPEVSEFGKVKHPNIVKLIGMCRSDK
GGFLVYEHVEGKDLSEILRNLSWERRRKIAIGIAKALRYLHGYCSPSIIVGHMSPEKILVDGKDE

PRLRLSLPDLLCIDTKCFISSAYVAPETRDSNDINEKSDMYGFGLILIELLTGKAPGDREFGVHESI
VEWARYCYSDCHLDTWIDEMIKAEALMNQNEIVETMNLALHCTATDPKARPCAIDMFKTLN
>VMLRR-RLK74
IGRGGQGSVYKAILPSGDIFAVKRLHPSEDNVSSDEYQMKTFKSEMYSLTEIRHRNIVKMHGFS
YLNGSLYFVYEFARGGSLGEWLQEEKKAKILNWDLRLKVIKGVANALSYLHHDCTPAVVHRDI
SGSNILLDAEFEAKISDFGTARVLKKSESNWTVPVGSYGYIAPELASTIKVTEKCDVYSFGVVAL
EVILGKHPHELLLCLQSGGYDMLFSYILDKRLAPPTGPIVQELVLAVTLALLCIRENPKSRPTMH
QVSSEL
>VMLRR-RLK77
ILGATGASIVYKAVLADGTAFAVRRIGESGVERFRDFENQVRIIAKLRHPNLVKVRGFYWGNDE
KLVLYDYVSNGSLASSNYKKPGSSPFHLPLEVRFKIARGLARGLAFIHEKKHVHGSIKPSNILLN
FDMEPIISDFGLDRLVLGNNSYKASNSGRNFGSQRSNSTPQEHPITASPYATPSSSSSTTITSPYQA
PESLKSLKPNPKWDVYAFGVILLELLTGRVVSDRELSQWTAGLIVEDKDRVLRLADVAIRVDVE
AKEDAMLACFKLGFSCVSFVPQKRPSMKEAVQVLEKI
>VMLRR-RLK78
LGKSNFSATYKGILRDRSVVVVKCITKTSCKSDEADFLKGLKILTSFKHENLVRLRGFCCSKGR
GECFLIYDFVPNGNLLEYLDVKEGSGRVLEWSTRISIINGIAKGIGYLHGNKGNKSALFHQNISA
EKVLIDRRHNPLLSDSGLHKILADDIIFSMLKASAAMGYLAPEYTTTGRFTEKSDVYAFGMIILQ
ILSGKSNVTPLTRHAVESCKVEDFIDANLERNFPGSEAAKLGRLALLCTHESPNHRPAMETVLQ
ELSGF
>VMLRR-RLK81
IGQGGFGSVYRGILDRDGGKLVAVKVLNLQQHGASKSFIAECKALRNIRHRNLVKILTYCSSIDF
KGNDFKALVFDFMENGSLDTWLYQEGNGNTQVQNLNFLRRLHIAIDVSFALLYLHDDCEAPVI
HCDLKPSNILLDNEMTAHVGDFGLSKLLSKTINNSSQGETSSIGIKGTIGYMAPEYGIGSEASAS
GDVYSLGIILLEMFTGKKPTDEMFTSGLNLHNFVKAKIPGQVMQVVDPKLELGDNNVQKCIVS
ILEIGLACSAEQVGERMNMRDVTRKLNII
>VMLRR-RLK86
NVIGTGSSGVVYKVTIPNGDSLAVKKMWSSEESGAFSSEIQTLGSIRHKNIIRLLGWGSNQNLK
LLFYDYLPNGSLSSLLHGAGKGGAEWETRYDIVLGVAHALAYLHHDCLPAILHGDVKAMNVL
LGPSYEPYLADFGLARVVSSNSNDDLAKPSQRPHLAGSYGYMAPEHASMQRITEKSDVYSFG
VVLLEVLTGRHPLDPTLPGGAPLVQWVSDHLASKKDPVDILDHKLRGRADPTMHEMLQTLAV
SFLCISTRADDRPTMKDIGAMLKEI
>VMLRR-RLK87
ILGQGGFGKVYKGVLQDNTKVAVKRLTDFESPGGDAAFQREVEMISVAVHKNLLRLIGFCTTPS
ERLLVYPFMMNLSVAYRLRERKPEEAVLDWATRKKVALGAARGLEYLHEHCNPKIIHRDVKA
ANVLLDEDFEAVVGDFGLAKLVDVRKTNVTTQVRGTMGHIAPEYLSTGKSSERTDVFGYGIM
LLELVTGQRAIDFSRLEEEDDVLLLDHVKKLEREKRLDAIVDRNLNKNYNILEVEMMIQVALL
CTQASPEDRPAMSDVVRMLEG
>VMLRR-RLK90
LGRGRSGIVYMVTLPSGSTVAVKRFKTMDKFSAAAFSSEIATLARIRHRNIVRLLGWGANRKTK
LLFYDYMSNGTLGALLHEGSIVGLVEWETRFKIALGVAEGLAYLHHDCVPPILHRDVKAHNIL
LGDRYEACLADFGLARLVEDDQGSFSATPQFAGSYGYIAPEYA
>VMLRR-RLK97

IGSGGFGATYKAEIVPGVVVAVKRLSVGRFQGVQQFATEIRTLGRVQHPNLVKLIGYHLSETEM
FLIYNYLPGGNLETFIQERSRRVVEWSMLHKIALDIARALAYLHDECVPRVLHRDIKPSNILLDN
NFNAYLSDFGLARLLGTSETHATTDVAGTFGYVAPEYAMTCRVSDKADVYSYGVVLLELISDK
KALDPSFSSFGNGFNIVAWASMLLRQGRASEFFTAGLWDSGPHNDLIQILHLGIMCTGESLSSRP
SMKQVAQRLKRI
>VMLRR-RLK98
KNILGAGGFGNVYKGKLGDGTMVAVKRLKDVSGNAGESQFRTELEMISLAVHRNLLRLIGYC
ATPNERLLVYPYMSNGSVASRLRGKPALDWNTRKRIAIGAARGLLYLHEQCDPKIIHRDVKAA
NVLLDDFCEAVVGDFGLAKLLNHADSHVTTAVRGTVGHIAPEYLSTGQSSEKTDVFGFGILLIE
LITGMRALEFGKTVNQKGAMLEWVKKILQEKKVEELVDRELRSNYDLIEIGEMLQVALLCTQ
YLPAHRPKMSEV
>VMLRR-RLK99
FSNIIGSFSYGTVYKGTLSSGVEIAVTSTTVISRKEWSKILEAQFRKKIDTLSKMNHKNFVNLIGY
CEENEPFTRIMVFEYAPNGTLFEHLHIKEAEHLDWGMRLRIAMGMAYCLEYMHQLTPPIVHDN
LQSSSIYLTEDYAAKISDFSYWNDVTTKIASIELIKPSSEYPESNVYSFGLILYEMITGRIPYTVEK
CSLTDWTSDILRGQQSLEGMVDPTLKYFKQDELEKLVSVIKNCIHPDAKQRPKMSEVVAKLKEI
>VMLRR-RLK101
LGRGGFGTVYKGTLKSGRQVAVKTLSAQSKQGVREFLNEIDTISKVRHPNLVELLGCCIQETNR
ILVYEYVENNSLDQALLGSKNSCIKLDWKKRSAICLGIARGLTYLHEELVPHIVHRDIKASNILL
DKEFNPKIGDFGLAKLFPDNITHISTRIAGTTGYLAPEYALGGQLTLKADVYSFGVLILEIISGRS
SVKASWGETQKLLLEWAWQLHEEGKHLELVDPELGEFSEEEVIRHMKVAFFCTQGAANRRPL
MSQVVDMLSK
>VMLRR-RLK103
IGQGSFGSVYKGSLVKLEDRLVAIKVLNLEQHGASKTFITECKTLSKIQHRNL
>VMLRR-RLK104
LGKSNFSATYKGILRDRSVVVVKCITKTSCKSDEADFLKGLKILTSFKHENLVRLRGFCCSKGR
GECFLIYDFVPNGNLLEYLDVKEGSGRVLEWSTRISIINGIAKGIGYLHGNKGNKSALFHQNISA
EKVLIDRRYNPLLSDSGLHKILADDIIFSMLKASAAMGYLAPEYTTTGRFTEKSDVYAFGMIILQ
ILSGKSNVTPLTRHAVESCKVEDFIDANLERNFPGSEAAKLGRLALLCTHESPNHRPAMETVLQ
ELSGF
>VMLRR-RLK106
CIGVGAHGNVYKAQLSINKIVALKKLHHSKTENLVNEVEVLTRIRHKNIVKLYGFCLHRRSMFL
IYEYMEKGSLFHALRNDTEAMELNWNIRVKIIKDIAFAVSYLHHGCNPPIVHRDISSKNILLNLE
LKAFVSDFGLARLLDPDSSNQTTMAGTYGYMAPELAYTIVMTEKCDVYSFGVVALELLMGTH
PGEFLSSSLTQNIMLNELLDSRLAPPSNDIDVQDIIFVATIAFSCLCATPKSRPTMEFLSQDFL
>VMLRR-RLK108
ILGATGASIVYKAVLADGTAFAVRRIGESGVERFRDFENQVRIIAKLRHPNLVKVRGFYWGNDE
KLVLYDYVSNGSLASSNYKKPGSSPFHLPLEVRFKIARGLARGLAFIHEKKHVHGSIKPSNILLN
FDMEPIISDFGLDRLVLGNNSYKASNSGRNFGSQRSNSTPQEHPITASPYATPSSSSSTTITSPYQA
PESLKSLKPNPKWDVYAFGVILLELLTGRVVSDRELSQWTAGLIVEDKNRVLRLADVAIRVNVE
AKEDAMLACFKLGFSCVSFVPQKRPSMKEAVQVLEKI
>VMLRR-RLK114
NLIGEGGCSNVYKGCLRKCKPVAIKVLKQYKEAWNDFSFEVDIMSSLKHKNITHLIGVCIEDDH
LILVYDFLSKGSLEERLQVRFKVAIAVAEALNYLHNGCSRSVIHRDVKSSNILLSSDFQPQLSDF

GLAIWGPEDAAYTISSDIVGTFGYIAPEYFMHGRVSDKIDIYSFGIVLLELLTGKKPISSKGLKGQ
EILVMWAMPMLESGNLEALVDPNLGNEFDIVQMHKMVLAATLCIKQSPKLRPKASQILKLL
>VMLRR-RLK118
FSPDNLIGIGSFGSVYKGILDREGIVIAVKVLNLTRHGASKSFAAECETLRNIRHRNLVKVLTACS
GVDYHGNDFKALVYDFMANGSLDGWLHPTIGLDETPKTLNVLQRLNIAIDVACALEYLHYHC
GTPVIHCDLKPSNILLDENMTGHVSDFGLVKFLSNETLDNFINQFSSIGVRGTIGYCPPEYGIGSE
VSTFGDIFSFGILLLEMFTGKRPIDDMFKESLNLHSFVKRALPDQVTQVIDPSL
>VMLRR-RLK122
LGRGGFGVVYKGELDDGTKIAVKRMEAGVISNKAFDEFQAEIAVLSKVRHRHLVSLLGYSIEG
NERILVYEYMPQGALSKHLFHWKSLKLEPLSWKRRLNIALDVARGVEYLHNLAHRSFIHRDLK
SSNILLGDDFRAKVSDFGLVKLAPDGEKSVVTRLAGTFGYLAPEYAVTGKITTKADVFSFGVVL
MELLTGLMALDEDRPEESQYLAAWFWHIKSDKQKLRAAVDPALDVKDETFESISIIAELAGHCT
AREPGQRPDMSHAVNVLAPL
>VMLRR-RLK128
LNEEHVIGFGGFGTVYKLAMDDGNVFALKRILKMNEGFDRFFERELEILGSIKHRYLVNLRGY
CNSPTSKLLIYDYLPGGSLDEALHERSEQLDWDARLNIIMGAAKGLAYLHHDCSPRIIHRDIKSS
NILLDGNLEARVSDFGLAKLLEDEESHITTIVAGTFGYLAPEYMQSGRATEKTDVYSFGVLVLE
VLSGKRPTDASFIEKGLNIVGWLNFLVTENRPREIADPNCEGVQIESLDALLLVATQCVSSSPED
RPTMHRVVQILE
>VMLRR-RLK131
IGQGGFGSVYRGILDRDGGKLVAVKVLNLQQHGASKSFIAECKALRNIRHRNLVKILTYCSSIDF
KALVFDFMENGSLDTWLYQEGNGNTQVQNLNFLRRLHIAIDVSFALLYLHDDCEAPVIHCDLK
PSNILLDNEMTAHVGDFGLSKLLSKTINNSSQGETSSIGIKGTIGYMAPEYGIGSEASASGDVYS
LGIILLEMFTGKKPTDEMFTSGLNLHNFVKAKIPGQVMQVVDPKLELGDNNVQKCIVSILEIGL
ACSAEQVGERMNMRDVTRKLNII
>VMLRR-RLK132
LGRGGFGTVYKGTLKSGRQVAVKTLSAQSKQGVREFLNEIDTISKVRHPNLVELLGCCIQETNR
ILVYEYVENNSLDQALLGSKNSCIKLDWKKRSAICLGIARGLTYLHEELVPHIVHRDIKASNILL
DKEFNPKIGDFGLAKLFPDNITHISTRIAGTTGYLAPEYALGGQLTLKADVYSFGVLILEIISGRS
SVKASWGETQKLLLEWAWQLHEEGKHLELVDPELGEFSEEEVIRHMKVAFFCTQGAANRRPL
MSQVVDMLSK
>VMLRR-RLK133
VLGEGSFGFVFKGWIDEDSLKAAVPGTGMAIAVKKLKPNGCQGQQEWLAEIKYLGHLRHSNL
VKLIGYCIEDNQRLLVYEYMPYSSLDNHLFRRNSHFPPLSWNLCMKVALGAARGLTFLHYEVD
VIYRDFKTSNILLDANYNVKLSDLGLAKDGPIGRRTHVSTRALGTEGYGAPDYIRTGHLTAKSD
VYSFGVVLLEMLCGRQAIDRNKPSEEQNLAKWARNVINARKIFQVLNPAIFGEHSGSSVLKVA
QLAYQCVSAEPKSRPNMKDVVQVLEEL
>VMLRR-RLK134
FEKKIGSGGFGVVYYGKMKDGKEIAVKVLTSNSFQGKREFSNEVTLLSRIHHRNLVQFLGFCQ
DDGRSMLVYEFMHHGTLKEHLYGPLTHGRNINWIKRLEIAEDAAKGIEYLHTGCVPAIIHRDLK
TSNILLDKHMRAKVSDFGLSKLAVDGVSHVSSIVRGTVGYLDPEYYISQQLTDKSDVYSFGVIL
LELMSGQEAISNESFGVNCRNIVQWAKLHIESGDIQGIIDPSLNDEYDIQSMWKIAEKALMCVQ
PHGHMRPSISEVLKEIQ
>VMLRR-RLK135

KNILGAGGFGNVYKGKLGDGTMVAVKRLKDVSGNAGESQFRTELEMISLAVHRNLLRLIGYC
ATPNERLLVYPYMSNGSVASRLRGKPALDWNTRKRIAIGAARGLLYLHEQCDPKIIHRDVKAA
NVLLDDFCEAVVGDFGLAKLLNHADSHVTTAVRGTVGHIAPEYLSTGQSSEKTDVFGFGILLIE
LITGMRALEFGKTVNQKGAMLEWVKKILQEKKVEELVDRELRSNYDLIEIGEMLQVALLCTQ
YLPAHRPKMSEV
>VMLRR-RLK136
FSNIIGSSQDSFVYKGTMKGGSEIAVISLCIKEEHWTGYLELYFQKEVADLARLNSENTGKLLGY
CSESTPFTRMLVFEYASNGTLYEHLHYGEGCQLSWTRRMKIVIGIANGLKYLHTELDPPFTISEL
NSSAVYLTDDFSAKLVDFESWKSILARSEKNSGSIGSQGAICLLPNSIEGRHLDVQGNVYAFGVL
LLEIISGRPPYCKDKGCLVDWAKEYLEMPEVMSYVVDPELKHFKYEDLKVICEVVSLCIHPEPA
KRPSMEEISSILE
>VMLRR-RLK137
QLGEGGYGPVYKGTLSDGRKVAVKLLSLASSQGKSEFVTEIATISAVQHRNLVKLYGCCIEGNR
RLLVYEYLENKSLDKALFGKTSLHLDWPTRFDICLGTARGLAYLHEESRPRIVHRDVKASNILL
DAELCPKISDFGLAKLYDEKKTHISTRVAGTIGYMAPEYAMRGHLTEKADVFSFGVLALEILSGI
PNYENNLVEEKIYLLGWAWTLYEKNQSLALVDPSLMDFDENEALRVLRVALLCTQASPTMRPT
MSRVVAML
>VMLRR-RLK139
LNEEHVIGFGGFGTVYKLAMDDGNVFALKRILKMNEGFDRFFERELEILGSIKHRYLVNLRGY
CNSPTSKLLIYDYLPGGSLDEALHERSEQLDWDARLNIIMGAAKGLAYLHHDCSPRIIHRDIKSS
NILLDGNLEARVSDFGLAKLLEDEESHITTIVAGTFGYLAPEYMQSGRATEKTDVYSFGVLVLE
VLSGKRPTDASFIEKGLNIVGWLNFLVTENRPREIADPNCEGVQIESLDALLLVATQCVSSSPED
RPTMHRVVQILE
>VMLRR-RLK140
IIGQGGFGKVYKGVLPDNTKVAIKRLTDYYSPGGEAAFQREVQLISVAVHRNLLRLIGFCTTSSE
RILVYPYMRNLSVAYQLRELKPGEKGLDWPTRKRVAFGAAHGLEYLHEHCNLKIIHRDLKAAN
ILLDDNFEAVLGDFGLAKLVDTKLTHVTTQIRGTMGHIAPEYLSTGKSSEKTDVFGYGITLLELV
TGQRAIDLSRLEDEEDVLLLDHIKKLLREDRLDDIVDGNLKTYNRQEVKTIIQVALLSTQSSPED
RPTMAEVVKLLQGV
>VMLRR-RLK141
IGKGGFGSVYKAVLSTGQVVAVKKLNISDSSDIPAINLQSFENEIRMLTEVRHRNIIKLYGYCSRR
GCLHLVYEYVERGSLGKVLYGVEGEIDLGWATR
>VMLRR-RLK142
FSNVIGSSPIGTLYKGTLSSGVEIAVAKVAITSSKDWPKNLEVQFRKKIETLSKINHKNFVNLLGY
CEEEEPFTRMLVFEYAPNGTLFEHLHIKESEHLDWGMRLRIAMGMAYCLEHMHQLNPPTTHSN
LNSSAISLTEDYAAKISDFSFSNVVTATEAEATKKFLDTPTARPESNVYSFGVLLFEMVTGRVPY
SVDNDSLEDWASYYLRGDQSLKNMVDPTLDSFEEEKLEKIGEVIKSCVHPDPKQRTEMREVTA
ILREI
>VMLRR-RLK144
IGVGSYGSVYRGILEQVEIEVAVKVINLQQRGASNSFISECQALGSIRHRNLLKLL
>VMLRR-RLK145
IGVGSYGSVYKGFLEQVGIEAAVKVLNLQRRGALKSFVSECQALRTIRHRNLLKLLNVCSSIDF
EGNDFKALIYEFMEKGSLEEWLHAHNAGEDGQEREFGNLKLIDRVNIAIDIANAIEYLHNGSSS
TIVHGDLKP

>VMLRR-RLK147
HFGKESLLSEGRCGPLYRAVLPGDVHVAIKVLENARGIDYEEAVTIFEGLSRLKHPNLLPLCGYC
IAGKEKLVLYEFMANGDLHRWLHELPTLVPNVEDWSTDTWEHQNISGSHIFSPEEKTNWLTRH
RIAIGVARGLAYLHHAGSIHGHLVASNILFSDTLEPRVADFGLRNISPKNKHIVENNGIEFDVYCF
GVVLIELLTGKQGSEETVEWVRRLVREGRGADALVPTLTVSGESVSEMVECLRVGYLCTAELP
GKRPSMQQVLGLLKDI
>VMLRR-RLK148
IGASSLSTVYKGQLEDGQMVAVKKLNLQQFPAESDKSFYREVKTLSQLRHKNLVKVLGYAWE
SRKLKALVLEYMNNGSLESIIHDAHVDQSKWTLSQRIDVWISVASGLEYLHSGYDFPIVHCDLK
PSNILLDSSWVAHVSDFGTARILGVHLQDGSSLSSSSAFQGTIGYLAPEFAYMRKVTTKVDVFSF
GIIIMEFLTKRRPTGLTEEHGIPISLSQLFEKALGNGINGLLQVLDPVIAMNASKDEQTLTELFKL
ALCCTNPNPDDRPNMNEVLSSLNKL
>VMLRR-RLK153
KIGEGGFGSVYKGVLPNGMEIAVKQLSSKSKQGTREFVNEVGTIFALQHPNLVKLLGCCTEDN
QLLLVYEYMENNSLAHALFGSEELRLKLNWPIRFKICLGIAKGLAFLHEESKLKVVHRDIKPTN
VLLDKDLNAKISDFGLAKLYEAEKTHVITRIAGTTGYMAPEYAMRGYLTNKADVYSFGVVALE
IVSGKNGTSYRPNDESVYLLDLAYVLQEKGDLLSLVDPMLGYDYSVKQAMTILDLAMLCTNP
SPTLRPTMSEVVNVLEG
>VMLRR-RLK154
GGFSEANLIKHGHSGDLFRGFLDGGSTIVVKKVNFHSLKKESYMMELELFSKYSHTRLVPLLG
HCSENDNEKLLVYKYMPNADLANSLYRVSEFEDDNLQSLDWITRLKIAIGAAEGLSYLHHECN
PPLVHRDIQASSILLDDKFEVRLGSLSEIRIQEGDSHHNVLTRFLRKPQSSEPGPSGSSSMTCSHD
VYCFGKVLLELITGKLGISKSDDVTAREWLEHALGYISIYDKELVTKIVDPSLIIDEDLLEEVWS
MAIVARSCLNPKPSKRPLMKYILKALEN
>VMLRR-RLK155
IGSGGSGQVYKVKLKSGQIVAVKRLWGGNNKKPETESVFRSEVETLGRVRHGNIVKLLMCCSG
EEFRILVYEYMENGSLGDVLHGEKDGGLMDWTERFAVAVGAAQGLAYLHHDCVPPIVHRDVK
SNNILLDEEMRPKVADFGLAKTLQSDVGEGDAVMSRIAGSYGYIAPEYAYTLK
>VMLRR-RLK156
KVGEGGFGIVYKGVLKDGTVAAIKVLSPESRQGVREFLTEIKLIADVEHDNLVKLYGCCVEEDH
RILVYGYLKNNSLAQTLLGGNRSSIQFNWPTRCRICIGVAQGLAFLHEEVRPHIVHRDIKASNIL
LDENLMPKISDFGLAKLFPPNLTHISTRVAGTAGYLAPEYAIRGQLTRKADIYSFGVLLLEIVCGR
SNTNRRLPPEEQYLLERVWDSHEKGELVSLVDASLNEDYDTEEACKYLKIGLLCTQDMPKLRP
TMSTVVKMLMG
>VMLRR-RLK159
IGIGHFSSVYKGKLKDGKEVAIKVFNVEEETSLRSFDLECEVFSRVSHPNLVRTVCISNSINFKAL
VMEYMANGSLEKWLHTHKYDLDILQRVDIMIETAKAVRYLHQRCIIHCDIKPSNIILDEDMVAH
VSDFSIAKIQPVSRIRTIQSKLMCTIGYVAPEYGRYGIVSASMDVYSFGILLMETFTGKKPTHEM
FTGEMNLRRWVIESLPCEVERVIDPSLLQTEEENNDVYMKCISSVMRLALICSAESPSERLNMR
DVEEKLNNI
>VMLRR-RLK160
VGHGGSGTVYKIELSNGEVVAVKRLWSKRTKDSSSEDQLLLDKNLKAEVGTLGSIRHKNIVKL
YCYFSSLNCSLLVYEYMSNGNLWDALHKNKIYLDWPTRHQIALGVAQGLAYLHHDLLSPIIHR
DIKSTNILLDVNYQPKVADFGIAKVLQARAGKDSTTTIVAGTYGYMAPEYAYSSKATTKCDVY

SFGIVLMELITGKKPIHADFGENKNIVYWVSAKVDTKEGVMEVLDKRLSGSFRDEMIQVLRIAI
RCTCNTPALRPTMNEVVQLLID
>VMLRR-RLK161
LDEEDVVGSGGFGTVYRMVMNDCGTFAVKRIDRSREGSDQVFERELEILGSIKHINLVNLRGY
CRLPMAKLLIYDYLAMGSLDDILHEHVQEQPLNWSARLSIALGSARGLAYLHHDCSPKIVHRD
IKSSNILLDETLEPHVSDFGLAKLLVDEDAHVTTVVAGTFGYLAPEYLQSGRATEKSDVYSFGV
LLLELVTGKRPTDPAFVKRGLNVVGWLNTLLRENRLDDVVDKRCKDADMETVEAILEIAARC
TDANPDDRPTMNQVLQLLE
>VMLRR-RLK163
KTRFSTYYKATMPSGASYFVKKLNWSDKIFQLGSHDKFDQELEVLGKLSNSNVMTPLAYVLT
VDSAYLFYEHAQKGTLFDVLHGKLGNALDWGSRYSIAVGVAQGLTFLHGYTSGPILLLDLSSR
NILLKSLKEPLVGDIELYKVIDPTKSTGSLSTVAGSVGYIPPEYAYTMRVTMAGNVYSFGVVLLE
LLTGKPAVSEGTELAKWVSSNSAQKDRWDHILDFSISRTSLAVRGQMLAMLKVALSCVSLSPEA
RPKMKSVLRMI
>VMLRR-RLK165
LIGSGQFGHVYKGVLQDNTRIAVKVLASKTTCGEISGSFKRECQVLKRARHRNLIKIITICSKPD
FKALVLPLMANGSLERYLYPSHGFLLNPGLDLIQLVSICCDVAEGVAYLHHYSPVRVVHCDLKP
SNILLDDDMTALVTDFGIARLVKGIDDNDQNSTSTNNNNDSVSFSSTDGLLCGSVGYIAPEYGM
GRRASTQGDVYSFGVLLLEILVGKRPTDVLFHEGSSLHEWVKSHYPRKLETIVEQTMVRWCA
QISSMPSAYYIYDNKIWSDVIVELIELGLMCTQYNPSTRPTMLDVAHEMGRL
>VMLRR-RLK166
FKEVIGRGSFGSVYLGKLSDGKPVAVKVRFDKTQLGADSFINEVYLLSQIRHQNLVCLEGFCYE
SKQQILVYEYLPGGSLAEHLYGLNSQKVSLSWVRRLKIAVDAAKGLDYLHNGNEPRIIHRDVK
CSNILLDKDMNAKVCDFGLSKQVMQADASHVTTVVKGTAGYLDPEYYSTQQLTEKSDVYSF
GVVLLELICGREPLRHSGTPDSFNLVLWTTIVKMQAKPYLQAGAFEIVDDSIKGTFDVESMRKA
ATVAVRSVERDASQRPNIAEVLAELKE
>VMLRR-RLK167
IGNGGFGLVYKGCLEDGSAVAIKVIDITQTGFQKSFLAECEALRNVRHRNLVKLITSCSSVDLKN
TEFLALVYEFLGNGSLQDWIQGKRKNEDGDGLNLVERLNVAIDIASAIDYLHNDCEVPVVHCD
LKPNNILLDEDMTAKVGDFGLAKLLIEKMSDQTSISSTYALKGSIGYIPPEYGLGVKPSTAGDV
YSFGVMLLELFTGKSPTGDSFMVGQNLVGWVQSAFPADIEQVLDPELQSLMDNFDSDSQTIKP
EAQHYCLITILGIGLACTVASPDNRISIRNTLSKLQ
>VMLRR-RLK168
GGFSEANLIKHGHSGDLFRGFLDGGSTIVVKKVNFHSLKKESYMMELELFSKYSHTRLVPLLG
HCSENDNEKLLVYKYMPNADLANSLYRVSEFEDDNLQSLDWITRLKIAIGAAEGLSYLHHECN
PPLVHRDIQASSILLDDKFEVRLGSLSEIRIQEGDSHHNVLTRFLRKPQSSEPGPSGSSSMTCSHD
VYCFGKVLLELITGKLGISKSDDVTAREWLEHALGYISIYDKELVTKIVDPSLIIDEDLLEEVWS
MAIVARSCLNPKPSKRPLMKYILKALEN
>VMLRR-RLK170
IGKGHIGIVYKAGMPFNVTFAVKKISPQTKQQEKDKIQREIFNLLSLRHENLVQLLGSCSRKGH
HILIYEYMENGSLHQALFEPDSTIELDWKARYDICLGIAKGLKYLHEDKRFEIVHGNITARNILL
DKNHTPKISDFGLARFRDDEDAFTTIKTRERLYVAPEYFLGKAITVKADVYSYGVVVLEIVSGR
TSMEQRPNQEYDVLLDAACVLHARGKILDLVDKKLSSSYDRKQALILLDTAIKCINSQSPTLRP
KMSDVFSAL

>VMLRR-RLK172
VGEGGFGSVYKGLLSDGTVIAVKQLSSKSKQGNREFVNEIGMISALQHPNLVKLLGCCTEDNQ
LLLVYEYMENNSLAHALFGSEELRLKLNWPIRFKICLGIAKGLAFLHEESKLKVVHRDIKPTNV
LLDKDLNAKISDFGLAKLYEAEKTHVITRIAGTTGYMAPEYAMRGYLTNKADVYSFGVVALEI
VSGKNGTSYRPNDESVYLLDLAYVLQEKGDLLSLVDPMLGYDYSVKQAMTILDLAMLCTNPS
PTLRPTMSEVVNVLEG
>VMLRR-RLK174
EVRILGALQHSCIVELYGHQISSKWVPSEEGKPERQILQSAILMEYVKGGSLKSYIEKASKTGEK
HVPVELALCIARDVAYALAELHSKHIIHRDVKSENILIDVENKRANGMPVVKLCDFDRAVPLRS
FLHTCCITHRGVPPPDVCVGTPRWMAPEVLQAMHKRSLYGLEVDIWSYGCLLLELLTLQVPYS
GLSEFHINELLQTGKRPPLTKELETLASMNEPATTQPGSNLAGAEAESETLRFLVDLFHRCTEGN
PANRPTAAEIYELL
>VMLRR-RLK175
IGNGGFGATYKAEISSGVLVAIKRLAVGRFQGVQQFHAEIKTLGRLHHPNLVTLIGYHASETEM
FLIYNYLPG
>VMLRR-RLK176
VGQGGYGKVYKGILADGTVVAIKRAQEHSLQGEKEFLTEIELLSRLHHRNLVSLTGYCDEEGE
QMLVYEFMPNGTLRDHLSAKSKVPLSFATRLRTALGSAKGILYLHTEADPPIFHRDIKASNILLD
SNFNAKVADFGLSRLAPVPDDEGAAPAHVSTVVKGTPGYLDPEYFLTHKLTDKSDVYSLGVVF
LELLTGMQAISHGKNIVREVNVSYQSGMIFSVIDGRMGSYPSDCVEKFLNLAMKCCKDETDAR
PSMADVVRELETI
>VMLRR-RLK179
IGKGSFGTVYYGQMKDGKEIAVKIMADSSSHLRQQFVTEVALLSRIHHRNLVPLIGFCEEEHQRI
LVYEYMHNGTLRDHIHGLRTI
>VMLRR-RLK184
LGAGGFGAVYKGTLKDGTIIAVKQLSSRSKQGNREFLNEIGMISVLQHPNLVRLYGCCVERNQL
LLVYEYMENNSLAHALFDTEESQSKMDWATRQRICVGIAKGLAFLHEESALKIVHRDIKTANIL
LDGDLNPKISDF
>VMLRR-RLK187
IGNGGFGATYKAEISPGTLVAVKKLALGRFQGVQQFHAEIKALGRVRHPNLVTLIGYHASEAEM
FLIYNYLPGGNLE
>VMLRR-RLK192
IGVGGYGIVYKVVLPTGRVVAVKKLHQSQNGEMDDFKAFKSEICVLMNIRHRNIVKLYGFCSH
VKHSFLVYEFIERGSLRNVLSNEEQAMELDWFKRLNVVKGIANALAYMHHDCCPPIIHRDISSN
NVLLDSEFEAHVSDFGTARLLMPDSSNWTSFAGTFGYTAPELAYTMAVNEKCDVYSFGVVTLE
IIMGRHPGDFISTLSLSSSSLSWPTDQHTLEDVIDQRLPFPRNKAANGLILNIKLALACLSANPQS
RPTMRQVSSQL
>VMLRR-RLK194
IGVGKFGEVYKGLLNDGMLVAIKKRPGAPSQEFVDEVRYLSPIQHRYLVTLLGFCQENNQQFLI
YEYIPSGSVSSHLYGPSQVSDGKLEFKHRLSIALGAAKGDPLQNQFGSSSLFESPFAKIDIMSSL
MKVQNTQDYSVFSSIVDPRLGTSFTAEGMEEFIQLIVRCLEPSSERRPSMSYVVMELDRI
>VMLRR-RLK195
HGHSGDLFRGTLENGISVVIKRVDMQSIKKETYLLELDFFSKVSHPRLVPLLGHCLENENEKFLI
YKYMPNGDLSSSLYRKTSEEDGSLQSLDWITRLKIATGAAEGLSCLHHECTPPIVHRDVQASSIL

LDDKFEVRLGSLSEACPQEGDTHQSRITRLLRLPQSSEQGASGSVTTTCAYDVYCFGKVLLELV
TGKLGMSASSEAQLKEWLEQTLPYISMYEKELVTKIVDPSLIIDEDLLEEVWAMAIVARSCLNP
KPSRRPPMRYILKALEN
>VMLRR-RLK197
IGVGGYGIVYKVVLPTGRVVAVKKLHQSQNGEMDDFKAFKSEICVLMNIRHRNIVKLYGFCSH
VKHSFLVYEFIERGSLRNVLSNEEQAMELDWFKRLNVVKGIANALAYMHHDCCPPIIHRDISSN
NVLLDSEFEAHVSDFGTARLLMPDSSNWTSFAGTFGYTAPELAYTMAVNEKCDVYSFGVVTLE
IIMGRHPGDFISTLSLSSSSLSWPTDQHTLEDVIDQRLPFPRNKAANGLILNIKLALACLSANPQS
RPTMRQVSSQL
>VMLRR-RLK198
FNEINLLGRGSFGSVYKGTLQGGMNVAVKVFNLQLEEGFKSFDVECEVLCRIRHRNLVKIITAC
CNIDFKALVLEFMPNWSLEKWLYSHNYFLDILQRLNIMVDVALALEYLHYGYTTPVVHCDLKP
SNILLDEDMVGHVSDFGIAKLLGEEHSIIQTMTLATVGYMAPEYGLEGLVSIRGDMYSYGILM
METFTRKKPTDEMFSEEMNMKQWVKESFSLPCGVTEIADANLLKSDGHFSAKKDCIASIMAL
ALKCSADLPEERIDIKDAL
>VMLRR-RLK202
EIGRGRFGIVYKAEMPDQTKLAVKKISPESTQDKLKDELQGEIFFNVKSLEHENLIQLFDGYSK
KDLHLLIYEYMENGSLDQALFEPKSRIELNWEVRFNICLGLAKALKYLHEENVRKIIHRNIKAS
NILLDENYTAKLTDFGLASFDNEDDPFKTIKAGGARAYFAPEYARGQAITDKADVYSFGVVTLE
IFSGRSCTENKSEAQKYLLDDACIFQARGKIDQLVDKKLANYDRKLVPKILNLAIMCINPTADL
RPTMSEVVSVL
>VMLRR-RLK203
IGVGKFGEVYKGLLNDGMLVAIKKRPGAPSQEFVDEDFKTANVLVDENFIAKVADAGLRNFLG
RVDVAAKIDIMSSLMKVQNTQDYSVFSSIVDPRLGTSFTAEGMEEFIQLIVRCLEPSSERRPSMS
YVVMELDRI
>VMLRR-RLK204
LGAGGFGAVYKGTLKDGTIIAVKQLSSRSKQGNREFLNEIGMISVLQHPNLVRLYGCCVERNQL
LLVYEYMENNSLAHALFDTEESQSKMDWATRQRICVGIAKGLAFLHEESALKIVHRDIKTANIL
LDGDLNPKISDF
>VMLRR-RLK205
IGVGSYGSVYRGILEQVEIEVAVKVINLQQRGASNSFISECQALGSIRHRNLLKLLSVCSSMDFE
GNDFKALIYEFMANGSLEKWLHVHGQERNSTNLKLINRLNIAIDIAFAIEYLHNGSSSTIIHGDL
KPSNVLLDEEMTAHIGDFGLAKIVSTISGEAMQFPSTSFAIKGSIGYVAPEYGMGEPVSIEGDVY
SYGILLLEMFTGKKPTDDYFKDELNLHTLVERSLPHKAMEIVDSSILSEDARGSFKEYCVISVLR
IGVACSMELPAERIKMRDVISELEKI
>VMLRR-RLK207
FSSKMEIGRGRFGIVYKAEMPDQTKLAVKKISPESTQDKLKDELQGEIFFNVKSLEHENLIQLFD
GYSKKDLHLLIYEYMENGSLDQALFEPKSRIELNWEVRFNICLGLAKALKYLHEENVRKIIHRN
IKASNILLDENYTAKLTDFGLASFDNEDDPFKTIKAGGARAYFAPEYARGQAITDKADVYSFGV
VTLEIFSGRSCTENKSEAQKYLLDDACIFQARGKIDQLVDKKLANYDRKLVPKILNLAIMCINPT
ADLRPTMSEVVSVL
>VMLRR-RLK208
IGEGGFGPVYKGLLPDGTVIAVKQLSSKSSQGNREFLNEIGMISCLQHPNLVKLHGCCVEGNQL
LLVYEYMENNSLARALLGPEHSRIKLDWQTRQRICVGIARGLAFLHEESRLKIVHRDIKATNVL

LDKHLNPKISDFGLAKLDTEEKTHISTRIAGTMGYMAPEYALWGYLTHKVDVYSFGIVALEIVS
GKHNMSHGPENNFACLLDWACHLQQSGNLMELVDEKLGSDFKKKEAERMIKASLLCTNGSPS
LRPSMSEVVNMLEG
>VMLRR-RLK209
IGEGGFGPVFKGLLSDGTIIAVKQLSSKSKQGNREFVNEIGMISALQHPHLVKLYGCCIEGNQLL
LVYEYMENNSLARALFGPEEHQLNLDWSTRHKICVGIARGLAFLHEESRLKIVHRDIKATNVLL
DKYLNSKISDFGLAKLDEEENTHISTRVAGTFGYMAPEYAMRGYLTDKADVYSFGIVALEIVSG
RCNTSNRSNMKEECFYLLDWACVLKEKGNLLELVDPKLGKNYNKEQVMTMINVALLCTNVS
PVVRPAMSSVVSMLEG
>VMLRR-RLK210
IGEGGFGPVYKGLLPDGTVIAVKQLSSKSSQGNREFLNEIGMISCLQHPNLVKLHGCCVEGNQL
LLVYEYMENNSLARALLGPEHSRIKLDWQTRQRICVGIARGLAFLHEESRLKIVHRDIKATNVL
LDKHLNPKISDFGLAKLDTEEKTHISTRIAGTMGYMAPEYALWGYLTHKVDVYSFGIVALEIVS
GKHNMSHGPENNFACLLDWACHLQQSGNLMELVDEKLGSDFKKKEAERMIKASLLCTNGSPS
LRPSMSEVVNMLEG
>VMLRR-RLK212
LGRGSFGSVYKGTLQGGMNVAVKVFNLQLEGGFKSFDVECEVLQRIRHRNLVKIITSCSSIDFR
ALVLEFMPNWSLEKWLYSHNYFLDIMQRLNIMIDVALALEYLHYGSTTPVVHCDLKPSNILLD
EDMVGHVSDFGIAKLLGEEHSIIQTMTLATVGYMAPEYGSEGLISIRGDMYSYGILVMETFTRK
RPIDEMFTEEMSMKQWVRDSLSLPCGVTEIADSNLLKRNEQHFSAKKDCIESILILAQKCCADL
PDERIDIKDVFSTLKKI
>VMLRR-RLK213
IGNGGFGATYKAEISSGVLVAIKRLAVGRFQGVQQFHAEIKTLGRLHHPNLVTLIGYHASETEM
FLIYNYLPGGNLEKFIQGRSTRAVDWRILHKIALDIARALAYLHDQCVPRVLHRDVKPSNILLD
DDFNAYLSDFGLARLLGTSETHATTGVAGTFGYVAPEYAMTCRVSDKADVYSYGVVLLELLSD
KKALDPSFSSYGNGFNIVAWGCMLLRQGRAKEFFTAGLWDAGPHDDLVEVLHLAIVCTVDSLS
TRPTMKQVVRRLKQL
>VMLRR-RLK215
IGEGGFGPVYKGLLSDGTIIAVKQLSSKSKQGNREFVNEIGMISALQHPHLVKLYGCCIEGNQLL
LVYEYMENNSLARALFGKSNKHNLFLHFHDACTSEKYIIIAVFLCSLLIGDLKGYEEHQLNLDW
STRHKICVGIARGLAFLHEESRLKIVHRDIKATNVLLDKYLNSKISDFGLAKLDEEENTHISTRV
AGTFGYMAPEYAMRGYLTDKADVYSFGIVALEIVSGRCNTSNRSNMKEECFYLLDWACVLKE
KGNLLELVDPKLGKNYNKEQVMTMINVALLCTNVSPVVRPAMSSVVSMLEG
>VMLRR-RLK216
IGKDGRFGIIYKAELSNLTVAVKKLFPQSKAVAQIGTEVYAKTFKLEHDNLVQLLATYSRKDLHL
LIYEYMDRGSLEQALFDPKPSMQLSWKTRYSICLQIAQGLEYLHAKNPPIIHRDIKASNILLDGS
SKAKISDFGLAKLYEEDNPFMFIEEGGTRMYMAPEYATHKAVTVGVDVFSFGVLLLEIVTGIKN
DENLSKHHDQTIFLLEKVANLHAMVKDAKLQYKKVKYAELVDEKLLNSNYSEEEVVTIIDLA
MLCTDQMASLRPIISDVVSVLEG
>VMLRR-RLK218
IGKGGYGTVYKANLPSGSTVAVKKLHPLGNGERPYQKEFLNEIRALTEIRHRNIVRLHGFCSYA
SHSFLVYEYLEGGSLDAVLGNDKEAKELDWSKRVNIVKGVAQALSYMHHDCSPPIVHRDIKSK
NILLDSEYEAHISDFGTAKLLNLDSSHWTALAGTYGYVAPEFAYTMK
>VMLRR-RLK220

IGKDGRFGIIYKAELSNLTVAVKKLFPQSKAVAQIGTEVYARTFKLVHDNLVQLLATYSRKDLHL
LIYEYMDRGSLEQALFDPKPSMQLSWKTRYSICLQIAQGLEYLHAKNPPIIHRDIKASNILLDGS
SKAKISDFGLAKLYEEDNPFMFIEEGGTRMYMAPEYATHKAVTVGVDVFSFGVLLLEIVTGIKN
DENLSKHHDQTIFLLEKVANLHAMVKDAKLQYKKVKYAELVDEKLLNSNYSEEEVVTIIDLA
MLCTDQMASLRPIISDVVSVLEG
>VMLRR-RLK221
KLGEGGFGPVYKGILNDGRVIAVKQLSVASHQGKSQFVTEIATISAVQHRNLVKLYGCCIEGYN
RLLVYEYLENKSLDQALFGETSLNLDWPTRYQICLGVARGLAYLHEESRLRIVHRDVKASNILL
DSELIPKISDFGLAKLYDDKKTHISTRVAGTIGYLAPEYAMRGHLTEKADVFAFGVVVLEIISGRP
NSDSSLEEEKIYLLEWAWNLHENNREVELVDSKLSDFSEEDVKRLIGVALLCTQTSPNLRPSMS
RVIAML
>VMLRR-RLK222
KLGEGGFGPVYKGILNDGRVIAVKQLSVASHQGKSQFVTEIATISAVQHRNLVKLYGCCIEGYN
RLLVYEYLENKSLDQALFGETSLNLDWPTRYQICLGVARGLAYLHEESRLRIVHRDVKASNILL
DSELIPKISDFGLAKLYDDKKTHISTRVAGTIGYLAPEYAMRGHLTEKADVFAFGVVVLEIISGRP
NSDSSLEEEKIYLLEWAWNLHENNREVELVDSKLSDFNEQDVKSLIGVALLCTQTSPNLRPSMS
RVIAML
>VMLRR-RLK225
FSTLNLIGVGTFGSVYKGTLEEDGVIIAVKVLNLQQQGAAKSFMAECKVLQNIRHKNLLRIITS
CSSIDFQGNDFKALIYEYMPNGNLEKWLHPSSEIYAEPIEQQNLNLLQRMNIAIDVANALTYLH
HSCQKPIIHCDLKPSNILLDNDMVAHIGDFGLAKFLSHFPNPVQSSSI
>VMLRR-RLK229
IGSGGSGTIYRAELHTGETVAVKRILWKDDFLLNKSFTREVKTLGRIRHRHLVKLLGYCTNKGA
GSNLLIYEYMENGSVWDWLHQQPVNSKIKKSLDWEARLRIAVGLAQGVEYLHHDCVPTLIHR
DIKSSNVLLDSSMEAHLGDFGLAKALVEDYDSYTESNSWFAGSY
>VMLRR-RLK237
IGSGGSGTIYRAELHTGETVAVKRILWKDDFLLNKSFTREVKTLGRIRHRHLVKLLGYCTNKGA
GSNLLIYEYMENGSVWDWLHQQPVNSKIKKSLDWEARLRIAVGLAQGVEYLHHDCVPTLIHR
DIKSSNVLLDSSMEAHLGDFGLAKALVEDYDSYTES
>VMLRR-RLK252
RLPDGLMDENMFRKEAEFLSKVKHRNLTVLRGYYAGQQQDLRLVVYDYMPNGNLATLLQEA
SHQDGHVLNWPMRHLIALGIARGLAFLHTSNMVHGDVKPQNVLFDADFEAHLSDFGLDRLTI
ATPAEASSSTTVGTLGYVSPEAILTGEVSKESDVYSFGIVLLELLTGKRPVMFNEDEDIVKWVK
KQLQRGQITELLEPGLLELDPESSEWEEFLLGVKVGLLCTAPDPLDRPTMSDIVFMLEG

Amino acid sequences of RLK domain from *A. thaliana*

>At4g29990
FERVLGKGGFGKVYHGFLNGDQVAVKILSEESTQGYKEFRAEVELLMRVHHTNLTSLIGYCNE
DNHMALIYEYMANGNLGDYLSGKSSLILSWEERLQISLDAAQGLEYLHYGCKPPIVHRDVKPA
NILLNENLQAKIADFGLSRSFPVEGSSQVSTVVAGTIGYLDPEYYATRQMNEKSDVYSFGVVLL
EVITGKPAIWHSRTESVHLSDQVGSMLANGDIKGIVDQRLGDRFEVGSAWKITELALACASESS
EQRPTMSQVVMELKQ
>At4g33430

SNKNILGRGGFGKVYKGRLADGTLVAVKRLKEERTQGGELQFQTEVEMISMAVHRNLLRLRGF
CMTPTERLLVYPYMANGSVASCLRERPESQPPLDWPKRQRIALGSARGLAYLHDHCDPKIIHR
DVKAANILLDEEFEAVVGDFGLAKLMDYKDTHVTTAVRGTIGHIAPEYLSTGKSSEKTDVFGY
GVMLLELITGQRAFDLARLANDDDVMLLDWVKGLLKEKKLEALVDVDLQGNYKDEEVEQLI
QVALLCTQSSPMERPKMSEVVRMLEGD
>At2g13790
SNKNVLGRGGFGKVYKGRLADGNLVAVKRLKEERTKGGELQFQTEVEMISMAVHRNLLRLRG
FCMTPTERLLVYPYMANGSVASCLRERPEGNPALDWPKRKHIALGSARGLAYLHDHCDQKIIH
RDVKAANILLDEEFEAVVGDFGLAKLMNYNDSHVTTAVRGTIGHIAPEYLSTGKSSEKTDVFG
YGVMLLELITGQKAFDLARLANDDDIMLLDWVKEVLKEKKLESLVDAELEGKYVETEVEQLI
QMALLCTQSSAMERPKMSEVVRMLEGD
>At1g71830
SNKNILGRGGFGKVYKGRLADGTLVAVKRLKEERTPGGELQFQTEVEMISMAVHRNLLRLRGF
CMTPTERLLVYPYMANGSVASCLRERPPSQPPLDWPTRKRIALGSARGLSYLHDHCDPKIIHRD
VKAANILLDEEFEAVVGDFGLAKLMDYKDTHVTTAVRGTIGHIAPEYLSTGKSSEKTDVFGYGI
MLLELITGQRAFDLARLANDDDVMLLDWVKGLLKEKKLEMLVDPDLQTNYEERELEQVIQV
ALLCTQGSPMERPKMSEVVRMLEGD
>At1g34210
SNKNILGRGGFGKVYKGRLADGTLVAVKRLKEERTPGGELQFQTEVEMISMAVHRNLLRLRGF
CMTPTERLLVYPYMANGSVASCLRERPPSQLPLAWSIRQQIALGSARGLSYLHDHCDPKIIHRD
VKAANILLDEEFEAVVGDFGLARLMDYKDTHVTTAVRGTIGHIAPEYLSTGKSSEKTDVFGYGI
MLLELITGQRAFDLARLANDDDVMLLDWVKGLLKEKKLEMLVDPDLQSNYTEAEVEQLIQV
ALLCTQSSPMERPKMSEVVRMLEGD
>At5g16000
MESTIVMMMMITRSFFCFLGFLCLLCSSVHGLLSPKGVNFEVQALMDIKASLHDPHGVLDNW
DRDAVDPCSWTMVTCSSENFVIGLGTPSQNLSGTLSPSITNLTNLRIVLLQNNNIKGKIPAEIGRL
TRLETLDLSDNFFHGEIPFSVGYLQSLQYLRLNNNSLSGVFPLSLSNMTQLAFLDLSYNNLSGP
VPRFAAKTFSIVGNPLICPTGTEPDCNGTTLIPMSMNLNQTGVPLYAGGSRNHKMAIAVGSSVG
TVSLIFIAVGLFLWWRQRHNQNTFFDVKDGNHHEEVSLGNLRRFGFRELQIATNNFSSKNL
>At3g25560
VGKGGFGNVYKGCLHDGSIIAVKRLKDINNGGGEVQFQTELEMISLAVHRNLLRLYGFCTTSSE
RLLVYPYMSNGSVASRLKAKPVLDWGTRKRIALGAGRGLLYLHEQCDPKIIHRDVKAANILLD
DYFEAVVGDFGLAKLLDHEESHVTTAVRGTVGHIAPEYLSTGQSSEKTDVFGFGILLLELITGLR
ALEFGKAANQRGAILDWVKKLQQEKKLEQIVDKDLKSNYDRIEVEEMVQVALLCTQYLPIHR
PKMSEVVRMLEGD
>At1g60800
KNILGRGGYGIVYKGHLNDGTLVAVKRLKDCNIAGGEVQFQTEVETISLALHRNLLRLRGFCSS
NQERILVYPYMPNGSVASRLKDNIRGEPALDWSRRKKIAVGTARGLVYLHEQCDPKIIHRDVKA
ANILLDEDFEAVVGDFGLAKLLDHRDSHVTTAVRGTVGHIAPEYLSTGQSSEKTDVFGFGILLL
ELITGQKALDFGRSAHQKGVMLDWVKKLHQEGKLKQLIDKDLNDKFDRVELEEIVQVALLCT
QFNPSHRPKMSEVMKMLEGD
>At3g13065
MNSPSKLKGWSSSGGDPCGDSWDGITCKGSSVTEIKVSGRGLSGSLGYQLGNLKSLTYLDVSK
NNLNGNLPYQLPDKLTYLDGSENDFNGNVPYSVSLMNDLSYLNLGRNNLNGELSDMFQKLPK

LETIDLSSNQLTGKLPQSFANLTGLKTLHLQENQFKGSINALRDLPQIDDVNVANNQFTGWIPNE
LKNIGNLETGGNKWSSGRAPSPPPGTRHIDRNSSGGGGGSSKALTLGVIIAVSSIGGLILFAGLIA
LISRRKNSNDSSHFFDDEKGTNRSKPLFTPQSSQMLQFDNMEEFKNQKTVDSNTSLETKPSVKR
TSSVSFKNSPTFHLIPSTQVAATPDRSSTSQDSPDTRGVKAFSLADLQNTASCFSP
>At1g11130
IIGEGSIGNVYRAELRHGKFLAVKKLSNTINRTQSDGEFLNLVSNVLKLKRGHILELLGYCNEFG
QRLLVYEYCPNGSLQDALHLDRKLHKKLTWNVRINIALGASKALQFLHEVCQPPVVHQNFKS
SKVLLDGKLSVRVADSGLAYMLPPRPTSQMAGYAAPEVEYGSYTCQSDVFSLGVVMLELLTG
RRPFDRTRPRGHQTLAQWAIPRLHDIDALTRMVDPSLHGAYPMKSLSRFADIISRSLQMEPGFRP
PISEIVQDLQ
>At4g39400
IGSGGFGDVYKAILKDGSAVAIKKLIHVSGQGDREFMAEMETIGKIKHRNLVPLLGYCKVGDE
RLLVYEFMKYGSLEDVLHDPKKAGVKLNWSTRRKIAIGSARGLAFLHHNCSPHIIHRDMKSSN
VLLDENLEARVSDFGMARLMSAMDTHLSVSTLAGTPGYVPPEYYQSFRCSTKGDVYSYGVVL
LELLTGKRPTDSPDFGDNNLVGWVKQHAKLRISDVFDPELMKEDPALEIELLQHLKVAVACLD
DRAWRRPTMVQVMAMFKEI
>At1g55610
VGSGGFGEVYKAQLRDGSVVAIKKLIRITGQGDREFMAEMETIGKIKHRNLVPLLGYCKVGEE
RLLVYEYMKWGSLETVLHEKSSKKGGIYLNWAARKKIAIGAARGLAFLHHSCIPHIIHRDMKS
SNVLLDEDFEARVSDFGMARLVSALDTHLSVSTLAGTPGYVPPEYYQSFRCTAKGDVYSYGVI
LLELLSGKKPIDPGEFGEDNNLVGWAKQLYREKRGAEILDPELVTDKSGDVELFHYLKIASQCL
DDRPFKRPTMIQLMAMFKE
>At2g01950
IGHGGFGEVFKATLKDGSSVAIKKLIRLSCQGDREFMAEMETLGKIKHRNLVPLLGYCKIGEER
LLVYEFMQYGSLEEVLHGPRTGEKRRILGWEERKKIAKGAAKGLCFLHHNCIPHIIHRDMKSS
NVLLDQDMEARVSDFGMARLISALDTHLSVSTLAGTPGYVPPEYYQSFRCTAKGDVYSIGVV
MLEILSGKRPTDKEEFGDTNLVGWSKMKAREGKHMEVIDEDLLKEGSSESLNEKEGFEGGVIV
KEMLRYLEIALRCVDDFPSKRPNMLQVVASLREL
>At3g13380
IGSGGFGDVYKAKLADGSVVAIKKLIQVTGQGDREFMAEMETIGKIKHRNLVPLLGYCKIGEE
RLLVYEYMKYGSLETVLHEKTKKGGIFLDWSARKKIAIGAARGLAFLHHSCIPHIIHRDMKSSN
VLLDQDFVARVSDFGMARLVSALDTHLSVSTLAGTPGYVPPEYYQSFRCTAKGDVYSYGVILL
ELLSGKKPIDPEEFGEDNNLVGWAKQLYREKRGAEILDPELVTDKSGDVELLHYLKIASQCLDD
RPFKRPTMIQVMTMFKE
>At1G69270
IGHGGFGSTYKAEVSPTNVFAVKRLSVGRFQGDQQFHAEISALEMVRHPNLVMLIGYHASETE
MFLIYNYLSGGNLQDFIKERSKAAIEWKVLHKIALDVARALSYLHEQCSPKVLHRDIKPSNILL
DNNYNAYLSDFGLSKLLGTSQSHVTTGVAGTFGYVAPEYAMTCRVSEKADVYSYGIVLLELIS
DKRALDPSFSSHENGFNIVSWAHMMLSQGKAKEVFTTGLWETGPPDDLVEVLHLALKCTVDS
LSIRPTMKQAVRLLKRI
>At3g02130
IGNGGFGATYKAEISQDVVVAIKRLSIGRFQGVQQFHAEIKTLGRLRHPNLVTLIGYHASETEMF
LVYNYLPGGNLEKFIQERSTRDWRVLHKIALDIARALAYLHDQCVPRVLHRDVKPSNILLDDD
CNAYLSDFGLARLLGTSETHATTGVAGTFGYVAPEYAMTCRVSDKADVYSYGVVLLELLSDK

KALDPSFVSYGNGFNIVQWACMLLRQGRAKEFFTAGLWDAGPHDDLVEVLHLAVVCTVDSLS
TRPTMKQVVRRLKQL
>At5g07280
IGDGGFGTVYKACLPGEKTVAVKKLSEAKTQGNREFMAEMETLGKVKHPNLVSLLGYCSFSE
EKLLVYEYMVNGSLDHWLRNQTGMLEVLDWSKRLKIAVGAARGLAFLHHGFIPHIIHRDIKAS
NILLDGDFEPKVADFGLARLISACESHVSTVIAGTFGYIPPEYGQSARATTKGDVYSFGVILLEL
VTGKEPTGPDFKESEGGNLVGWAIQKINQGKAVDVIDPLLVSVALKNSQLRLLQIAMLCLAETP
AKRPNMLDVLKALKEI
>At5g48380
IATGRTGTMYKGRLEDGSLLMIKRLQDSQRSEKEFDAEMKTLGSVKNRNLVPLLGYCVANKE
RLLMYEYMANGYLYDQLHPADEESFKPLDWPSRLKIAIGTAKGLAWLHHSCNPRIIHRNISSKC
ILLTAEFEPKISDFGLARLMNPIDTHLSTFVNGEFGDFGYVAPEYSRTMVATPKGDVYSFGVVLL
ELVTGQKATSVTKVSEEKAEEENFKGNLVEWITKLSSESKLQEAIDRSLLGNGVDDEIFKVLKV
ACNCVLPEIAKQRPTMFEVYQLL
>At4g20140
IGSGGSGKVYKAELENGETVAVKKILWKDDLMSNKSFSREVKTLGRIRHRHLVKLMGYCSSKS
EGLNLLIYEYMKNGSIWDWLHEDKPVLEKKKKLLDWEARLRIAVGLAQGVEYLHHDCVPPIV
HRDIKSSNVLLDSNMEAHLGDFGLAKVLTENCDTNTDSNTWFACSYGYIAPEYAYSLKATEKS
DVYSMGIVLMEIVTGKMPTDSVFGAEMDMVRWVETHLEVAGSARDKLIDPKLKPLLPFEEDA
ACQVLEIALQCTKTSPQERPSSRQ
>At5g44700
IGSGGSGKVYKAELKNGETIAVKKILWKDDLMSNKSFNREVKTLGTIRHRHLVKLMGYCSSKA
DGLNLLIYEYMANGSVWDWLHANENTKKKEVLGWETRLKIALGLAQGVEYLHYDCVPPIVH
RDIKSSNVLLDSNIEAHLGDFGLAKILTGNYDTNTESNTMFAGSYGYIAPEYAYSLKATEKSDV
YSMGIVLMEIVTGKMPTEAMFDEETDMVRWVETVLDTPPGSEAREKLIDSELKSLLPCEEEAA
YQVLEIALQCTKSYPQERPSSRQASEYL
>At1g75820
IGKGGAGIVYRGSMPNNVDVAIKRLVGRGTGRSDHGFTAEIQTLGRIRHRHIVRLLGYVANKDT
NLLLYEYMPNGSLGELLHGSKGGHLQWETRHRVAVEAAKGLCYLHHDCSPLILHRDVKSNNIL
LDSDFEAHVADFGLAKFLVDGAASECMSSIAGSYGYIAPEYAYTLKVDEKSDVYSFGVVLLELI
AGKKPVGEFGEGVDIVRWVRNTEEEITQPSDAAIVVAIVDPRLTGYPLTSVIHVFKIAMMCVEE
EAAARPTMREVVHMLTNP
>At5g65700
IGKGGAGIVYKGVMPNGDLVAVKRLAAMSRGSSHDHGFNAEIQTLGRIRHRHIVRLLGFCSNH
ETNLLVYEYMPNGSLGEVLHGKKGGHLHWDTRYKIALEAAKGLCYLHHDCSPLIVHRDVKSN
NILLDSNFEAHVADFGLAKFLQDSGTSECMSAIAGSYGYIAPEYAYTLKVDEKSDVYSFGVVLL
ELVTGRKPVGEFGDGVDIVQWVRKMTDSNKDSVLKVLDPRLSSIPIHEVTHVFYVAMLCVEEQ
AVERPTMREVVQILTEI
>At3g49670
IGKGGAGIVYKGTMPKGDLVAVKRLATMSHGSSHDHGFNAEIQTLGRIRHRHIVRLLGFCSNH
ETNLLVYEYMPNGSLGEVLHGKKGGHLHWNTRYKIALEAAKGLCYLHHDCSPLIVHRDVKSN
NILLDSNFEAHVADFGLAKFLQDSGTSECMSAIAGSYGYIAPEYAYTLKVDEKSDVYSFGVVLL
ELITGKKPVGEFGDGVDIVQWVRSMTDSNKDCVLKVIDLRLSSVPVHEVTHVFYVALLCVEEQ
AVERPTMREVVQILTEI

>At4g20270
IGKGGRGIVYKGVMPNGEEVAVKKLLTITKGSSHDNGLAAEIQTLGRIRHRNIVRLLAFCSNKD
VNLLVYEYMPNGSLGEVLHGKAGVFLKWETRLQIALEAAKGLCYLHHDCSPLIIHRDVKSNNI
LLGPEFEAHVADFGLAKFMMQDNGASECMSSIAGSYGYIAPEYAYTLRIDEKSDVYSFGVVLL
ELITGRKPVDNFGEEGIDIVQWSKIQTNCNRQGVVKIIDQRLSNIPLAEAMELFFVAMLCVQEH
SVERPTMREVVQMIS
>At2g31880
LEIIGRGGCGEVFKAELPGSNGKIIAVKKVIQPPKDADELTDEDSKFLNKKMRQIRSEINTVGHI
RHRNLLPLLAHVSRPECHYLVYEYMEKGSLQDILTDVQAGNQELMWPARHKIALGIAAGLEY
LHMDHNPRIIHRDLKPANVLLDDDMEARISDFGLAKAMPDAVTHITTSHVAGTVGYIAPEFYQ
THKFTDKCDIYSFGVILGILVIGKLPSDEFFQHTDEMSLIKWMRNIITSENPSLAIDPKLMDQGFD
EQMLLVLKIACYCTLDDPKQRPNSKDVRTMLSQI
>At4g28490
IGFGSSGKVYKVELRGGEVVAVKKLNKSVKGGDDEYSSDSLNRDVFAAEVETLGTIRHKSIVR
LWCCCSSGDCKLLVYEYMPNGSLADVLHGDRKGGVVLGWPERLRIALDAAEGLSYLHHDCV
PPIVHRDVKSSNILLDSDYGAKVADFGIAKVGQMSGSKTPEAMSGIAGSCGYIAPEYVYTLRVN
EKSDIYSFGVVLLELVTGKQPTDSELGDKDMAKWVCTALDKCGLEPVIDPKLDLKFKEEISKVI
HIGLLCTSPLPLNRPSMRKVVIMLQEVSGAVPCSSPNTSKRSKTGGKLSPYYTEDLN
>At3g19700
IGRGGQGNVYKVSLRSGETLAVKHIWCPESSHESFRSSTAMLSDGNNRSNNGEFEAEVATLSNI
KHINVVKLFCSITCEDSKLLVYEYMPNGSLWEQLHERRGEQEIGWRVRQALALGAAKGLEYL
HHGLDRPVIHRDVKSSNILLDEEWRPRIADFGLAKIIQADSVQRDFSAPLVKGTLGYIAPEYAYT
TKVNEKSDVYSFGVVLMELVTGKKPLETDFGENNDIVMWVWSVSKETNREMMMKLIDTSIE
DEYKEDALKVLTIALLCTDKSPQARPFMKSVVSMLEKI
>At5g61480
LGMGSTGTVYKAEMPNGEIIAVKKLWGKNKENGKIRRRKSGVLAEVDVLGNVRHRNIVRLLG
CCTNRDCTMLLYEYMPNGSLDDLLHGGDKTMTAAAEWTALYQIAIGVAQGICYLHHDCDPVI
VHRDLKPSNILLDADFEARVADFGVAKLIQTDESMSVVAGSYGYIAPEYAYTLQVDKKSDIYSY
GVILLEIITGKRSVEPEFGEGNSIVDWVRSKLKTKEDVEEVLDKSMGRSCSLIREEMKQMLRIA
LLCTSRSPTDRPPMRDVLLILQE
>At5g46330
IGSSSLSTVYKGQLEDGTVIAVKVLNLKEFSAESDKWFYTEAKTLSQLKHRNLVKILGFAWESG
KTKALVLPFMENGNLEDTIHGSAAPIGSLLEKIDLCVHIASGIDYLHSGYGFPIVHCDLKPANILL
DSDRVAHVSDFGTARILGFREDGSTTASTSAFEGTIGYLAPEFAYMRKVTTKADVFSFGIIMMEL
MTKQRPTSLNDEDSQDMTLRQLVEKSIGNGRKGMVRVLDMELGDSIVSLKQEEAIEDFLKLCL
FCTSSRPEDRPDMNEILTHLMKL
>At5g20480
IGSGNFGNVFKGLLGPENKLVAVKVLNLLKHGATKSFMAECETFKGIRHRNLVKLITVCSSLDS
EGNDFRALVYEFMPKGSLDMWLQLEDLERVNDHSRSLTPAEKLNIAIDVASALEYLHVHCHDP
VAHCDIKPSNILLDDDLTAHVSDFGLAQLLYKYDRESFLNQFSSAGVRGTIGYAAPEYGMGGQP
SIQGDVYSFGILLLEMFSGKKPTDESFAGDYNLHSYTKSILSGCTSSGGSNAIDEGLRLVLQVGI
KCSEEYPRDRMRTDEAVRELISI
>At1g31420

IGCGGFGTVYKLAMDDGKVFALKRILKLNEGFDRFFERELEILGSIKHRYLVNLRGYCNSPTSK
LLLYDYLPGGSLDEALHVERGEQLDWDSRVNIIIGAAKGLSYLHHDCSPRIIHRDIKSSNILLDG
NLEARVSDFGLAKLLEDEESHITTIVAGTFGYLAPEYMQSGRATEKTDVYSFGVLVLEVLSGKR
PTDASFIEKGLNVVGWLKFLISEKRPRDIVDPNCEGMQMESLDALLSIATQCVSPSPEERPTMH
RVVQLLE
>At2g35620
IGCGGFGTVYKLSMDDGNVFALKRIVKLNEGFDRFFERELEILGSIKHRYLVNLRGYCNSPTSK
LLLYDYLPGGSLDEALHKRGEQLDWDSRVNIIIGAAKGLAYLHHDCSPRIIHRDIKSSNILLDGN
LEARVSDFGLAKLLEDEESHITTIVAGTFGYLAPEYMQSGRATEKTDVYSFGVLVLEVLSGKLP
TDASFIEKGFNIVGWLNFLISENRAKEIVDLSCEGVERESLDALLSIATKCVSSSPDERPTMHRV
VQLLE
>At2g26330
IGHGASSTVYKCVLKNCKPVAIKRLYSHNPQSMKQFETELEMLSSIKHRNLVSLQAYSLSHLGS
LLFYDYLENGSLWDLLHGPTKKKTLDWDTRLKIAYGAAQGLAYLHHDCSPRIIHRDVKSSNIL
LDKDLEARLTDFGIAKSLCVSKSHTSTYVMGTIGYIDPEYARTSRLTEKSDVYSYGIVLLELLTR
RKAVDDESNLHHLIMSKTGNNEVMEMADPDITSTCKDLGVVKKVFQLALLCTKRQPNDRPT
MHQVTRVL
>At5g62230
IGYGASSTVYKCALKSSRPIAIKRLYNQYPHNLREFETELETIGSIRHRNIVSLHGYALSPTGNLL
FYDYMENGSLWDLLHGSLKKVKLDWETRLKIAVGAAQGLAYLHHDCTPRIIHRDIKSSNILLD
ENFEAHLSDFGIAKSIPASKTHASTYVLGTIGYIDPEYARTSRINEKSDIYSFGIVLLELLTGKKAV
DNEANLHQLILSKADDNTVMEAVDPEVTVTCMDLGHIRKTFQLALLCTKRNPLERPTMLEVSR
VL
>At5g07180
IGYGASSTVYKCTSKTSRPIAIKRIYNQYPSNFREFETELETIGSIRHRNIVSLHGYALSPFG

supplementary Data Set 2   Amino acid sequences for motif analysis

>VMLRR-RLK210
MTQRSIAAGLPQDEVDALNQIITKMGASFGNFSADKACGDLVEQVQSTDPERSITCNCTFPNNT
CHITSLEIKRYSLPGQLPPELVQLPYLETIDFAYNYLNGSIPREWASMPLKSISLLANRLSGNIPSY
LENFTNLTYLNIELNQFSGTVPRELGKLVNLEKLILSSNKLSGNLPMELGDLNLTDFRINDNSLN
GSIPDFIQNWKQLGRLEIQASGLEGPIPSSISFLEKLTNLRISDINATNQLFPDLSNITDLKWLILKN
CNISGEIPPYIWQMSKVEILDLSFNNLNGELPDSILVGIWRVITLSGNSLSGKLPIWRQGINVDLS
YNNFTRESTEQPTCQQRDVMLNLFRSSSMGNDIGGACANDFRCDQYWYSMYINCGGENVNT
DDRTYEGDSVASGNDDWGLSSAGDFMDDNDFLNTDYTVNSPSLNIGELYRTARLSPLSLTYYH
RCLMNESYTVSLHFAEIQFTNDSTYNSLGRRIFDIYIQNDRVEKDFNIEVEANGVAMPIVKKYN
ATVTNNILEIRFFWAGKGTTRIPKSGVYGPLISAISVDPNFEPPSRGGKTKIAPIVVGVVGSCLILL
ALVVIGWRRYFRENNGRQKDLEGLESQTVSFTLKHIKAATKNFDPSNKIGEGGFGPVYKGLLP
DGTVIAVKQLSSKSSQGNREFLNEIGMISCLQHPNLVKLHGCCVEGNQLLLVYEYMENNSLAR
ALLGPEHSRIKLDWQTRQRICVGIARGLAFLHEESRLKIVHRDIKATNVLLDKHLNPKISDFGLA
KLDTEEKTHISTRIAGTMGYMAPEYALWGYLTHKVDVYSFGIVALEIVSGKHNMSHGPENNFA
CLLDWACHLQQSGNLMELVDEKLGSDFKKKEAERMIKASLLCTNGSPSLRPSMSEVVNMLEG
TAIIPDVIPEANNYNEDLRFKAIREHQKQIRSESSRGSQDHNSISIKSENEASSASAIDLHEINEVS
SMRYKARKDLKQMDSQVSTSFSITASSSASVHDPYDFNSNSQ
>VMLRR-RLK208
MTQRSIAAGLPQDEVDALNQIITKMGASFGNFSADKACGDLVEQVQSTDPERSITCNCTFPNNT
CHITSLEIKRYSLPGQLPPELVQLPYLETIDLAYNYLNGSIPREWASMPLKSISLLANRLSGNIPSY
LENFTNLTYLNIELNQFSGTVPRELGKLVNLEKLILSSNKLSGNLPMELGDLNLTDFRINDNSLN
GSIPDFIQNWKQLGRLEIQASGLEGPIPSSISFLEKLTNLRISDINATNQLFPDLSNITDLKWLILKN
CNISGEIPPYIWQMSKVEILDLSFNNLNGELPDSILVGIWRVITLSGNSLSGKLPIWRQGINVDLS
YNNFTRESTEQPTCQQRDVMLNLFRSSSMGNDIGGACANDFRCDQYWYSMYINCGGENVNT
DDRTYEGDVVASGTVSELYSSNDDWGLSSTGDFMDDDNFLKTDYTAISSSLNIDELYRTARLSP
LSLTYYHRCLMNESYTVSLHFAEIQFTNDSTYNSLGRRIFDIYIQNDRVEKDFNIEVEANGVVTP
IVKKYNATVTNNILEIRFFWASKGTTRIPGRGVYGPLISAISVDPNFEPPSRGGKTKIAPIVVGVV
GSCLILLALVVIGWRRYFRENNGRQKDLEGLESQTVSFTLKHIKAATKNFDPSNKIGEGGFGPV
YKGLLPDGTVIAVKQLSSKSSQGNREFLNEIGMISCLQHPNLVKLHGCCVEGNQLLLVYEYME
NNSLARALLGPEHSRIKLDWQTRQRICVGIARGLAFLHEESRLKIVHRDIKATNVLLDKHLNPK
ISDFGLAKLDTEEKTHISTRIAGTMGYMAPEYALWGYLTHKVDVYSFGIVALEIVSGKHNMSH
GPENNFACLLDWACHLQQSGNLMELVDEKLGSDFKKKEAERMIKASLLCTNGSPSLRPSMSEV
VNMLEGTAIIPDVIPEANNYNEDLRFKAIREHQKQIRSESSRGSQDHNSISIKSENEASSASAIDL
HEINEVSSMRYKARKDLKQMDSQVSTSFSITASSSASVHDPYDFNSNSQ
>VFLRR-RLK271
MTQRSIAAGLPQDEVDALNQIITKMGASFGNFSADKACGDLVEQVQSTDPERSITCNCTFPNNT
CHITSLEIKRYSLPGQLPPELVQLPYLETIDFAYNYLNGSIPREWASMPLKSISLLANRLSGNIPSY
LENFTNLTYLNIELNQFSGTVPRELGKLVNLKTLILSSNKLSGNLPMELGDLNLTDFRINDNSLN
GSIPDFIQNWKQLGRLEIQASGLEGPIPSSISFLEKLTNLRISDINATNQLFPDLSNITSLKWLILRN
CNMSGVIPSNIWQMSQLEILDLSFNKLHGELPNSILTGLWRVITLSGNSLSGKLPIWRQGINVDL
SYNNFTRESAEQPTCQQRDVMLNLFRSSSMGNDIGGACANDFRCDQYWYSMYINCGGENVN
TDDRTYEGDSVASGNDDWGLSSAGDFMDDNDFLNTDYTVNSPSLNIGELYRTARLSPLSLTYY
HRCLMNESYTVSLHFAEIQFTNDSTYNSLGRRIFDIYIQNDRVEKDFNIEVEANGVAMPIVKKY

NATVTNNILEIRFFWAGKGTTRIPKSGVYGPLISAISVDPNFEPPSRGGKTKIAPIVVGVVGSCLIL
LALVVIGWRRYFRENNGRQKDLEGLESQTVSFTLKHIKAATKNFDPSNKIGEGGFGPVYKGLL
PDGTVIAVKQLSSKSSQGNREFLNEIGMISCLQHPNLVKLHGCCVEGNQLLLVYEYMENNSLA
RALLGPEHSRIKLDWQTRQRICVGIARGLAFLHEESRLKIVHRDIKATNVLLDKHLNPKISDFGL
AKLDTEEKTHISTRIAGTMGYMAPEYALWGYLTHKVDVYSFGIVALEIVSGKHNMSHGPENNF
ACLLDWACHLQQSGNLMELVDEKLGSDFKKKEAERMIKASLLCTNGSPSLRPSMSEVVNMLE
GTAIIPDVIPEANNYNEDLRFKAIREHQKQIRSESSRGSQDHNSISIKSENEASSASAIDLHEINEV
SSMRYKARKDLKQMDSQVSTSFSITASSSASVHDPYDFNSNSQ
>VFLRR-RLK159
MQLKSISLLVNRLSGNVPSYLQNFTSLTYLNIEANQFSGPVPQELGKLVNLKSLILSSNNLSGNL
PVELSELKNLTDFRISDNNFNGSIPDFIQGWQQLDRLEMEAGGLKGPIPSSTSVLKNLTDLRISEL
NGTTQAFPNLSSITGLRRLVLRNCNISGEIPAYIWGMNDLRTLDLSFNKLSGRLPTSINTANLLFL
FLSHNMLNGDIPIIRKGIDVDLSYNNFTKQSPARPACQQTREMNLNLFRSSSMRNDLGGACME
DFRCDRYWHNLYINCGGFTEIINGSTYEGDADDAGGAARFHQNSNNWGFSSTGDFLGDNNDR
NLDSNIISLSSSNLDGLYRTARISPLSLTYYRYCLENGSYSVSLHFAEIQFTEDKLYNRIGRRLFDI
YIQDNLVEKDFNTKTEASAVLTPITKSYNANVSNGTLEIRFYWSGKGTTRIPRIGVYGPIISAISV
NSNFKPQSGGRKTKVVPIVIGVVGSCLIFSVLGILWWRRCSKAKSKRQKELEGLEFQTASFTLK
QIKAATNNFDHANKIGEGGFGPVYKGLLADGTAIAVKQLSSKSSQGNX
>VMLRR-RLK178
MQLKSISLLVNRLSGNVPSYLQNFTSLTYLNIEANQFSGPVPQELGKLVNLKSLILSSNNLSGNL
PVELSELKNLTDFRISDNNFNGSIPDFIRSWQQLDRLEMEASGLKGPIPSSTSVLKNLTDLRISEL
NGTTQAFPNLSSITGLRRLVLRNCNISGEIPAYIWGMNDLRTLDLSFNKLSGKLPTSINTANLLFL
FLSHNMLNGDIPIIRKGIDVDLSYNNFTKQSPARPACQQTREMNLNLFRSSSMRNDLGGACME
DFRCDRYWHNLYINCGGFTEIINGSTYEGDADDAGGAARFHQNSNNWGFSSTGDFLGDNNDR
NLDSNIISLSSSNLDGLYRTARISPLSLTYYRYCLENGSYSVSLHFAEIQFTEDKLYNRIGRRLFDI
YIQDNLVEKDFNTKTEASAVLTPITKSYNANVSNGTLEIRFYWSGKGTTRIPRIGVYGPIISAISV
NSNFKPQSGGRKTKVVPIVIGVVGSCLIFSVLGILWWRRCSKAKSKRQKELEGLEFQTASFTLK
QIKAATNNFDHANKIGEGGFGPVFKGLLADGTVIAVKQLSSKSSQGNX
>VFLRR-RLK255
XQDLAGILPRAIVKLPYLNYLDLTRNYLSGNIPSEWISMKLEYLILSVNRLTGQIPSYLGNIFTLK
GLSLENNMFSGTVPHQLGNLVNLEIFVLSANNLTGTLPVALTNLTKLTELRISSNKFIGKIPSFFQS
WKHLQRLEIQASGLEGPIPSSISALNNLIELRISDIQGEGSEFPHLENMTGMNKLMLRNCNISGP
VPQYITAMPNLKILDLSFNRMGGNLSADFKGLTTLENLYLTSNLLSGPIPDWLLNGDTRDAIDLS
RNNFTENSAPSTCRTTLNLFKSTSGGNNSKVAEYSLHINCGGGTTKIGSVTYEADEESGDAAKY
VPSKESWEISNTGHFWDKVRSSSDYIAQNSSILRMKNSELYTTARLSPLSLSYYARCLANGNYS
VKLHFAEIVMRDNRSFYSLGRRIFDVYIQENRVLKDFNIVEEAQGVDKVIIRDFKAIVKDGTLEI
RFHWAGKGTTATPKRGIYGPLISAIDVESDFKPTVSIEAKKKKFIVAGAVVLPLFLILIITGTLWW
KGYLGGRAVKEQELLGLDLHTGIFTFRQIKAATNNFNPANKLGAGGFGAVYKGTLKDGTIIAV
KQLSSRSKQGNREFLNEIGMISVLQHPNLVRLYGCCVEGNQLLLVYEYMENNSLAHVLFDAEE
SQSKLDWATRQRICVGIAKGLAFLHEESALKIVHRDIKTANILLDGDLNPKISDFGLAKLDEEEN
THISTR
>VMLRR-RLK172
MRADLANPNPKLLLLLTICFITFFSFAASTSKLHPTEVKALKDIGKKLGKKDWNFGKDPCSGEG
NWSILDEIKGFESSVICNCRFNHNSTCHVVSIALKAQNLSGIVPPEFSKLRYLELLDLSRNCLTGS

IPSQWATMRLVDLSFMGNRLSGSFPKVLTNISTLKNLSIEGNSFSGSIPPEIGKLIHLHKFILSSNA
FTGKLPAEFSKLANLMDMRINDNNFSGKIPGFISKWTQIQKLHIQGCGLEGPIPPSISVLTSLTDL
RITDLKGKGSTFPPLSDMKSMKTLILRNCLLSGDIPEYIGHMKKLKNLDLSFNNLIGEIPASFSQL
AKADFMYLTSNKLTGTVPVWVLERNKNVDISDNNFTWESSSPIECPRGSVNLVESYASSTEKLS
RVHSCLKQNFPCSSSNKKYYSLHINCGGKEIIASGNTTYQADLEPRGASMFYSSQSWAFSSTGN
FLDNDIEADSYIQMNTSAISNVSALDAQLYTTARTSPLSLTYYGLCLINGNYTVKLHFAEIVFIN
DSSLNSLGKRIFDVYIQEKLVLKDFNIVEDAGGTGRPIVKTFTVAVTSHTLKIHFYWAGKGTTGI
PLRGVYGPLISAISVDPNFKTPSDHNDEKNVIIVSSTVGGTVFIVLLILCIMWRKGCLGGKVSAD
KELRGLDLQTGIFTLRQIKAATKNFDAANKVGEGGFGSVYKGLLSDGTVIAVKQLSSKSKQGN
REFVNEIGMISALQHPNLVKLLGCCTEDNQLLLVYEYMENNSLAHALFGSEELRLKLNWPIRF
KICLGIAKGLAFLHEESKLKVVHRDIKPTNVLLDKDLNAKISDFGLAKLYEAEKTHVITRIAGTT
GYMAPEYAMRGYLTNKADVYSFGVVALEIVSGKNGTSYRPNDESVYLLDLAYVLQEKGDLLS
LVDPMLGYDYSVKQAMTILDLAMLCTNPSPTLRPTMSEVVNVLEGKSKIKAPSFHVPYSTDDF
AMAKAVASLIPRIRSGSSSSGKTSNPVTYCWSRDEGEYGIYEDCSSEMLEMTDSLVKLSS
>VMLRR-RLK215
MTKLKNLDLSFNKLTGDIPRSFSGAVKTDYIYLTGNMLTGVVPDWILQKGENVDLSYNNFTTH
SSCQERTVNLFGSSSMNNVSTTVSCLRSNHCPKNFYSFHINCGGKEAVINEKTYEDDTDSGGPS
RFYQSRTNWAFSSTGNFMDDDRPTDSFTWTNTAKPGTSALYTDARLSPISLTYYGFCMGNGNY
TVSLHFAEIMFTDDNTYNSLGRRIFDIYIQGKRVQKDFNLGVEAGGAGKAVKNFAAIVTNQTLE
IRFYWNGKGTTAIPSRGVYGPLISAISVNPDFVPPSENGSSISAGTVVGIVVAVVAIIFVVLGVLW
WKGCLRRKDTMNQDLKGLDLQTGSFTLKQIKAATNNFDRANKIGEGGFGPVYKGLLSDGTIIA
VKQLSSKSKQGNREFVNEIGMISALQHPHLVKLYGCCIEGNQLLLVYEYMENNSLARALFGKS
NKHNLFLHFHDACTSEKYIIIAVFLCSLLIGDLKGYEEHQLNLDWSTRHKICVGIARGLAFLHEE
SRLKIVHRDIKATNVLLDKYLNSKISDFGLAKLDEEENTHISTRVAGTFGYMAPEYAMRGYLTD
KADVYSFGIVALEIVSGRCNTSNRSNMKEECFYLLDWACVLKEKGNLLELVDPKLGKNYNKE
QVMTMINVALLCTNVSPVVRPAMSSVVSMLEGKASVQNLALDTSVSNDEMRIKAMRKHFQD
SIEHSTSESLTQSMALDGPWTGSSTSAKDLYPISLGSDYWESRN
>VMLRR-RLK209
XFSFEAAQLPNDEVEALRDIAKILGKRDWNFSVDPCSGELGWADLNPVEGQENAVKCNCSFSN
DTICHVVSIILKAQNLQGTLPADLGRFPFLQEIDLTRNYLNGTIPPEWGSTQLVNISLMGNRLTGP
IPKELGNISTLANITVEFNKLSGELPQELGNLSRIERMLLTSNNFTGQLPPTFAKLTTLQDFRIGD
NQFIGQIPDFIQNWTNLEKLVIQGSGLSGPIPSGIRLLGSITDLRISDLSNGTEVPFPTLSNMKNLK
TLILRSCNIVGQLPDFLGEMTKLKNLDLSFNKLTGDIPRTFSGAVKTDYIYLTGNMLTGVVPDWI
LQKGENVDLSYNNFTTHSSCQERTVNLFGSSSMNNVSTTVSCLRSNHCPKNFYSFHINCGGKE
AVINGKTYEDDTDSGGPSRFYQSRTNWAFSSTGNFMDDDRPTDSFTWTNTAKPGTSALYTDAR
LSPISLTYYGFCMGNGNYTVSLHFAEIMFTDDNTYNSLGRRIFDIYIQGKRVQKDFNLGVEAGG
AGKAVKNFAAIVTNQTLEIRFYWNGKGTTAIPSRGVYGPLISAISVNPDFVPPSENGSSISAGTV
VGIVVAVVAIIFVVLGVLWWKGCLRRKDTMNQDLKGLDLQTGSFTLKQIKAATNNFDPANKIG
EGGFGPVFKGLLSDGTIIAVKQLSSKSKQGNREFVNEIGMISALQHPHLVKLYGCCIEGNQLLLV
YEYMENNSLARALFGPEEHQLNLDWSTRHKICVGIARGLAFLHEESRLKIVHRDIKATNVLLD
KYLNSKISDFGLAKLDEEENTHISTRVAGTFGYMAPEYAMRGYLTDKADVYSFGIVALEIVSGR
CNTSNRSNMKEECFYLLDWACVLKEKGNLLELVDPKLGKNYNKEQVMTMINVALLCTNVSP
VVRPAMSSVVSMLEGKASVQNLALDTSVSNDEMRIKAMRKHFQDSIEHSTSESLTQSMALDG
PWTGSSTSAKDLYPISLGSDYWESRN

>VFLRR-RLK258
XFSFEAAQLPNDEVEALRDIAKILGKRDWNFSVDPCSGESGWADLNPVEGQENAVKCNCSFSN
DTICHVVSIILKAQNLQGTLPADLGRFPFLQEIDFTRNYLNGTIPPEWGTTQLVNISLMGNRLTG
PIPKELGNISTLANITVEFNKLSGELPQELGNLSRIERMLLTSNNFTGQLPPTFAKLTTLQDFRIGD
NQFTGQIPDFIQNWTNLEKLVIQGSGLSGPIPSGIRLLGSITDLRISDLSNGTEIPFPTLSNMKNLK
TLILRSCNIVGQLPDFLGEMTKLKNLDLSFNKLTGDIPRSFSGAVKTDYIYLTGNMLTGVVPDWI
LQKGENVDLSYNNFTTHSSCQERTVNLFGSSSMNNVSTTVSCLRSNHCPKNFYSFHINCGGKE
AVINEKTYEDDTDSGGPSRFYQSRTNWAFSSTGNFMDDDRPTDSFTWTNTAKPGTSALYTDAR
LSPISLTYYGFCMGNGNYTVSLHFAEIMFTDDNTYNSLGRRIFDIYIQGKRVQKDFNLGVEAGG
AGKAVKNFAAIVTNHTLEIRFYWNGKGTTAIPSRGVYGPLISAISVNPDFVPPSENGSSISAGTV
VGIVVAVVAIIFVVLGVLWWKGCLRRKDTMNQDLKGLDLQTGSFTLKQIKAATNNFDPANKIG
EGGFGPVFKGLLSDGTIIAVKQLSSKSKQGNREFVNEIGMISALQHPHLVKLYGCCIEGNQLLLV
YEYMENNSLARALFGPEEHQLNLDWSTRHKICVGIARGLAFLHEESRLKIVHRDIKATNVLLD
KYLNSKISDFGLAKLDEEENTHISTRVAGTFGYMAPEYAMRGYLTDKADVYSFGIVALEIVSGR
CNTSNRSNMKEECFYLLDWACVLKEKGNLLELVDPKLGKNYNKEQVMTMINVALLCTNVSP
VVRPAMSSVVSMLEGKASVQNLALDTSVSNDEMRIKAMRKHFQDSIEHSTSESLTQSMALDG
PWTGSSTSAKDLYPISLGSDYWESRN
>VFLRR-RLK261
XFSFEAAQLPNDEVEALRDIAKILGKRDWNFSVDPCSGELGWADLNPVEGQENAVKCNCSFSN
DTICHVVSIILKAQNLQGTLPADLGRFPFLQEIDFTRNYLNGTIPPEWGSTQLVNISLMGNRLTGP
IPKELGNISTLANITVEFNKLSGELPQELGNLSRIERMLLTSNNFTGQLPPTFAKLTTLQDFRIGD
NQFTGQIPDFIQNWTNLEKLVIQGSGLSGPIPSGIRLLGSITDLRISDLSNGTEIPFPTLSNMKNLK
TLILRSCNIVGQLPDFLGEMTKLKNLDLSFNKLTGDIPRSFSGAVKTDYIYLTGNMLTGVVPDWI
LQKGENVDLSYNNFTTHSSCQERTVNLFGSSSMNNVSTTVSCLRSNHCPKNFYSFHINCGGKE
AVINEKTYEDDTDSGGPSRFYQSRTNWAFSSTGNFMDDDRPTDSFTWTNTAKPGTSALYTDAR
LSPISLTYYGFCMGNGNYTVSLHFAEIMFTDDNTYNSLGRRIFDIYIQGKRVQKDFNLGVEAGG
AGKAVKNFAAIVTNHTLEIRFYWNGKGTTAIPSRGVYGPLISAISVNPDFVPPSENGSSISAGTV
VGIVAAVVAIIFVVLGVLWWKGCLRRKDTMNQDLKGLDLQTGSFTLKQIKAATNNFDPANKIG
EGGFGPVFKGLLSDGTIIAVKQLSSKSKQGNREFVNEIGMISALQHPHLVKLYGCCIEGNQLLLV
YEYMENNSLARALFGPEEHQLNLDWSTRHKICVGIARGLAFLHEESRLKIVHRDIKATNVLLD
KYLNSKISDFGLAKLDEEENTHISTRVAGTFGYMAPEYAMRGYLTDKADVYSFGIVALEIVSGR
CNTSNRSNMKEECFYLLDWACVLKEKGNLLELVDPKLGKNYNKEQVMTMINVALLCTNVSP
VVRPAMSSVVSMLEGKASVQDLALDTSVSNDEKRIKAMRKHFQDSIEHSTSESLTQSMSLDGP
WTGSSTSAKDLYPISLGSDYWEX
>VFLRR-RLK275
MFFNRYPSSWILVFLVLYGVCITDIVHAQNGTQATTDPNEARALNSIFQQWGIKADQTQWNVS
GELCSGAALGSSPTIDAQNYNPFIKCDCSDNNATTCHITALKVYAIDVVGVIPDELWTLTFLTNL
KLGQNYLTGTLSPSIGNLTRMQYLDVSINSLSGELPKELGLLTDLRSFGFGSNNFSGPLLSEIGNC
SKLEQIYFDSSGVSGEIPLTYANLRNMVTVWASDNELTGRIPEFIGNWSKLAVLRFEGNSFEGPIP
SALSNITSLTELRISGLSNGSSSLAFLKDMKSLTILVLKNNNISDSIPSNIGEYQNLTQLDLSFNNIT
GQIPDSLFNLSNLNVLFLGNNKLDGPLPSQKVATLQNIDVSYNNLAGSFPSWVNDQNLQVNLV
ANNFTIDSSNSSGLPSGLNCLQRNFPCNRGSPVYSQFAIKCGGPPITSLNGIEYERENETLGPATY
YVTGTSRWGVSNVGIFTGNNNPQYTASSSSQFTNTLDSELFQTARVSASSLRYYGLGLENGNY
TVSLQFAETVIEDGNTWRSLGRRIFDVYVQGNRVLKDFDIKKEAGGVSKRAVERSYNAVVSEN

YLEIHLFWAGKGTCCIPFQGTYGPFISAISATPDFVPTVSNRPPTRKKDRTGLIVGIVVGVGVASF
LLVFVVFFVIRRRKRQSTYDDEELLGIDSKPYTFSYTELKTATEDFSPANKLGEGGFGPVYKGIL
NDGRVIAVKQLSVASHQGKSQFVTEIATISAVQHRNLVKLYGCCIEGYNRLLVYEYLENKSLDQ
ALFGETSLNLDWPTRYQICLGVARGLAYLHEESRLRIVHRDVKASNILLDSELIPKISDFGLAKL
YDDKKTHISTRVAGTIGYLAPEYAMRGHLTEKADVFAFGVVALEVISGRPNSDSSLEEEKIYLLE
WAWNLHENNREVELVDSKLSDFSEEDVKRLIGVALLCTQTSPNLRPSMSRVIAMLSGDTEVST
VTSRPGYLTDWKFDDTSTFMSDDAGKGNDTSYYSSSQSTSMVADAEHTHKSAAKPLLQEITGE
GR

>VFLRR-RLK220

XVEALNKIFKQWDIQSVPLWNISGQPCSGTAIDESEFEAPDNNPAIRCDCSYNNGTTCHIVRLRV
YNLNKRGVIPEEILVFKHLIFLKIDRNYFTGPLPAFIGNLTALRTLSIAHNAFSGTIPRELGNLKDL
TLFSIGVNDFSGTLPPELGNLVKLEQMYINSCGLSGEIPSTFANLTRMRILWAFDNPFTGKIPDFIR
NWTQLTTLRLQGNSFEGPIPSSFSNLVSMSSLRISDISNLSSTLDFIKNMKNLTDLNLRNALITGTI
PPNIEEYQALKILDLSFNNLTGQIPSALFSLRSLESLFLGNNSLSGALPNQKSGLLQTIDLSYNHL
TGSFPSWVNSNLELNFVANNFVFDNSNISVLPRLNCLQRNFPCNRNTPRYANFAIKCGGPEMRT
SGILFEAENSSLGAASFNVTRSEKWAVSITGMFADRQNPTYVEDTLSQVTSTNTPELYLTSRLSP
GSIRYYGLGLENGPYTISLFFAETALKHRSSQIWESVGRRVFDIYIQGSLELKDFDISKEAGGVER
AITKTFNATVSENHLEIHLFWAGKGTCCTPIQGYYGPIISALSVVPDFIPTVSGIPPGSRQKSRQG
LIISISISAGVISSILIFAIFYIKRRARKNDEEVLSGLGPRPYTFSYAQLRMATEDFSPSNQLGEGGY
GPVYKGTLSDGRKVAVKLLSLASNQGKSEFVTEIATISAVQHRNLVKLYGCCIEGNRRLLVYEY
LENKSLDKALFATCAGKTSLHLDWPTRFDICLGTARGLAYLHEESRPRIVHRDVKAX

>VFLRR-RLK248

XVEALNKIFEQWDIQSVPLWNISGKPCSGTAIDESEFEAPDNNPAIRCDCSYNNGTTCHIVRLRV
YNLNKRGVIPEEILVFKHLIFLKIDRNYFTGPLPAFIGNLTALRTLSIAHNAFSGTIPRELGNLKDL
TLFSIGVNDFSGTLPPELGNLVKLEQMYINSCGLSGEIPSTFANLTRMRILWAFDNPFTGKIPDFI
GNWTQLTTLRLQGNSFEGPIPSSFSNLVSMSSLRISDLSNVSSTLDFIKNMKNLTDLNLRNALIT
GTIPPNIEEYQALKILDLSFNNLTGQIPSALFSLRSLESLFLGNNSLSGALPNQKSGLLQTIDLSYN
HLTGSFPSWVNSNLELNFVANNFVFDNSNISVLPRLNCLQRNFPCNRNTPRYANFAIKCGGPEM
RTSGILFEAENSSLGAASFNVTRSEKWAVSITGMFADRQNPTYVEDTLSQVTSTNTPELYLTSRL
SPGSIRYYGLGLENGPYTISLFFAETALKHRSSQIWESVGRRVFDIYIQGSLELKDFDISKEAGGV
ERAITKTFNATVSENHLEIHLFWAGKGTCCTPIQGYYGPIISALSVVPDFIPTVSGIPPGSRQKSR
QGLIISISISAGVISSILIFAIFYIKRRARKNDEEVLSGLGPRPYTFSYAQLRMATEDFSPSNQLGEG
GYGPVYKGTLSDGRKVAVKLLSLASNQGKSEFVTEIATISAVQHRNLVKLYGCCIEGNRRLLVY
EYLENKSLDKALFATCAGKASLHLDWPTRFDICLGTARGLAYLHEESRPRIVHRDVKASNILLD
AELCPKISDFGLAKLYDEKKTHISTRVAGTIGYMAPEYAMRGHLTEKADVFSFGVLALEILSGIP
NYENNLVEEKIYLLGWAWTLYEKNQSLALVDPSLMGFDENEALRVLRVALLCTQASPTVRPTM
SRVVAMLAGDIEVSVAITKPSYLSDWDPKDIHCGFSYEDTKTFTKSEICSNKSHESHPIDLNPGV
DLTPSPLNFSEPRLSDLIGKGX

>VFLRR-RLK256

MICEYRLMSLITLPLLLLVCKVGSEFRIVNAVLPPLTGNTGNPYNRRDPSSVACNKAYSRYCLGR
EYRQKLDCATDGGLPCYEVYALRAVTRALRLPSMPYISREYCNDGKSEDSITVGCNCTEKNGT
VCHITRISTSSIDLSGGRIHENVSHLTYLKTLDLSYNHLHCTIPESLGNLKSLVTLNLHNNFLNGS
IPSSFQGLSSLKTLSLRNNKLSGPIPKELGSLSNLVTMRLEENELYGHLPPELGKLSKLERLDLSS
NNLTGNLPRTYDKLTSLEIFGVAGNSLNGQIPEFISKWTNLRELYLIGNDFEGHLPLEIFNMSKLD

MLWVSDLRNPGFSFPEHANMTNIENLILRNCNIIGPVPEYIANWIWLLTGNMLDGPIPPLIDND
MDLSYNNILLPKDAKEGKPHGSGPKPHGPEPNTKPNRNYILEARDKYCQGKSKYYSLFINCGG
GQTSFEGNQYDEDINETKFYESPGGKWAYSCSGDFLSASADSSDYLKNMTCGVSEQSLHETAR
LCPTSLTYYGFCLHKGNYTVKLHFAEIVYTKDEDYSSSGKRIFDVYIQGEKKLENFNIKDEAGW
PNKLHTREFVTHVDENPLVIHFFWAGKGSIYNTPQLNGPLVSAISVTPNFTIPEGNKLSTSKIVGI
AVGSAFAPILLLAFLWKMGWLGNKELQEVHIKVRKKSFTLQQIIDGTRDFSSKMEIGRGRFGIV
YKAEMPDQTKLAVKKISPESTQDKLKDELQGEIFFNVKSLEHENLIQLFDGYSKKDLHLLIYEY
MENGSLDQALFEPKSRIELNWEVRFNICLGLAKALKYLHEENVRKIIHRNIKASNILLDENYTA
KLTDFGLASFDNEDDPFKTIKAGGARAYFAPEYARGQAITDKADVYSFGVVTLEIFSGRSCTEN
KSEAQKYLLDDACIFQARGKIDQLVDKKLANYDRKLVPKILNLAIMCINPTADLRPTMSEVVS
VLSGGTLEQISKVHTSSENFSGYYLX

>VMLRR-RLK202

MICEYRLMSLITLPLLLLVCKVGSEFRIVNAVLPPLTGNTGNPYNRRDPSSVACNKAYSRYCLGR
EYRQKLDCATDGGLPCYEVYALRAVTRALRLPSMPYISREYCNDGKSEDSITVGCNCTEKNGT
VCHITRISTSSIDLSGGRIHENVSHLTYLKTLDLSYNHLHCTIPESLGNLKSLVTLNLHNNFLNGS
IPSSFQGLSSLKTLSLRNNKLSGPIPKELGSLSNLVTMRLEENELYGHLPPELGKLSKLERLDLSS
NNLTGNLPRTYDKLTSLEIFGVAGNSLNGQIPEFISKWTNLRELYLIGNDFEGHLPLEIFNMSKLD
MLWVSDLRNPGFSFPEHANMTNIENLILRNCNIIGPVPEYIANWIWNYILEARDKYCQGKSKY
YSLFINCGGGQTSFEGNQYDEDINETKFYESPGGKWAYSCSGDFLSASADSSDYLKNMTCGVS
EQSLHETARLCPTSLTYYGFCLHKGNYTVKLHFAEIVYTKDEDYSSSGKRIFDVYIQGEKKLEN
FNIKDEAGWPNKLHTREFVTHVDENPLVIHFFWAGKGSIYNTPQLNGPLVSAISVTPNFTIPEGN
KLSTSKIVGIAVGSAFAPILLLAFLWKMGWLGNKELQEVHIKVRKKSFTLQQIIDGTRDFSSKM
EIGRGRFGIVYKAEMPDQTKLAVKKISPESTQDKLKDELQGEIFFNVKSLEHENLIQLFDGYSK
KDLHLLIYEYMENGSLDQALFEPKSRIELNWEVRFNICLGLAKALKYLHEENVRKIIHRNIKAS
NILLDENYTAKLTDFGLASFDNEDDPFKTIKAGGARAYFAPEYARGQAITDKADVYSFGVVTLE
IFSGRSCTENKSEAQKYLLDDACIFQARGKIDQLVDKKLANYDRKLVPKILNLAIMCINPTADL
RPTMSEVVSVLSGGTLEQISKVHTSSENFSGYYL

>VMLRR-RLK216

MGNNGSIILIPILIFLLRWQLVSGAGPAGNTYSGDPDGKYGSGNPYSGYPNGQGFGLSGKLQAT
YCGQNQTLPCAEVNALQAVINGFRLLPTIYISSTYCGYRLTTINCTCYRDDKNFILCHITEIYMSS
KDLSGYIDPAISGLQFLKILELSNNQLTGSIPASLGNLTNLERLYIINNQLVGSIPESFKNLKSLKIL
DLSSNYLDGPIPSLRDLQNLNTIGLRFNFLNGPIPASLGNLSSLQILNLYSNILSGPIPDDLGNLTQ
LQFLALDDNELNGKLPEAFGKLSNITALWLGSNYISGNIPQNYSYLTGLQIFSVAGNDLSGKIPD
YIAKWKNLTALILLGNNFDGNLPEGIFKLEKLQTLWVSGLTNPGFAFPKQEKLTTNLYSLILRNC
SINGTIPSYIAQWLSLTNLDLSFNNLIGEIPKFSPYLKKIFLTRNKLNGTLPPWIINEHNAPERTND
VALMDLSNNDFANVPSVNHENGTLSDSPNSIIEPRSEYISQKIKECKVKYRSLSINSGGEAVKFG
KQNYENDTALSNFYLSPQGNWAYSFSGDYISPTINASGYIKNLTCGVSLPEAQLYVNARVAPVS
LTYYAFCLHQGKYNVELHFAETLYSKKEDHSKVGKRVFDVHIQGKRVLQDFNIKKEAGDANK
NVMTRFPATVAKNKPLSIEFFWTGRGSLYNPPGQNGPLISAISITRAPRKLSLWEIVVIVAACVLG
LLILLAFMWRMGWIGERELRKARIELETQDGKKSFTVKEIIDATGNFSPRKKIGKDGRFGIIYKA
ELSNLTVAVKKLFPQSKAVAQIGTEVYAKTFKLEHDNLVQLLATYSRKDLHLLIYEYMDRGSLE
QALFDPKPSMQLSWKTRYSICLQIAQGLEYLHAKNPPIIHRDIKASNILLDGSSKAKISDFGLAK
LYEEDNPFMFIEEGGTRMYMAPEYATHKAVTGVDVFSFGVLLLEIVTGIKNDENLSKHHDQTI

FLLEKVANLHAMVKDAKLQYKKVKYAELVDEKLLNSNYSEEEVVTIIDLAMLCTDQMASLRP
IISDVVSVLEGKEIVENVSKIK
>VMLRR-RLK220
MDFSRNYLNGPIPTKFGGLQNLTFMDLSSNILSGSIPEELGKLTQLYRLILDNNELNGSLPEGLG
NLTNLGTLRLSSNNLTGSIPQSYFNLTQLEIFRVGGNYLSGGIPDFIADWGNLEELILVGNNFEGN
IAEGIFNLPKLEKLWVVGLRNPGLTFPKKANMSSLQTLTLRNCSINGTIPSYIAQWLSLTNLDLS
FNNLIGEIPKFSPYLKKIFLTRNKLNGTLPPWIINEHNAPERTNDVALMDLSNNDFANVPSVNHE
NGTLSDSPNSIIEPRSEYISQKIKECKVKYRSLSINSGGEAVKFGKQNYENDTALSNFYLSPQGN
WAYSFSGDYISPTINASGYIKNLTCGVSLPEAQLYVNARVAPVSLTYYAFCLHQGKYNVELHFA
ETLYSKKEDHSKVGKRVFDVHIQGKRVLQDFNIKKEAGDANKNVMTRFPATVAKNKPLSIEFF
WTGRGSLYNPPGQNGPLISAISITRAPRKLSLWEIVVIVAACVLGLLILLAFMWRMGWIGEREL
RKARIELETQDGKKSFTVKEIIDATGNFSPRKKIGKDGRFGIIYKAELSNLTVAVKKLFPQSKAVA
QIGTEVYARTFKLVHDNLVQLLATYSRKDLHLLIYEYMDRGSLEQALFDPKPSMQLSWKTRYSI
CLQIAQGLEYLHAKNPPIIHRDIKASNILLDGSSKAKISDFGLAKLYEEDNPFMFIEEGGTRMYM
APEYATHKAVTVGVDVFSFGVLLLEIVTGIKNDENLSKHHDQTIFLLEKVANLHAMVKDAKLQ
YKKVKYAELVDEKLLNSNYSEEEVVTIIDLAMLCTDQMASLRPIISDVVSVLEGKEIVENVSKI
KVKESSTV
>VFLRR-RLK233
MNHGKSVQVKNPDGSLVLQYQRRRDFPIDNKKYCYNLGTKERRRYLVRATFQYGSLENEDSY
PKFDLYLDATKWSTVTVLDASRVYVEEMIIRAPSSSIDVCICCATTGSPFISTIELRPLNLSMYAT
DYEDGFFLKLAARVNFGAPSEEALRYPDDPYDRIWDSDLVKRQNFLVGVAPGTVRINTTKSIDI
QTREYPPVKVMQTAVVGEEGMLSYRLNLEDFPANARAYAYFAEIEDLRANETRKFKMMQPYIS
DYSNAVVNIAENANGSYRLYEPSYMNVTLDFVLSFSFVKTHDSTQGPLLNAIEISKYLKIASKT
DSQDVTVLDAFQSMLAASSLTNEGGDPCVPAQWDWVNCSSNAPPRITKIALSGKNLKGEIPPEI
NNMEALEELWLDGNFLTGSLPDISNLVNLKIVHMEKNKLSGSLPKYLGSLPNLRELYIQNNSFT
GEVPPALLNGKVIINYEDNPGLHKKATKMLHFKLILGISIGILAVLLVLLLGSLVYLRSLQRKAS
HQKTNDQGNSLRNSTKPSTAYSIARGWHLMDEGVSYYISFSELEEATNNFSKKIGKGSFGTVY
YGQMKDGKEIAVKIMADSSSHLRQQFVTEVALLSRIHHRNLVPLIGFCEEEHQRCNPSIIHRDVK
TSNILLDINMRAKVSDFGLSRQAEEDLTHISSVARGTVGYLDPEYYANQQLTEKSDVYSFGVVL
LELISGKKPVSTEDFGAEMNIVHWARALIRKGDVVSIVDSIIIGNAKIESIWRIAEVAIQCVQQRA
ISRPKMQEVILAIQEAIKIEKGTDSSQKLSASGSSKAQSSRKTLLTSFLEIESPDLSNGCLVPAAR
>VMLRR-RLK179
MNHGKSVQVKNPDGSLVLQYQRRRDFPIDNKKYCYNLGTKERRRYLVRATFQYGSLENEDSY
PKFELYLDATKWSTVTVLDASRVYVEEMIIRAPSSSIDVCICCATTGSPFISTIELRPLNLSMYATD
YEDGFFLKLAARVNFGAPSEEALRYPDDPYDRIWDSDLVKRQNFLVGVAPGTVRINTTKSIDIQ
TREYPPVKVMQTAVVGEEGMLSYRLNLEDFPANARAYAYFAEIEDLRANETRKFKMMQPYISD
YSNAVVNIAENANGSYRLYEPSYMNVTLDFVLSFSFVKTHDSTQGPLLNAIEISKYLKIASKTDS
QDVTVLDAFQSMLAASSLTNEGGDPCVPAQWDWVNCSSNAPPRITKIALSGKNLKGEIPPEIN
NMEALEELWLDGNFLTGSLPDISNLVNLKIVHMEKNKLSGSLPKYLGSLPNLRELYIQNNSFTG
EVPPALLNGKVIINYEDNPGLHKKATKMLHFKLILGISIGILAVLLVLLLGSLVYLRSLQRKASH
QKTNDQGNSLRNSTKPSTAYSIARGWHLMDEGVSYYISFSELEEATNNFSKKIGKGSFGTVYY
GQMKDGKEIAVKIMADSSSHLRQQFVTEVALLSRIHHRNLVPLIGFCEEEHQRILVYEYMHNGT
LRDHIHGLRTIILELSSLF
